# Histamine H1 receptor inverse agonists improve structure and pain in an osteoarthritis mouse model

**DOI:** 10.1172/JCI183588

**Published:** 2025-08-28

**Authors:** Ichiro Kurakazu, Merissa Olmer, Hannah Swahn, Kevin Myers, Chelsea Kenvisay, Yukio Akasaki, Yasuharu Nakashima, Martin K. Lotz

**Affiliations:** 1 Department of Molecular and Cellular Biology, Scripps Research, La Jolla, California, USA.; 2 Department of Orthopaedic Surgery, Graduate School of Medical Sciences, Kyushu University, Fukuoka, Japan.

**Keywords:** Aging, Bone biology, Cell biology, Calcium signaling, Cartilage, Osteoarthritis

## Abstract

Osteoarthritis (OA) is the most common joint disease. Controlling the complex pathogenesis is challenging, thus, disease-modifying OA drugs are not available. Forkhead box O (FOXO) transcription factors contribute to cartilage homeostasis through autophagy and oxidative stress resistance. Here, we sought to discover FOXO activators and found that cyproheptadine, a histamine H1 receptor (HRH1) inverse agonist, promoted FOXO3 nuclear translocation and increased FOXO target genes while suppressing inflammation. In a murine OA model, cyproheptadine reduced structural joint tissue damage and pain behaviors. Mechanistically, the inhibition of HRH1 constitutive activity mediated the effects of cyproheptadine on calcium balance between endoplasmic reticulum (ER) and cytoplasm, and FOXO activation was part of this mechanism. The antiinflammatory effect of cyproheptadine involved the inhibition of protein kinase C/NF-κB pathway. HRH1 inhibition also suppressed osteogenesis in mesenchymal stem cells and nerve growth factor expression, which are mechanisms of osteophyte formation and pain behaviors. Moreover, cyproheptadine suppressed ER stress–induced lipogenesis by upregulating insulin-induced gene 1. Our findings suggest that HRH1 constitutive activity controls important OA-promoting mechanisms and indicate that HRH1 inverse agonists are promising drug repurposing candidates for structure and pain improvement in OA.

## Abstract 2

Constitutive activity of histamine H1 receptor is a novel therapeutic target for osteoarthritis and inverse agonists are promising candidates for drug repurposing.

## Graphical abstract

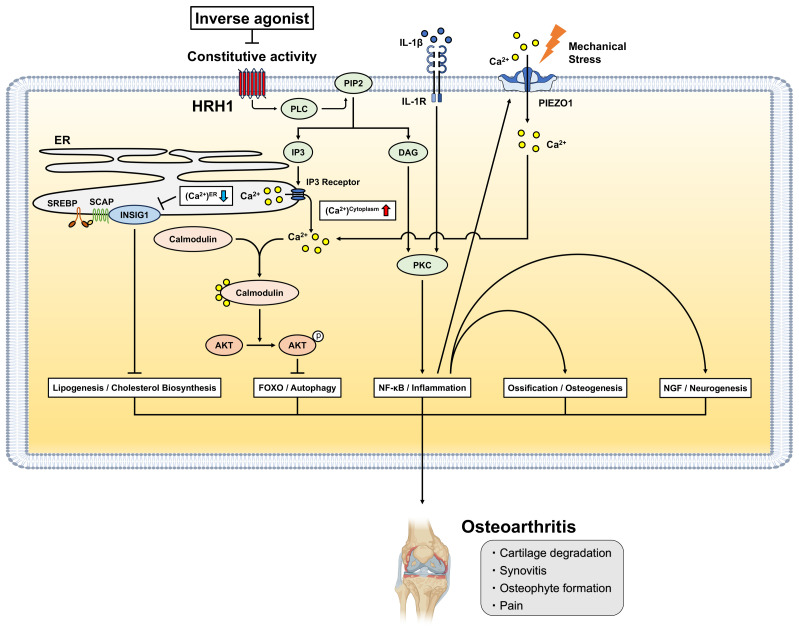

## Introduction

Osteoarthritis (OA) is the most prevalent joint disease, with joint pain and mobility limitations as the principal patient-reported outcomes (1). Despite large numbers of clinical trials, there are still no approved disease-modifying OA drugs (DMOADs) (2). A variety of factors are involved in the pathogenesis of OA, including disruption of cellular homeostasis, inflammation, abnormal ossification, and aberrant metabolism (3). Accordingly, OA patients exhibit diverse characteristic structural changes, including cartilage and meniscus degradation, synovitis, calcification of joint tissues, subchondral bone sclerosis, and osteophyte formation. The complexity of OA pathogenesis mechanisms poses a challenge to the selection of DMOAD candidates.

Aging is a major risk factor for OA (4, 5). Forkhead box O (FOXO) transcription factors play a central role in the regulation of autophagy and oxidative stress resistance and thereby reduce age-related diseases and promote longevity (6–9). We have previously found that FOXO1 and FOXO3 expression and activity are downregulated in OA cartilage and meniscus, and that *Foxo1-* and *Foxo3*-deficient mice spontaneously develop cartilage and meniscus degeneration (10–14). On the other hand, FOXO1 or FOXO3 overexpression enhances autophagy and antioxidant genes in chondrocytes (12, 13). Therefore, FOXO activation is a promising therapeutic strategy for OA.

The activity of FOXO is regulated by nuclear-cytoplasmic shuttling, in part via the phosphatidylinositol-3-OH kinase (PI3K)/AKT pathway (15). When phosphorylated by AKT, FOXO binds to exportin 1 (XPO1) and is translocated from the nucleus to the cytoplasm, where it is ubiquitinated and degraded (16). Drugs that increase nuclear FOXO in chondrocytes would thus be potential DMOADs.

In this study, we selected FOXO activators as candidates for testing in vitro in an OA animal model and for mechanistic analyses.

## Results

### Selection and prioritization of FOXO activators.

Based on the results of previous image-based drug screening of FOXO activators, we observed that most hit compounds fall into 2 major categories: XPO1 inhibitors and PI3K/AKT inhibitors (9). In addition, we considered the results of a recent in silico drug screen that matched FOXO3 overexpression and drug-response transcriptome data where hits were expected to have similar effects to FOXO3 overexpression (17). Among the top 130 hits, PI3K/AKT inhibitors were the major class, consistent with the results of image-based screening (Supplemental Table 1; supplemental material available online with this article; https://doi.org/10.1172/JCI183588DS1). Nine mammalian target of rapamycin (mTOR) inhibitors, which were also identified as FOXO activators in the image-based drug screening, were hits. Interestingly, 6 serotonin antagonists and 4 histamine antagonists that were not hits in the image-based screening were hits in the in-silico screening. From these previous studies, we selected 5 drugs for further evaluation (Supplemental Figure 1). This included selinexor, an XPO1 inhibitor; BEZ235, a dual PI3K/mTOR inhibitor; and cyproheptadine, an antagonist of serotonin and histamine receptors. In addition, we included LOM612 (18) and psammaplysene A (PSA) (19) from 2 image-based screens for agents that promote FOXO nuclear accumulation without affecting XPO1 or PI3K. These are the only agents positioned as FOXO-specific activators.

**Figure 1:**
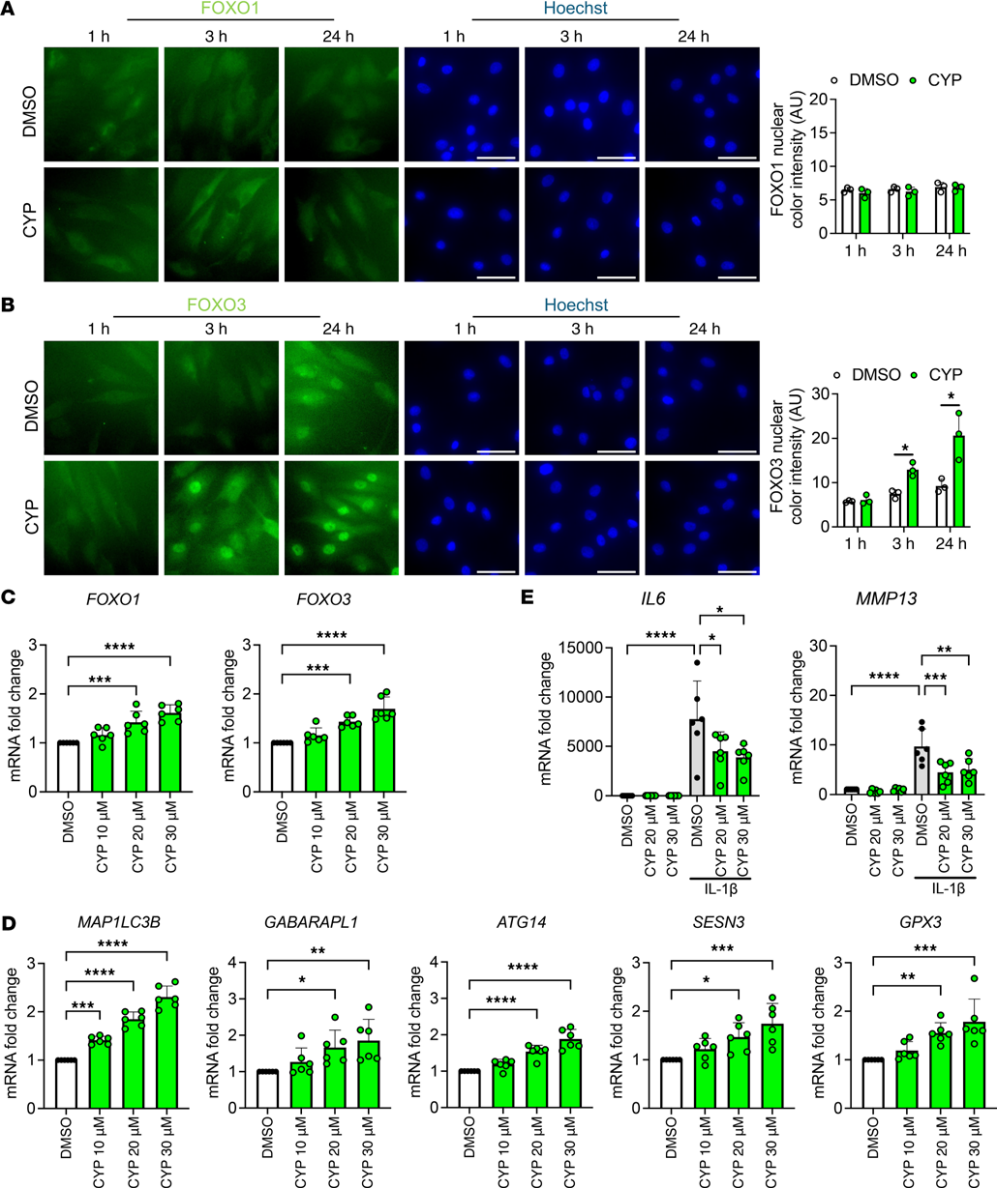
Effects of cyproheptadine in human chondrocytes. FOXO1 (**A**) and FOXO3 (**B**) localization in human chondrocytes (*n* = 3) 24 hours after treatment with DMSO or cyproheptadine (CYP) (30 μM) was analyzed by immunocytochemistry. Scale bar: 50 μm. Nuclear color intensity of FOXO1 and FOXO3 was quantified. Human chondrocytes (*n* = 6) were incubated with the indicated doses of CYP for 24 hours and RNA was isolated for qRT-PCR for *FOXO1* and *FOXO3* genes (**C**) and FOXO target genes (**D**). (**E**) Relative mRNA levels of *IL6* and *MMP13* in human chondrocytes (*n* = 6) incubated with IL-1β (1 ng/mL) for 6 hours after pretreatment with or without the indicated doses of CYP for 24 hours in qRT-PCR. Data are presented as means ± SD. Statistical analysis in **A** and **B** was performed using Student’s *t* test. Statistical analysis in **C** and **D** was performed using one-way ANOVA with the Dunnett’s post hoc test. Statistical analysis in **E** was performed using 1-way ANOVA with Tukey-Kramer post hoc test. *P < 0.05, **P < 0.01, ***P < 0.001, ****P < 0.0001.

To further characterize and prioritize these 5 drugs, human primary chondrocytes were treated for analysis of FOXO1 and FOXO3 nuclear localization at doses that did not significantly affect cell viability (Supplemental Figure 2). Selinexor increased nuclear levels of both FOXO1 and FOXO3 (Figure 1, A and B, and Supplemental Figure 3). BEZ235 increased nuclear levels of FOXO3 at 1 and 3 hours. Cyproheptadine increased nuclear FOXO3 after 3 hours. LOM612 and PSA had no effect on FOXO1 or FOXO3 localization and were excluded from further analyses.

Next, the expression of FOXO and FOXO target genes was analyzed by qRT-PCR after treatment of chondrocytes with the 3 drugs that increased nuclear FOXO. All 3 drugs increased *FOXO1* and *FOXO3* gene expression (Figure 1C and Supplemental Figure 4, A and B) and the expression of autophagy-related genes *MAP1LC3B*, *GABARAPL1*, and *ATG14* (Figure 1D and Supplemental Figure 4, C and D). The antioxidant *SESN3* was upregulated only by cyproheptadine, while BEZ235 downregulated it. *GPX3,* which also protects cells from oxidative damage, was increased by selinexor and cyproheptadine, but BEZ235 had little effect. These results indicated that selinexor and cyproheptadine induced FOXO and FOXO target genes more effectively than BEZ235. Furthermore, we tested the antiinflammatory effects of drugs against IL-1β, which is a potent inducer of OA-promoting factors, and found that cyproheptadine, but not selinexor or BEZ235, suppressed *IL6* and *MMP13* induction by IL-1β (Figure 1E and Supplemental Figure 4, E and F). Cyproheptadine also suppressed *IL6* and *MMP13* induction by IL-1β in human synoviocytes (Supplemental Figure 5).

To confirm whether the enhanced autophagy by cyproheptadine treatment was mediated by FOXO3, chondrocytes were transfected with FOXO3 siRNA, which suppressed the effect of cyproheptadine on the expression of autophagy-related genes and antioxidant genes (Supplemental Figure 6A). On the other hand, FOXO3 knockdown did not suppress the antiinflammatory effect of cyproheptadine (Supplemental Figure 6B).

Thus, cyproheptadine enhanced FOXO3 nuclear accumulation, promoted FOXO target gene expression, and had antiinflammatory effects independent of FOXO3, making it the most promising of the 5 drugs and was chosen for further characterization.

### Improvement of structural outcomes by cyproheptadine in an OA animal model.

**Figure 2:**
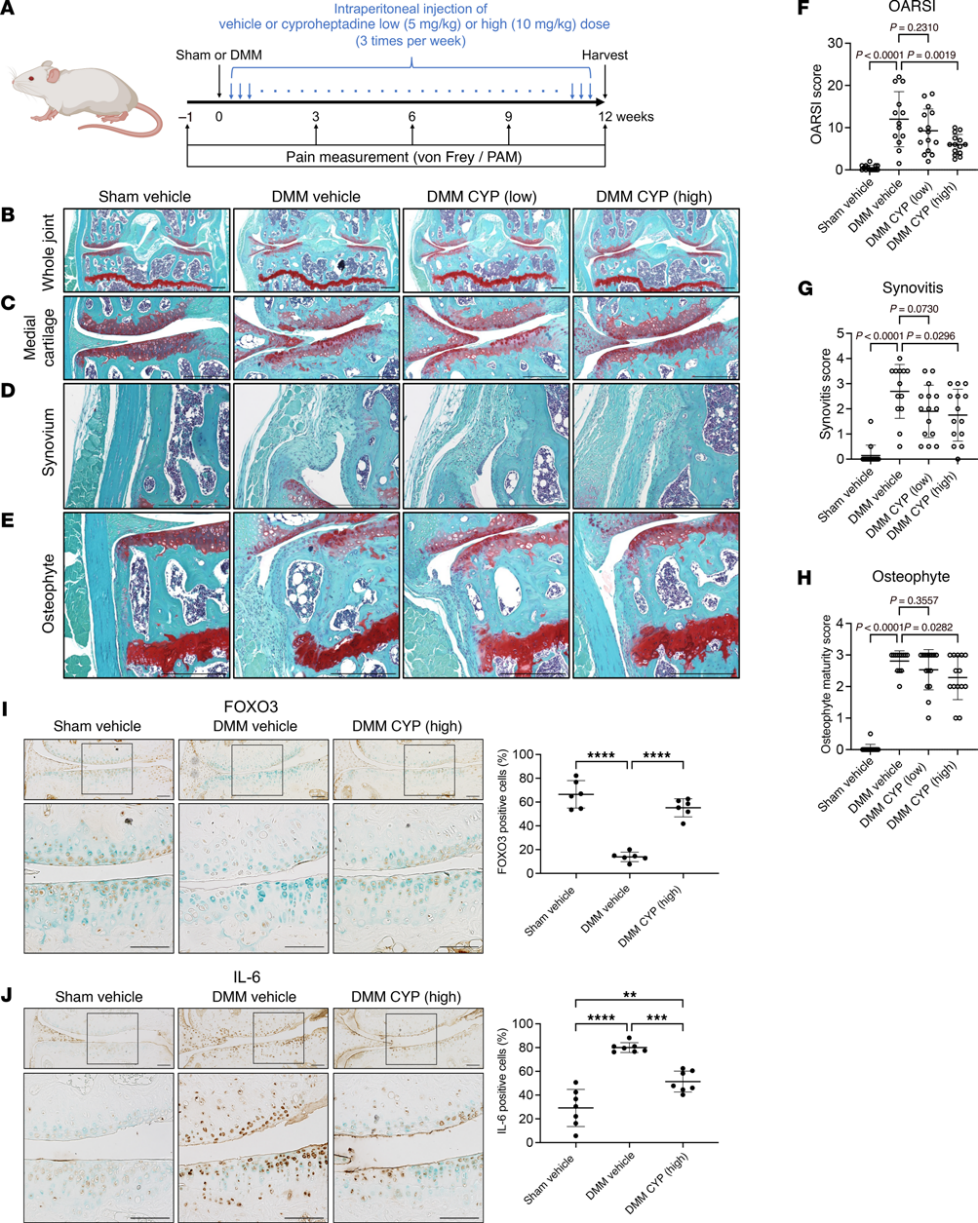
Effects of cyproheptadine on structural changes in mice with experimental OA. (**A**) Mice were treated with cyproheptadine (low dose: 5mg/kg, high dose: 10mg/kg) or control vehicle after DMM surgery. Representative Safranin-O staining images of whole joint (**B**), medial cartilage (**C**), synovium (**D**), and osteophyte (**E**). Scale bar: 300 μm. OARSI score of medial femoral condyle and tibial plateau (**F**), synovitis score (**G**), and osteophyte maturity score (**H**) following 12 weeks of cyproheptadine treatment. Sham vehicle (*n* = 14), DMM vehicle (*n* = 13), DMM CYP (low) (*n* = 15), and DMM CYP (high) (*n* = 14). Immunohistochemistry of FOXO3 (*n* = 6) (**I**) and IL-6 (*n* = 7) (**J**) in cartilage of mice with sham or DMM surgery treated with vehicle or CYP. Scale bar: 100 μm. Data are presented as means ± SD. Statistical analysis in **F**–**H** was performed using 1-way ANOVA with the Dunnett’s post hoc test. Statistical analysis in **I** and **J** was performed using 1-way ANOVA with the Tukey-Kramer post hoc test. **P < 0.01, ***P < 0.001, ****P < 0.0001.

We tested cyproheptadine in the OA animal model induced by surgical destabilization of the medial meniscus (DMM) in 15-week-old mice. Low (5 mg/kg) or high (10 mg/kg) doses of cyproheptadine were administered intraperitoneally 3 times per week starting 1 day after DMM surgery, and knee joints were harvested after 12 weeks (Figure 2A). Osteoarthritis Research Society International (OARSI) score showed that cartilage degradation was significantly attenuated in the cyproheptadine high-dose group compared with the control group (Figure 2, B, C, and F). Cyproheptadine high-dose treatment also suppressed synovitis and osteophyte formation (Figure 2, D, E, G, and H). IHC showed that FOXO3-positive cells were reduced in the cartilage of mice with DMM, but cyproheptadine enhanced the expression of FOXO3 (Figure 2I). Cyproheptadine also reduced the expression of IL-6 in cartilage of mice with DMM (Figure 2J).

### Transcriptomic changes in response to cyproheptadine.

**Figure 3:**
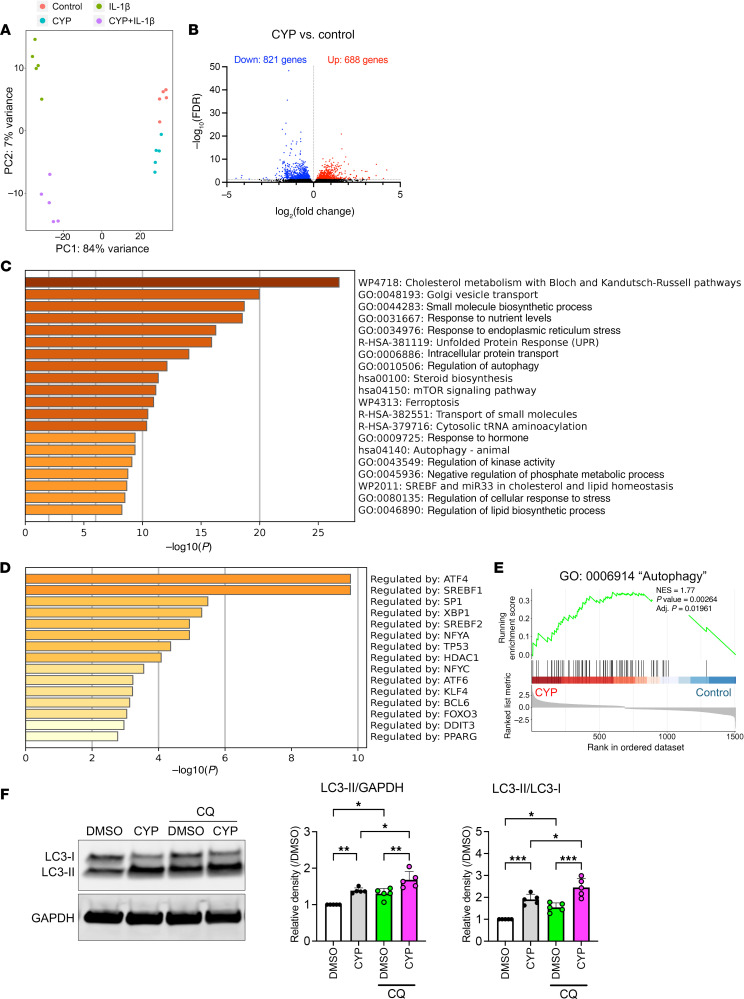
Transcriptomic changes induced by cyproheptadine in human chondrocytes. RNA-seq was performed on human chondrocytes (*n* = 5) treated with cyproheptadine (CYP) (30 μM) or control vehicle (DMSO) for 24 hours. For IL-1β stimulation, chondrocytes (n=5) were incubated with IL-1β (1 ng/mL) for 6 hours following the pretreatment with cyproheptadine (30 μM) for 24 hours. (**A**) Principal component analysis showing separation in 4 groups. (**B**) Volcano plot of the differentially expressed genes (DEGs) in CYP versus control. Metascape enrichment (**C**) and TRRUST (**D**) analysis using the upregulated genes after CYP treatment. (**E**) Gene set enrichment analysis (GSEA) showing enrichment of “autophagy” in chondrocytes treated with CYP. (**F**) Western blot analysis of LC3 in chondrocytes (*n* = 5) incubated with chloroquine (CQ) (25 μM) for 2 hours after pretreatment with or without CYP (30 μM) for 24 hours. Data are presented as means ± SD. Statistical analysis was performed using 1-way ANOVA with the Tukey-Kramer post hoc test. *P < 0.05, **P < 0.01, ***P < 0.001.

To determine candidate pathways that mediate cyproheptadine effects, RNA-seq was performed on chondrocytes treated with cyproheptadine with or without IL-1β stimulation. Principal component analysis could clearly distinguish 4 groups (Figure 3A). Cyproheptadine treatment increased the expression of 688 genes and decreased the expression of 821 genes (Figure 3B). Metascape enrichment analysis with the differentially expressed genes (DEGs) showed that “autophagy” was enriched in the upregulated DEGs (Figure 3C). Furthermore, the transcriptional regulatory relationships unraveled by sentence-based text-mining (TRRUST) analysis (20) showed that FOXO3 target genes were significantly increased after cyproheptadine treatment (Figure 3D). In the downregulated DEGs, “cell cycle” was enriched (Supplemental Figure 7, A and B). In gene set enrichment analysis (GSEA), we confirmed that autophagy was induced in cyproheptadine-treated chondrocytes (Figure 3E) and this included 42 autophagy-related genes (Supplemental Figure 7C). To further confirm the effect of cyproheptadine on autophagy flux, Western blotting was performed. Cyproheptadine induced the expression of LC3-II, an active form of LC3 protein, and the LC3-II/LC3-I ratio (Figure 3F). Similar changes were also observed in the presence of chloroquine, a lysosome inhibitor. These results indicate that cyproheptadine induces not only the expression of autophagy-related genes but also autophagy flux.

Notably, metascape enrichment analysis with the upregulated DEGs revealed that cyproheptadine also modulated several events in the ER, including “cholesterol/lipid metabolism”, “golgi vesicle transport”, “response to ER stress”, “unfolded protein response (UPR)”, “intracellular protein transport”, and “steroid biosynthesis” (21–23) (Figure 3C). TRRUST analysis also showed that cyproheptadine upregulated activating transcription factor 4 (ATF4), ATF6, x-box binding protein 1 (XBP1),and DNA damage inducible transcript 3 (DDIT3), which regulate UPR, and sterol regulatory element binding transcription factor 1 (SREBF1) and SREBF2, which are the master regulators of lipid/cholesterol homeostasis (Figure 3D). These transcription factors are closely related to ER function (21, 22). GSEA showed similar enrichment of pathways related to ER (Supplemental Figure 7, D–I). This suggests a key role of the ER in signaling induced by cyproheptadine.

**Figure 4:**
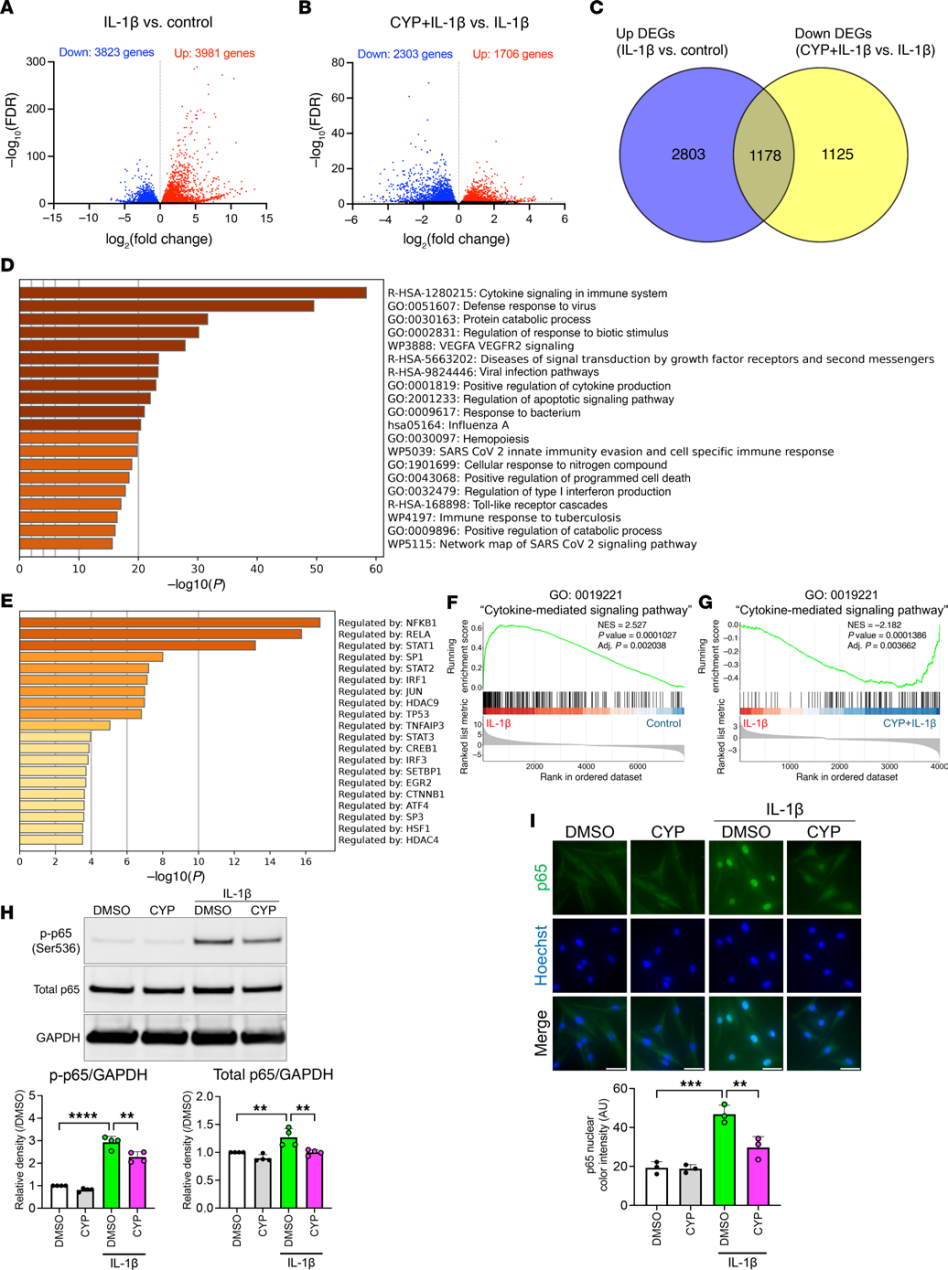
Antiinflammatory effects of cyproheptadine via NF-κB pathway. (**A**) Volcano plot of the DEGs in RNA-seq performed on human chondrocytes (*n* = 5) treated with IL-1β (1 ng/mL) for 6 hours. (**B**) Volcano plot of the DEGs in RNA-seq performed on human chondrocytes (*n* = 5) treated with IL-1β (1 ng/mL) for 6 hours after pretreatment with or without cyproheptadine (CYP) for 24 hours. (**C**) Venn diagram of the shared upregulated genes by IL-1β stimulation and the downregulated genes by CYP treatment under IL-1β stimulation. Metascape enrichment (**D**) and TRRUST (**E**) analysis using the shared genes in **C**. (**F**) GSEA showing induced “cytokine-mediated signaling pathway” in chondrocytes treated with IL-1β. (**G**) GSEA showing inhibited “cytokine-mediated signaling pathway” in chondrocytes treated with CYP under IL-1β stimulation. (**H**) Western blot analysis of total p65 and phosphorylated p65 at Ser536 (p-p65 (Ser536)) in chondrocytes (*n* = 4) incubated with IL-1β (1 ng/mL) for 20 minutes after pretreatment with or without CYP (30 μM) for 24 hours. (**I**) Immunocytochemistry of p65 in chondrocytes (*n* = 3) incubated with IL-1β (1 ng/mL) for 20 minutes after pretreatment with or without CYP (30 μM) for 24 hours. Scale bar: 50 μm. Data are presented as means ± SD. Statistical analysis was performed using 1-way ANOVA with the Tukey-Kramer post hoc test. **P < 0.01, ***P < 0.001, ****P < 0.0001.

We also performed RNA-seq of chondrocytes under IL-1β stimulation, which changed the expression of large numbers of genes (3,981 up and 3,823 down) (Figure 4A). Metascape enrichment analysis showed that “cytokine signaling” was strongly upregulated by IL-1β stimulation, and TRRUST analysis showed that NF-κB target genes were strongly upregulated (Supplemental Figure 8, A and B). In the downregulated DEGs, “cell cycle” was enriched (Supplemental Figure 8, C and D). Under IL-1β stimulation and cyproheptadine treatment, 1,706 genes were upregulated, and 2,303 genes were downregulated (Figure 4B). Enrichment analysis using the downregulated DEGs revealed that cyproheptadine treatment suppressed cytokine signaling, and TRRUST analysis showed that NF-κB target genes were suppressed by cyproheptadine treatment (Supplemental Figure 9, A and B). We further determined which IL-1β–induced genes were suppressed by cyproheptadine and found that approximately one-third of the genes induced by IL-1β were suppressed by cyproheptadine (Figure 4C). These shared genes were still strongly enriched in cytokine signaling and NF-κB pathway (Figure 4, D and E). In GSEA, “cytokine-mediated signaling pathway” was also upregulated after IL-1β treatment (Figure 4F). Cyproheptadine treatment suppressed this pathway under IL-1β treatment (Figure 4G) and inhibited 75 genes in this annotation induced by IL-1β treatment (Supplemental Figure 9C). To further confirm the effect of cyproheptadine on NF-κB pathway, we performed Western blotting and found that cyproheptadine suppressed p65 phosphorylation induced by IL-1β (Figure 4H). Consistently, immunocytochemistry showed that cyproheptadine suppressed the nuclear translocation of p65 induced by IL-1β (Figure 4I). These results indicate that cyproheptadine suppressed cytokine signaling at least in part by inhibiting NF-κB pathway.

Cyproheptadine also induced “cellular response to external stimulus” and the target genes of PPAR-γ, a known NF-κB suppressor, under IL-1β stimulation (Supplemental Figure 10, A and B). We found that the suppression of 944 genes by IL-1β was restored by cyproheptadine, and these genes were also enriched in PPAR-γ target genes (Supplemental Figure 10, C–E).

### Receptors mediating cyproheptadine effects.

**Figure 5:**
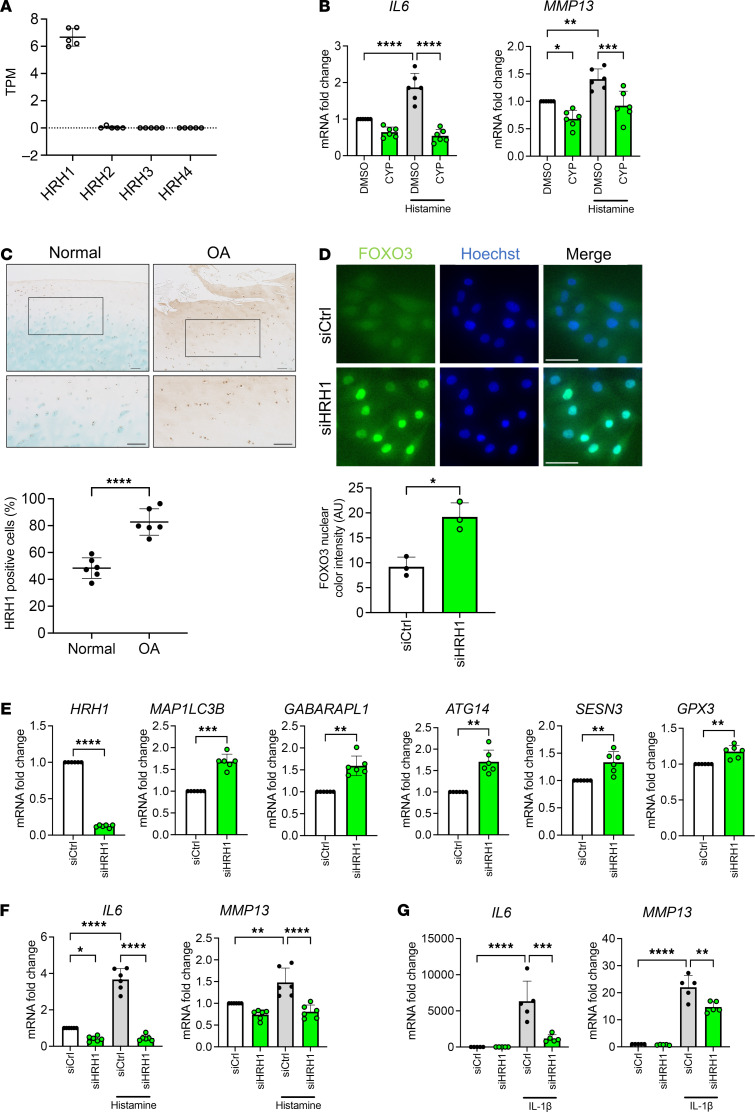
Cyproheptadine signaling via histamine H1 receptor. (**A**) Transcripts per kilobase million (TPM) in RNA-seq of human chondrocytes (*n* = 5) for histamine receptors. (**B**) Relative mRNA levels of *IL6* and *MMP13* in human chondrocytes (*n* = 6) incubated with histamine (10 μM) for 6 hours after pretreatment with cyproheptadine (CYP) (30 μM) for 24 hours. (**C**) IHC of HRH1 in human normal and OA cartilage. *n* = 6. Scale bar: 100 μm. (**D**) Immunocytochemistry of FOXO3 in human chondrocytes (*n* = 3) transfected with siCtrl or siHRH1. Scale bar: 50 μm. (**E**) Relative mRNA levels of *HRH1* and FOXO target genes in chondrocytes (*n* = 6) transfected with siCtrl or siHRH1. Human chondrocytes were incubated with histamine (10 μM) (*n* = 6) (**F**) or IL-1β (1 ng/mL) (*n* = 5) (**G**) for 6 hours after siRNA transfection, and RNA was isolated for qRT-PCR for *IL6* and *MMP13* genes. Data are presented as means ± SD. Statistical analysis in **B**, **F**, and **G** was performed using 1-way ANOVA with the Tukey-Kramer post hoc test. Statistical analysis in **C**–**E** was performed using Student’s *t* test. *P < 0.05, **P < 0.01, ***P < 0.001, ****P < 0.0001.

Cyproheptadine is known to bind several types of receptors, including histamine, serotonin, and muscarinic acetylcholine receptors, which are G protein–coupled receptors (GPCRs) that trigger calcium release from the ER to the cytoplasm via phospholipase C (PLC)/inositol trisphosphate (IP3) axis (24). Because cyproheptadine affected many events in the ER, as seen in RNA-seq, we wanted to determine which specific receptors are involved. We first checked their gene expression by analyzing our RNA-seq data of human primary chondrocytes. Histamine H1 receptor (HRH1) was the most highly expressed receptor (Figure 5A). mRNA levels for all other receptors had very low read counts (< 0.5 TPM) (Figure 5A and Supplemental Figure 11, A and B). This profile suggests that HRH1 inhibition mediates the effects of cyproheptadine. We also found that chondrocytes expressed only a few enzymes required for the synthesis of the ligands of these receptors, suggesting that chondrocytes are not a relevant source of these ligands (Supplemental Figure 11C).

In chondrocytes, histamine induced a small but significant increase in the expression of *IL6* and *MMP13* and this was canceled by cyproheptadine pretreatment (Figure 5B). In synoviocytes, histamine slightly induced *IL6* and *MMP13*, and this was abolished by cyproheptadine (Supplemental Figure 11D). To determine the relationship between HRH1 and its antagonists in chondrocytes, we tested desloratadine, a second-generation histamine H1 antagonist. Cell viability testing showed toxicity of desloratadine at lower doses compared with cyproheptadine (Supplemental Figure 12A) and it was thus used at lower doses than cyproheptadine in the subsequent experiments. Desloratadine treatment of chondrocytes promoted FOXO3 but not FOXO1 nuclear translocation (Supplemental Figure 12, B and C) and increased the expression of FOXO target genes (Supplemental Figure 12D). Desloratadine also suppressed the induction of *IL6* and *MMP13* under IL-1β stimulation (Supplemental Figure 12E). These results support the notion that signaling via HRH1 mediates the effects of these HRH1 antagonists.

Some GPCRs, including histamine receptors, are known to have constitutive activity, which is observed in the absence of agonists and may be enhanced by increased expression of these receptors (25). We next analyzed the expression of HRH1 by IHC and found that it was increased in OA cartilage compared with normal cartilage (Figure 5C).

Most of the histamine antagonists in clinical use, including cyproheptadine and desloratadine, are known to exhibit inverse agonism and suppress the constitutive activity of their receptors (25). Because our previous results showed cyproheptadine effects in the absence of histamine, we hypothesized that cyproheptadine functions in chondrocytes as an inverse agonist. To directly test the role of the constitutive activity of HRH1 in chondrocytes, we knocked down HRH1 with siRNA. This resulted in similar effects as treatment with cyproheptadine, including nuclear accumulation of FOXO3 (Figure 5D) and increased expression of autophagy-related and antioxidant genes (Figure 5E). In chondrocytes, HRH1 knockdown suppressed the induction of *IL6* and *MMP13* in the presence of histamine (Figure 5F). Importantly, HRH1 knockdown also suppressed IL-1β–induced *IL6* and *MMP13* (Figure 5G)*,* suggesting interactions between signaling through the HRH1 and the IL-1 pathway. Similarly, in synoviocytes, HRH1 knockdown suppressed the induction of *IL6* and *MMP13* by histamine and IL-1β treatment, respectively (Supplemental Figure 13).

Collectively, these findings suggest that, in chondrocytes and synoviocytes, there is constitutive, ligand-independent HRH1 activation, which regulates FOXO3 nuclear localization and the expression of OA-relevant genes.

### Cyproheptadine effects calcium signaling by modulating calcium balance in ER and cytoplasm, calcium binding proteins, and mechanosensitive ion channels.

**Figure 6:**
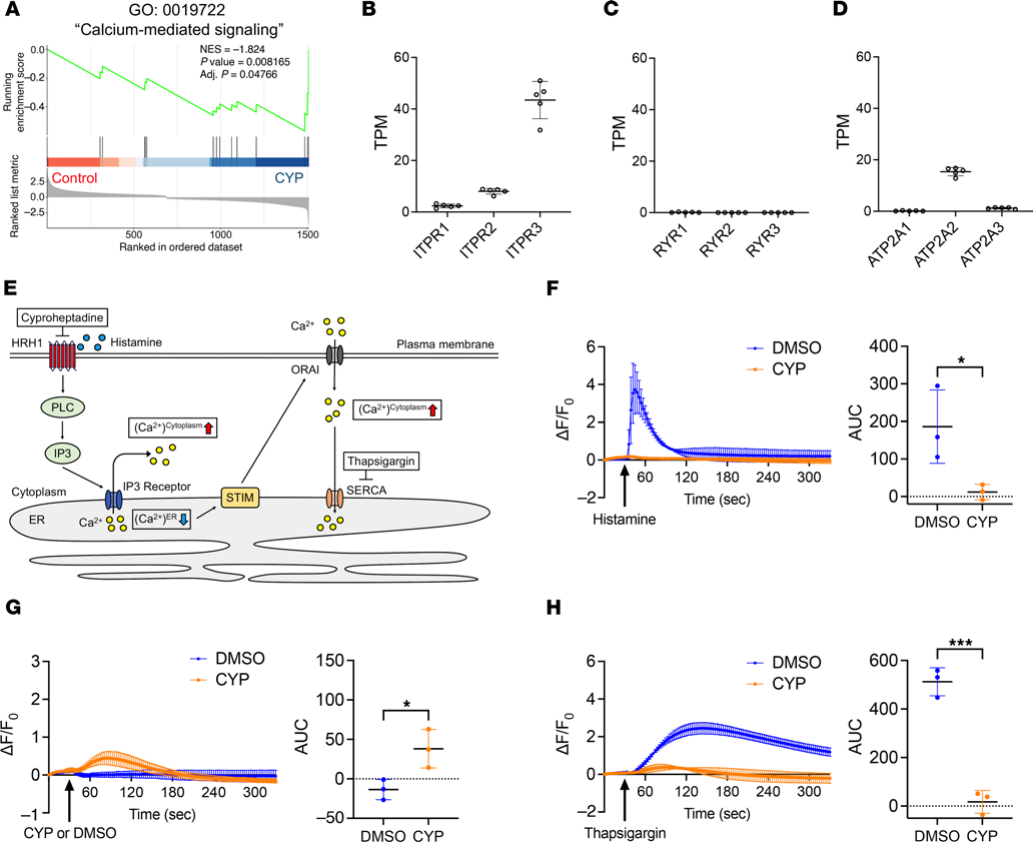
Cyproheptadine regulation of calcium signaling. (**A**) GSEA showing inhibited “calcium-mediated signaling” in chondrocytes treated with cyproheptadine (CYP). TPM in RNA-seq of human chondrocytes (*n* = 5) for ITPRs (**B**), RYRs (**C**), and ATP2As (**D**). (**E**) Overview of compound effects on intracellular calcium dynamics via HRH1 signaling. (**F**) Intracellular calcium levels in TC28 cells (*n* = 3) following histamine (10 μM) stimulation after pretreatment with DMSO or cyproheptadine (CYP) (30 μM) for 1 hour. (**G**) Intracellular calcium levels in TC28 cells (*n* = 3) with DMSO or CYP treatment. (**H**) Intracellular calcium levels in TC28 cells (*n* = 3) following thapsigargin (1 μM) stimulation after pretreatment with DMSO or CYP for 1 hour. Data are presented as means ± SD. Statistical analysis was performed using Student’s *t* test. *P < 0.05, ***P < 0.001.

HRH1 signaling triggers calcium release from the ER into the cytoplasm where it binds to calmodulin and exhibits various functions as a second messenger (26). We recognized that, although many of the hit compounds were classified as XPO1 inhibitors or PI3K inhibitors in previous image-based drug screening for FOXO activators (9), calmodulin inhibitors or calcium chelators have also been identified as FOXO nuclear translocators in 2 separate drug screenings (27, 28). Indeed, GSEA showed that calcium-mediated signaling was suppressed by cyproheptadine treatment (Figure 6A). Therefore, we hypothesized that calcium signaling is a key mediator of the effects of cyproheptadine in chondrocytes.

We first determined the expression of molecules that mediate calcium transport between ER and cytoplasm by analyzing RNA-seq data of human primary chondrocytes. IP3 receptors release calcium from the ER. All types of ITPR genes that encode IP3 receptors were expressed in chondrocytes, and *ITPR3* was the most highly expressed (Figure 6B). Ryanodine receptors also release calcium from the ER and are generally expressed in excitable cells, such as muscle cells and neurons (29). In chondrocytes, RYR genes were not expressed (Figure 6C). The sarco/endoplasmic reticulum Ca^2+^-ATPase (SERCA), encoded by ATP2As, is responsible for transporting calcium ions from the cytoplasm into the ER lumen (30). Only *ATP2A2* was expressed in chondrocytes (Figure 6D). This indicates that ER calcium homeostasis in chondrocytes is regulated by the balance between IP3 receptors and SERCA (Figure 6E).

When histamine binds to HRH1, PLC is activated and IP3 is synthesized, which binds to the IP3 receptor, which releases calcium from the ER to the cytoplasm (25) (Figure 6E). After histamine promotes calcium release from the ER, stromal interaction molecule (STIM) is activated in response to decreased calcium levels in the ER, which subsequently activates ORAI, a calcium channel on the plasma membrane, to allow extracellular calcium influx and restore calcium in the ER through SERCA (30). Indeed, histamine induced a rapid increase in intracellular calcium levels in TC28 chondrocytes (Figure 6F and Supplemental Video 1), and this histamine effect was blocked by cyproheptadine pretreatment (Figure 6F and Supplemental Video 2). This indicates that cyproheptadine blocks the binding of histamine to HRH1 and inhibits calcium release from the ER. We next tested the effect of cyproheptadine alone on intracellular calcium flux. Cyproheptadine treatment significantly increased total calcium levels, indicating cyproheptadine action as an inverse agonist on intracellular calcium balance (Figure 6G and Supplemental Videos 3 and 4). To ensure that the effect of cyproheptadine on calcium balance is mediated by the maintenance of calcium levels in the ER, we treated TC28 chondrocytes with thapsigargin, a SERCA inhibitor that blocks calcium uptake from the cytoplasm to the ER. As thapsigargin treatment decreases calcium concentration in the ER, extracellular calcium influx via the STIM/ORAI axis occurs (Figure 6E). We found that cyproheptadine inhibited calcium influx induced by thapsigargin treatment (Figure 6H and Supplemental Videos 5 and 6). These results indicate that cyproheptadine can maintain calcium concentration in the ER by not only blocking histamine stimulation but also by inhibiting the constitutive activity of HRH1, which promotes calcium release from the ER. The overall result of these effects is that cyproheptadine suppresses calcium signaling in the cytoplasm.

Calcium-binding proteins play a pivotal role in cellular calcium signaling by binding free calcium in the cytoplasm. Our RNA-seq data indicated that the gene ontology of “calcium ion binding” was enriched in OA compared with healthy human cartilage, with increased expression of *CALM1*, *CALM2*, and *CALM3*, encoding calmodulins, the major calcium-binding proteins (31) (Supplemental Figure 14A). Because calmodulin inhibitors had been identified as FOXO activators (27, 28), we explored possible effects of cyproheptadine on the expression of calcium-binding proteins. First, we checked RNA-seq data for the overlapping genes between the gene ontology of “calcium ion binding” and the downregulated genes in chondrocytes treated with cyproheptadine. We found 26 shared genes, including *CALM3* (Supplemental Figure 14B). In RNA-seq data, the expression of *CALM3* was the highest among 3 CALM genes in human OA chondrocytes (Supplemental Figure 14C). qRT-PCR showed that cyproheptadine decreased *CALM1*, *CALM2* and *CALM3* (Supplemental Figure 14D). HRH1 knockdown also suppressed *CALM1*, *CALM2* and *CALM3* (Supplemental Figure 14E). These results indicate that HRH1 inhibition suppressed not only calcium release from the ER but also the expression of calmodulin which was upregulated in OA cartilage.

Cytoplasmic calcium concentrations are maintained at approximately 10,000-fold lower levels than extracellular and intra-ER calcium concentrations, and calcium influx into the cytoplasm due to various stimuli is an important signaling mechanism (26). Our previous results demonstrated the inhibitory effect of cyproheptadine on the increase in intracellular calcium following calcium release from the ER into the cytoplasm, while extracellular calcium influx is another major source of calcium in the cytoplasm. Recent reports showed that increased mechanical stress causes excessive intracellular calcium influx via mechanosensitive ion channels, and enhanced calcium signaling leads to inflammation and chondrocyte apoptosis (32). Thus, we explored the effect of cyproheptadine on extracellular calcium influx. Analysis of RNA-seq data confirmed that chondrocytes express the mechanosensitive channels *PIEZO1*, *PIEZO2*, *TRPV2*, and *TRPV4* (Supplemental Figure 15A). On the other hand, only very low levels of CACNAs, which encode voltage-dependent calcium channels, were detected in chondrocytes. Interestingly, our RNA-seq results further showed that cyproheptadine downregulated *PIEZO1* gene expression (Supplemental Figure 15B). In addition, *PIEZO1* and *TRPV4* were increased by IL-1β stimulation, and cyproheptadine suppressed IL-1β-induced *PIEZO1* expression. Under IL-1β stimulation, *PIEZO2* and *TRPV2* were suppressed by cyproheptadine. qRT-PCR showed that cyproheptadine suppressed *PIEZO1*, *PIEZO2,* and *TRPV2* expression with or without IL-1β stimulation (Supplemental Figure 15C). We then tested the effect of HRH1 knockdown by siRNA (Supplemental Figure 15D). HRH1 knockdown also decreased *PIEZO1* and *TRPV2* expression with or without IL-1β stimulation, and *PIEZO2* in the absence of IL-1β. Under IL-1β stimulation, HRH1 knockdown suppressed *TRPV4* although cyproheptadine did not suppress it. These results suggest that HRH1 inhibition prevents the inflammation-induced expression of mechanosensitive channels in chondrocytes.

### Mechanisms of cyproheptadine effects via calcium signaling and protein kinase C pathway.

**Figure 7:**
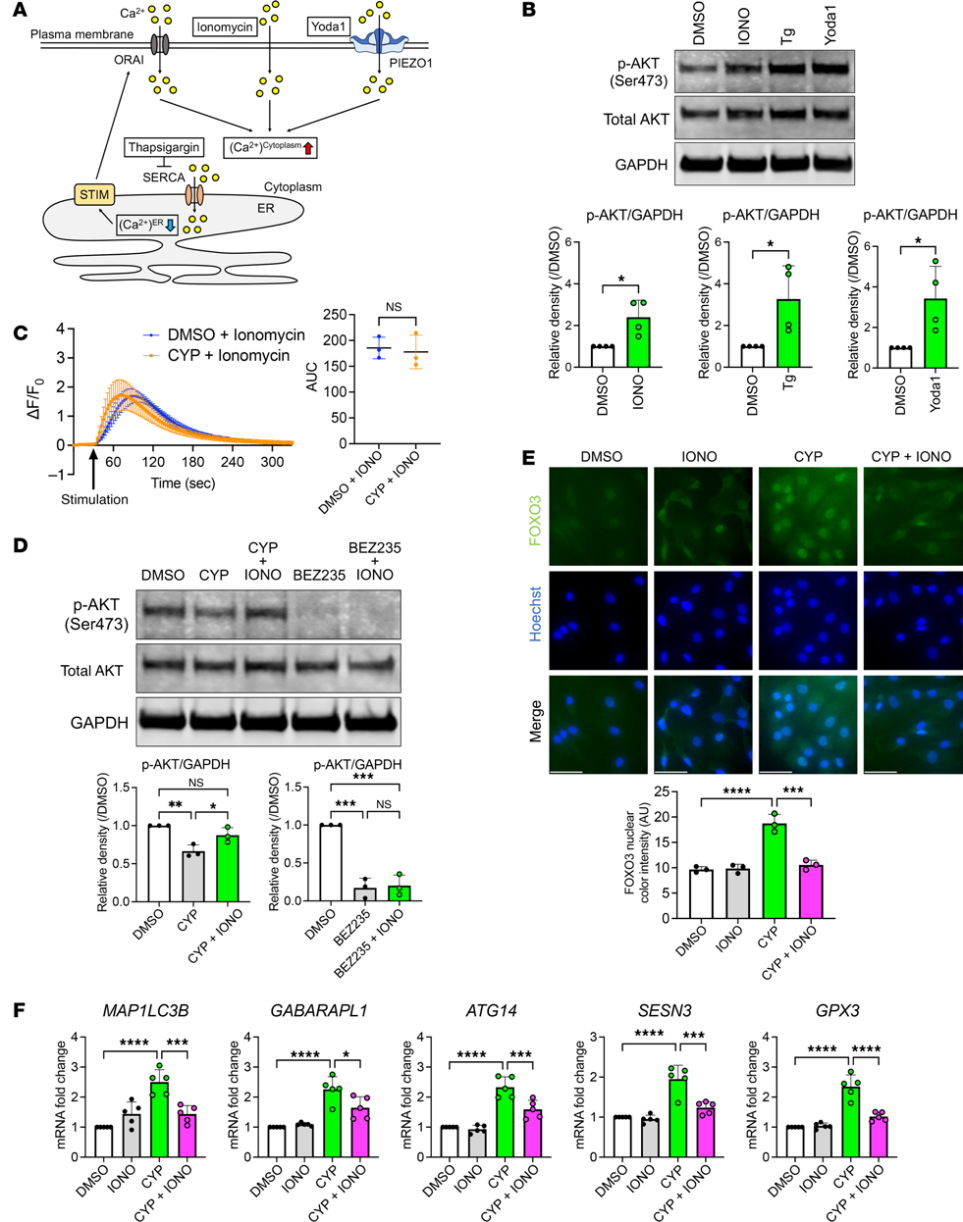
Mechanisms of cyproheptadine effects on the FOXO/autophagy axis via calcium signaling. (**A**) Overview of compound effects on intracellular calcium dynamics. (**B**) Western blot analysis of total AKT and phosphorylated AKT at Ser473 (p-AKT (Ser473)) in chondrocytes (*n* = 4) incubated with ionomycin (IONO) (0.2 μM), thapsigargin (Tg) (1 μM), or Yoda1 (10 μM) for 30 minutes. (**C**) Intracellular calcium levels in TC28 cells (*n* = 3) following IONO (0.2 μM) stimulation with DMSO or CYP (30 μM). (**D**) Western blot analysis of total AKT and p-AKT (Ser473) in human chondrocytes (*n* = 3) incubated with DMSO, CYP (30 μM), CYP and IONO (0.2 μM), BEZ235 (0.1 μM), or BEZ235 and IONO for 30 minutes. (**E**) Immunocytochemistry of FOXO3 in human chondrocytes (*n* = 3) 24 hours after treatment with CYP and IONO. Scale bar: 50 μm. (**F**) Relative mRNA levels of FOXO target genes in chondrocytes (*n* = 5) treated with CYP and IONO. Data are presented as means ± SD. Statistical analysis in **B** and **C** was performed using Student’s *t* test. Statistical analysis in **D**–**F** was performed using 1-way ANOVA with the Tukey-Kramer post hoc test. *P < 0.05, **P < 0.01, ***P < 0.001, ****P < 0.0001.

We next explored mechanisms of the cyproheptadine effects on FOXO3 activation. Because cyproheptadine and BEZ235, a dual PI3K/mTOR inhibitor induced the nuclear accumulation of only FOXO3 while the XPO1 inhibitor selinexor induced the nuclear accumulation both of FOXO1 and FOXO3 (Figure 1, A and B, and Supplemental Figure 3), we hypothesized that cyproheptadine inhibited PI3K/AKT pathway but not XPO1. Indeed, previous studies reported that calcium signaling induced AKT signaling (33). Therefore, we examined whether the effects of cyproheptadine in chondrocytes are mediated by calcium signaling in the cytoplasm and by AKT signaling. Ionomycin is a calcium ionophore, which can facilitate the transport of calcium across the plasma membrane and increase calcium levels in the cytoplasm (Figure 7A). In addition to ionomycin, we used thapsigargin and Yoda1, an activator of PIEZO1, to increase calcium levels in the cytoplasm. We first showed that all 3 reagents could induce AKT phosphorylation (Figure 7B), which indicates that calcium signaling in the cytoplasm activates AKT in chondrocytes. We next tested whether ionomycin suppressed the effects of cyproheptadine. We confirmed that ionomycin increased intracellular calcium concentration and that cyproheptadine administered concurrently with ionomycin did not suppress the calcium increase (Figure 7C). Cyproheptadine suppressed the phosphorylation of AKT, but ionomycin cancelled it, although ionomycin could not inhibit the effect of BEZ235, a direct PI3K inhibitor (Figure 7D). These results suggest that cyproheptadine suppresses AKT phosphorylation by inhibiting calcium signaling in the cytoplasm and induces FOXO3 activation. Consistently, ionomycin suppressed FOXO3 nuclear accumulation induced by cyproheptadine (Figure 7E) and the upregulation of FOXO target genes by cyproheptadine (Figure 7F).

**Figure 8:**
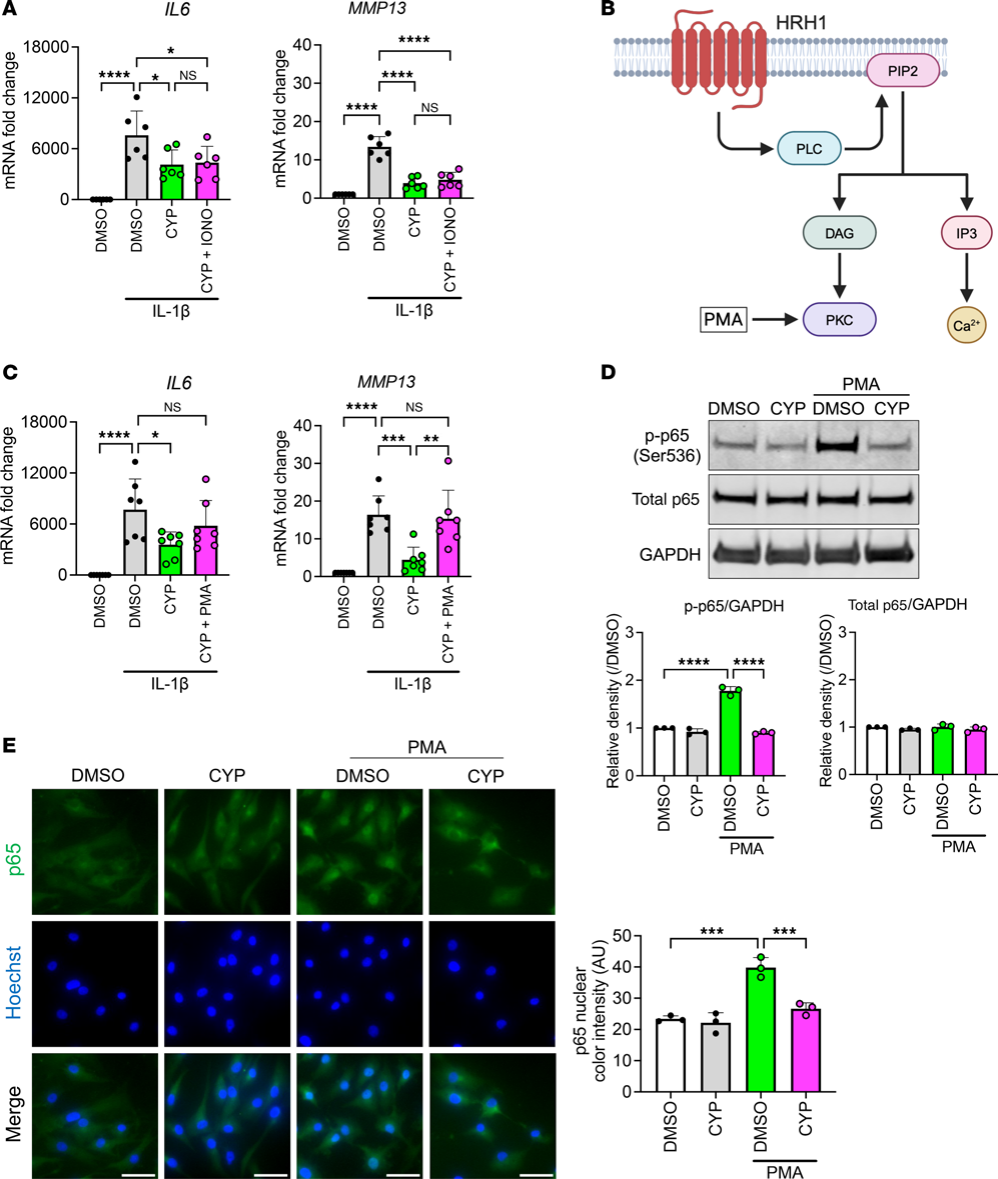
Mechanisms of cyproheptadine antiinflammatory effects via protein kinase C pathway. (**A**) Relative mRNA levels of *IL6* and *MMP13* in human chondrocytes (*n* = 6) incubated with IL-1β (1 ng/mL) for 6 hours after pretreatment with or without cyproheptadine (CYP) and ionomycin (IONO). (**B**) Diagram of HRH1 signaling pathway. (**C**) Relative mRNA levels of *IL6* and *MMP13* in human chondrocytes (*n* = 7) incubated with IL-1β (1 ng/mL) for 6 hours after pretreatment with or without CYP and phorbol 12-myristate 13-acetate (PMA) (20 nM). (**D**) Western blot analysis of total p65 and phosphorylated p65 at Ser536 (p-p65 (Ser536)) in chondrocytes (*n* = 3) incubated with PMA (20 nM) for 20 minutes after pretreatment with or without CYP (30 μM) for 24 hours. (**E**) Immunocytochemistry of p65 in chondrocytes (*n* = 3) incubated with PMA (20 nM) for 40 minutes after pretreatment with or without CYP (30 μM) for 24 hours. Scale bar: 50 μm. Data are presented as means ± SD. Statistical analysis was performed using 1-way ANOVA with the Tukey-Kramer post hoc test. *P < 0.05, **P < 0.01, ***P < 0.001, ****P < 0.0001.

On the other hand, ionomycin did not suppress the antiinflammatory effects of cyproheptadine (Figure 8A). Protein kinase C (PKC) is also part of HRH1 signaling and a NF-κB activator in T lymphocytes (Figure 8B) (34). Phorbol 12-myristate 13-acetate (PMA), a PKC activator, cancelled the antiinflammatory effects of cyproheptadine under IL-1β stimulation in chondrocytes (Figure 8C). In addition, we found that cyproheptadine inhibited the phosphorylation and nuclear translocation of p65 induced by PMA treatment (Figure 8, D and E), indicating that the antiinflammatory effects of cyproheptadine are mediated by the inhibition of the PKC/NF-κB pathway.

### Cyproheptadine effects on ossification and osteogenesis.

Based on our observation that cyproheptadine administration significantly suppressed osteophyte formation in the mouse OA model, we explored the mechanism by which cyproheptadine affects osteophyte formation. RNA-seq results showed that GO: 0001503 “ossification” was enriched in upregulated DEGs in IL-1β-stimulated chondrocytes (Supplemental Figure 16A). Furthermore, cyproheptadine suppressed the expression of genes involved in ossification under IL-1β stimulation. We determined which ossification-related genes were upregulated by IL-1β and which were downregulated by cyproheptadine and identified 56 genes (Supplemental Figure 16B). These genes included *YAP1*, *IGF1*, *LEF1*, *PTGS2*, *PTGER4*, and *BMP2*, reported to be involved in osteophyte formation. qRT-PCR analysis validated that the genes were suppressed both by cyproheptadine treatment and HRH1 knockdown under IL-1β stimulation, and only *IGF1* was not suppressed by HRH1 knockdown (Supplemental Figure 16, C and D). These results suggest that HRH1 inhibition suppresses ossification-related genes induced by inflammation stimulus in chondrocytes, which may be a mechanism that mediates the inhibition of osteophyte formation as observed in the mouse OA model.

**Figure 9:**
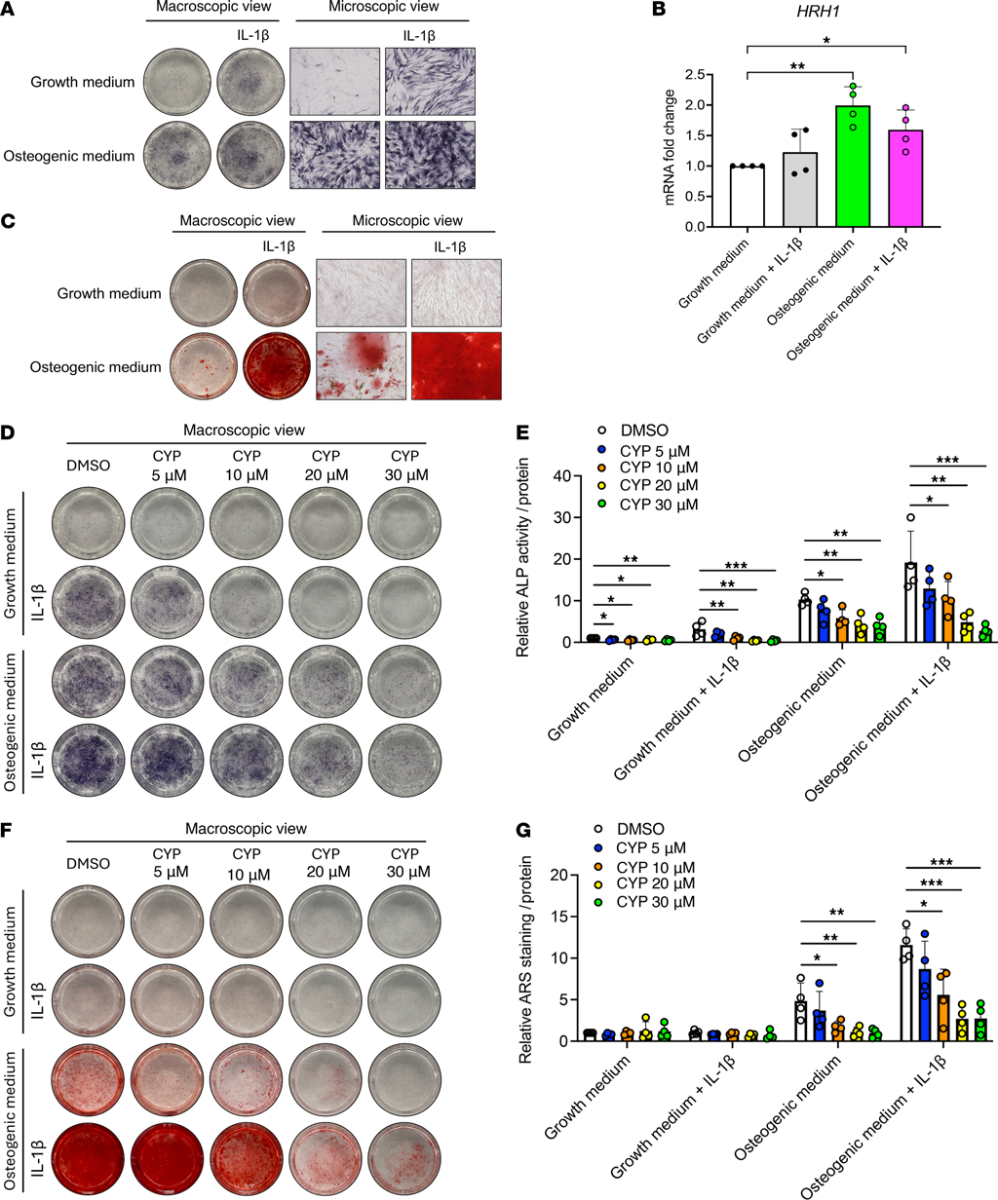
Effects of cyproheptadine on osteogenesis. (**A**) Alkaline phosphatase (ALP) staining in human mesenchymal stem cells (MSCs) incubated in growth medium or osteogenic medium in the presence or absence of IL-1β (1 ng/mL) for 7 days. (**B**) Relative mRNA levels of *HRH1* in MSCs (*n* = 4) incubated in growth medium or osteogenic medium in the presence or absence of IL-1β (1 ng/mL) for 7 days. (**C**) Alizarin red S staining in MSCs incubated in growth medium or osteogenic medium in the presence or absence of IL-1β (1 ng/mL) for 28 days. ALP staining (**D**) and relative ALP activity (**E**) in MSCs (*n* = 4) incubated in growth medium or osteogenic medium in the presence or absence of CYP (5, 10, 20, or 30 μM) and IL-1β (1 ng/mL) for 7 days. Alizarin red S (ARS) staining (**F**) and its relative quantification (**G**) in MSCs (*n* = 4) incubated in growth medium or osteogenic medium in the presence or absence of CYP (5, 10, 20 or 30 μM) and IL-1β (1 ng/mL) for 28 days. Data are presented as means ± SD. Statistical analysis was performed using 1-way ANOVA with the Dunnett’s post hoc test. *P < 0.05, **P < 0.01, ***P < 0.001.

Furthermore, we confirmed the effects of cyproheptadine on osteogenesis in mesenchymal stem cells (MSCs). We found that IL-1β treatment accelerated osteogenic differentiation of MSCs as measured by alkaline phosphatase (ALP) staining, under incubation in osteogenic medium (Figure 9A). Interestingly, IL-1β treatment alone also induced osteogenic differentiation of MSCs in normal growth medium. This indicates that inflammation is a trigger of osteogenesis in MSCs. Notably, we found that *HRH1* gene expression was higher in MSCs 7 days after incubation with osteogenic medium than control (Figure 9B), which suggests that HRH1 constitutive activity is induced during osteogenic differentiation. In Alizarin Red S staining, we validated the calcium deposition 28 days after incubation in osteogenic medium and determined that it was accelerated by IL-1β treatment, although IL-1β did not show the calcium deposition in MSCs incubated in normal growth medium (Figure 9C). In this in vitro model, we tested the effects of cyproheptadine and found that cyproheptadine suppressed osteogenesis in the absence or presence of IL-1β in a dose-dependent manner (Figure 9, D–G, and Supplemental Figure 17, A and B). qPCR showed that *RUNX2*, a master regulator of osteogenesis, was decreased by cyproheptadine treatment 1 day after incubation in osteogenic medium with or without IL-1β (Supplemental Figure 17C). On day 7, cyproheptadine decreased *RUNX2* in MSCs incubated in growth medium with or without IL-1β and in osteogenic medium with IL-1β (Supplemental Figure 17D). Consistent with the results of ALP staining and activity assay (Figure 9, D and E), *ALPL* was decreased by cyproheptadine (Supplemental Figure 17D). Collectively, these results suggest that inflammation-induced osteogenesis in MSCs was suppressed by cyproheptadine, which may be another mechanism of inhibitory effect of cyproheptadine on osteophyte formation in the OA mouse model.

### Cyproheptadine effects on pain behavior.

**Figure 10:**
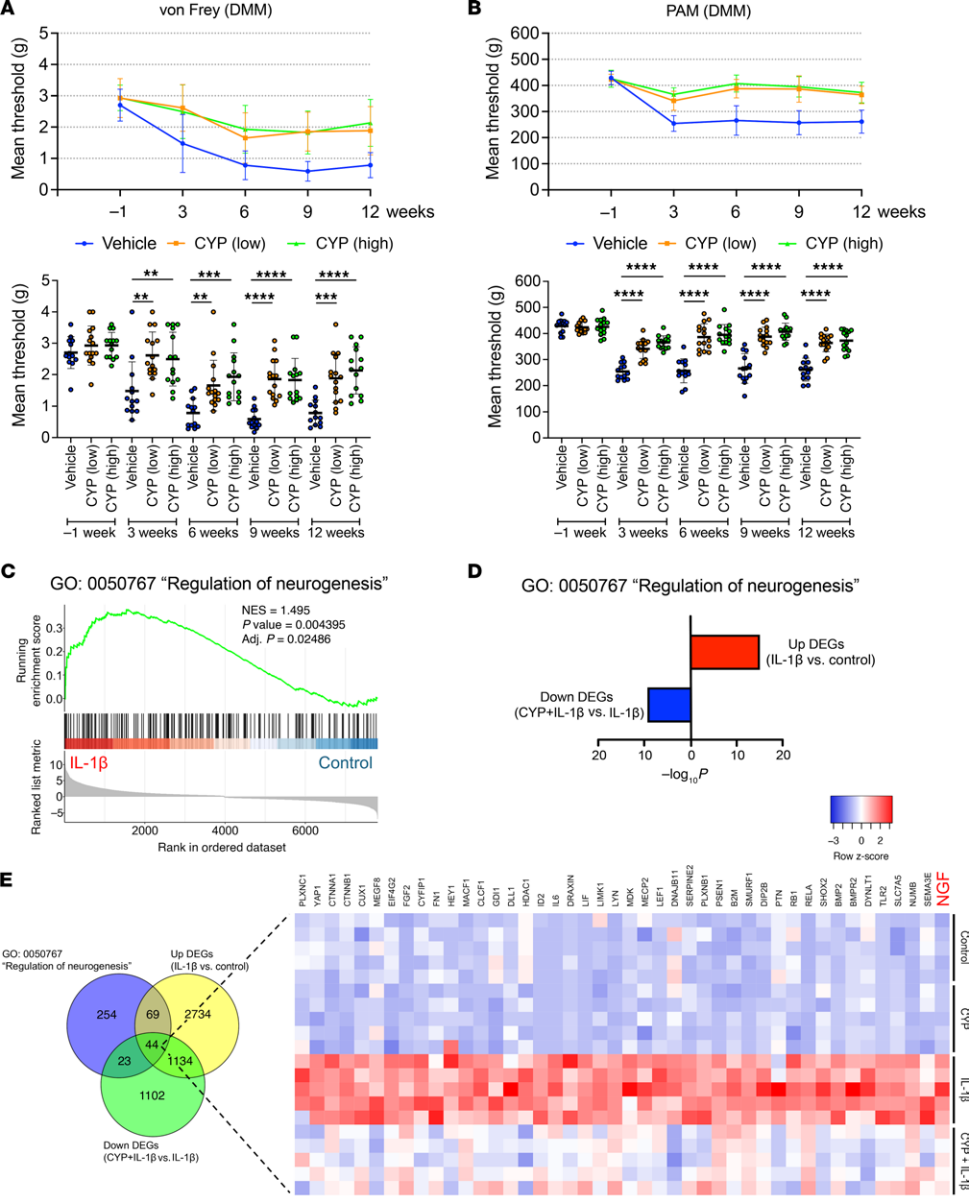
Effects of cyproheptadine on pain behaviors. Pain behaviors were evaluated by von Frey test (**A**) and pressure application measurement (PAM) (**B**) in mice treated with cyproheptadine (CYP) (low dose, 5 mg/kg; high dose, 10 mg/kg) or control vehicle after DMM surgery (Figure 2A). DMM vehicle (*n* = 13), DMM CYP (low) (*n* = 15), and DMM CYP (high) (*n* = 14). (**C**) GSEA showing induced “regulation of neurogenesis” in chondrocytes treated with IL-1β. (**D**) Enriched GO: 0050767 “regulation of neurogenesis” in the upregulated DEGs by IL-1β and the downregulated DEGs by CYP with IL-1β in RNA-seq. (**E**) Venn diagram and heat map of the shared genes in GO: 0050767 “regulation of neurogenesis”, the upregulated DEGs by IL-1β and the downregulated DEGs by CYP with IL-1β. Statistical analysis was performed using 1-way ANOVA with the Dunnett’s post hoc test. **P < 0.01, ***P < 0.001, ****P < 0.0001.

In the OA mouse model, pain behaviors were assessed by the von Frey test and pressure application measurement (PAM) (Figure 2A). In the von Frey test, the pain threshold decreased after DMM surgery (Figure 10A). In addition, the sham side also showed reduced pain threshold, which is known as mirror image pain (Supplemental Figure 18A) (35). These pain behaviors were improved by cyproheptadine treatment even at the low dose. PAM also showed that low and high dose cyproheptadine suppressed pain on the DMM side (Figure 10B and Supplemental Figure 18B). In enrichment analysis of the RNA-seq data, we found that “regulation of neurogenesis” was induced by IL-1β and suppressed by cyproheptadine (Figure 10, C and D). Notably, cyproheptadine suppressed 44 neurogenesis-related genes that were increased by IL-1β treatment, including *NGF* (Figure 10E). In qPCR, we validated that cyproheptadine, desloratadine, and HRH1 knockdown inhibited the induction of *NGF* by IL-1β in chondrocytes (Supplemental Figure 18, C–E). Furthermore, cyproheptadine and HRH1 knockdown also inhibited the induction of *NGF* gene expression by IL-1β in synoviocytes (Supplemental Figure 18, F and G).

### Cyproheptadine and ER/lipid/cholesterol homeostasis.

RNA-seq showed that cyproheptadine modulated UPR and ER stress (Figure 3C and Supplemental Figure 7, H and I). Indeed, the expression of UPR-related genes in all 3 branches, PERK, IRE1, and ATF6, were increased by cyproheptadine treatment in RNA-seq and qRT-PCR (Supplemental Figure 19, A and B). Calcium depletion in the ER triggers ER stress and thapsigargin induces ER stress (36). Since cyproheptadine rescued the calcium decrease in the ER by thapsigargin treatment (Figure 6H), we hypothesized that cyproheptadine could affect ER homeostasis. We first tested if cyproheptadine suppressed ER stress response induced after thapsigargin treatment by monitoring the expression of UPR-related genes. Thapsigargin strongly increased UPR-related genes compared with cyproheptadine, but cyproheptadine did not suppress the induction of these genes by thapsigargin (Supplemental Figure 19C).

**Figure 11:**
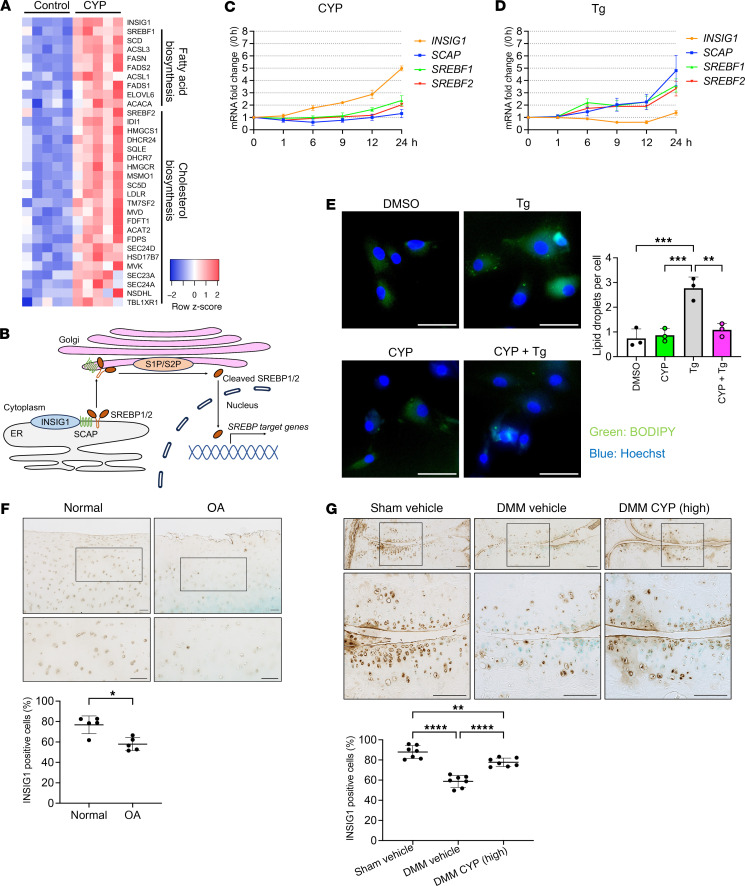
Cyproheptadine regulation of ER stress-induced lipid/cholesterol biosynthesis. (**A**) Heat map of fatty acid and cholesterol biosynthesis-related genes in RNA-seq in human chondrocytes 24 hours after treatment with cyproheptadine (CYP) (30 μM). (**B**) Diagram of 3 major regulators for lipid/cholesterol biosynthesis, SREBP1/2, SCAP, and INSIG1. Time courses of *INSIG1*, *SCAP*, *SREBF1,* and *SREBF2* in chondrocytes (*n* = 3) incubated with CYP (30 μM) (**C**) or thapsigargin (Tg) (1 μM) (**D**) for 24 hours in qRT-PCR. (**E**) BODIPY staining (*n* = 3) in chondrocytes incubated with CYP, Tg, or CYP and Tg for 24 hours. Scale bar: 50 μm. (**F**) IHC of INSIG1 in human normal and OA cartilage. *n* = 5. Scale bar: 100 μm. (**G**) IHC of INSIG1 in cartilage of mice with sham or DMM surgery treated with vehicle or CYP. *n* = 7. Scale bar: 100 μm. Data are presented as means ± SD. Statistical analysis in **E** and **G** was performed using 1-way ANOVA with the Tukey-Kramer post hoc test. Statistical analysis in **F** was performed using the Mann-Whitney test. *P < 0.05, **P < 0.01, ***P < 0.001, ****P < 0.0001.

Cholesterol metabolism was the most enriched process in RNA-seq analysis of cyproheptadine treatment in chondrocytes (Figure 3C). ER is the major site of cholesterol and fatty acid synthesis, and ER stress is a known trigger of lipogenesis (37). Therefore, we analyzed the effects of cyproheptadine on ER stress–induced lipogenesis and cholesterol biosynthesis by thapsigargin treatment. First, we checked the expression of genes related to fatty acid biosynthesis and cholesterol biosynthesis in RNA-seq data and found that many genes were upregulated by cyproheptadine (Figure 11A). Cellular cholesterol levels are strictly regulated by a feedback loop and there are 3 major regulators, SREBP2, SREBF chaperone (SCAP), and insulin induced gene 1 (INSIG1) (38) (Figure 11B). When cholesterol depletion occurs, the SREBP2-SCAP complex translocates from the ER to the Golgi. SREBP2 then undergoes cleavage by S1P and S2P, and cleaved SREBP2 is subsequently transported to the nucleus and induces genes that increase cholesterol biosynthesis. When cholesterol levels are high, INSIG1 interacts with SCAP, retaining the SREBP2-SCAP complex in the ER. SREBP1 is a transcription factor involved in upregulating cellular fatty acid levels and its activity is regulated by the same mechanism with SCAP and INSIG1 (39). Our RNA-seq data showed that *INSIG1* and *SREBF1/2* expression was increased after treatment of cyproheptadine for 24 hours, but *SCAP* was not changed (Figure 11A). We examined whether the effect of cyproheptadine on the expression of these genes was mediated by the inhibition of HRH1 signaling. Desloratadine treatment increased *INSIG1*, *SREBF1,* and *SREBF2* (Supplemental Figure 20A). HRH1 knockdown also increased *INSIG1*, *SREBF1,* and *SREBF2* (Supplemental Figure 20B). *SCAP* was not changed by desloratadine treatment and HRH1 knockdown. We further tested the time course of these gene expressions after cyproheptadine treatment for 24 hours (Figure 11C). *INSIG1* expression was elevated at 6 hours and most strongly increased at 24 hours. *SREBF1* and *SREBF2* were increased at 24 hours. *SCAP* was not changed. We next checked the expression pattern of these genes after thapsigargin treatment (Figure 11D). After 6 hours, *SCAP*, *SREBF1,* and *SREBF2* genes were elevated and gradually increased thereafter. On the other hand, *INSIG1* was reduced at 9 hours. These results indicate that cyproheptadine suppresses, while thapsigargin enhances, lipogenesis and cholesterol biosynthesis. Indeed, cyproheptadine inhibited the formation of lipid droplets induced by thapsigargin (Figure 11E). Filipin staining showed that cyproheptadine also inhibited the increase of cellular cholesterol levels induced by thapsigargin (Supplemental Figure 21A). We then examined the interaction of cyproheptadine and thapsigargin on these genes and found that the reduction of *INSIG1* by thapsigargin was rescued by cyproheptadine, but the increases in *SCAP*, *SREBF1,* and *SREBF2* by thapsigargin were not suppressed by cyproheptadine (Supplemental Figure 21B). Consistently, immunocytochemistry showed that cyproheptadine increased INSIG1 protein levels and prevented the reduction of INSIG1 by thapsigargin treatment. We also found that the co-localization of INSIG1 and SREBP1 was induced by cyproheptadine and the nuclear localization of SREBP1 induced by thapsigargin was inhibited by cyproheptadine (Supplemental Figure 21C). Therefore, we hypothesized that INSIG1 plays a key role in the regulation of lipid and cholesterol metabolism by cyproheptadine. Notably, we found that the expression of INSIG1 in human OA cartilage was lower than in healthy cartilage (Figure 11F). In the mouse DMM model, the levels of INSIG1 were reduced in OA cartilage and cyproheptadine treatment maintained its expression (Figure 11G).

## Discussion

The goal of this study was to discover FOXO activators in chondrocytes and to test their efficacy as DMOADs in cells and an OA animal model. In our initial screening approach, we tested candidate compounds for effects on nuclear retention of FOXO1 and FOXO3. We found that the effects on FOXO1 and FOXO3 were different depending on compound and time, with selinexor increasing both FOXOs while BEZ235 and cyproheptadine only increased FOXO3. Subsequent testing showed that cyproheptadine had an overall more promising activity profile and was selected for further analyses. Systemic cyproheptadine administration to mice with experimental OA ameliorated cartilage damage, synovitis, osteophyte formation, and pain behaviors. Analysis of the mouse knee joints showed that cyproheptadine rescued the decreased expression of FOXO3 in OA cartilage, supporting this mechanistic linkage, as observed in vitro. We also showed that cyproheptadine has other desirable activities to control OA pathways, independent of FOXO3, establishing proof of concept that cyproheptadine modulates both FOXO-dependent and FOXO-independent mechanisms in OA.

Although cyproheptadine is clinically used as an H1 antihistamine, its targets include not only HRH1 but also serotonin and muscarinic acetylcholine receptors (24). Since there have been no reports that cyproheptadine promotes the nuclear translocation of FOXO3, it was unclear through which pathways cyproheptadine regulates the activity of FOXO. We performed RNA-seq to explore mechanisms and observed that cyproheptadine strongly affects the ER. Calcium release from the ER is a shared effect of histamine, serotonin, and muscarinic receptors (26). Of these receptors, only HRH1 was expressed in chondrocytes. This suggested that HRH1 is the likely target of cyproheptadine in chondrocytes, which is further supported by experiments with HRH1 knockdown in chondrocytes. We also found that the second-generation H1 antagonist desloratadine promoted nuclear translocation of FOXO3 and increased FOXO target genes. These findings demonstrate that histamine H1 antagonists promote nuclear translocation of FOXO.

We found that chondrocytes do not express histidine decarboxylase, an enzyme required for histamine production, suggesting that chondrocytes are not capable of secreting histamine. Mast cells are the major histamine-producing cell type in peripheral tissues and mast cell–derived histamine may be increased in OA joints (40). However, in discussing the effects of cyproheptadine in chondrocytes, constitutive activity of HRH1 should be considered as an important mechanism because the effects of cyproheptadine on FOXO activity were observed in the absence of histamine. HRH1 has constitutive activity and can spontaneously send signals in the absence of ligands (25). In allergic states, the expression of HRH1 is increased, thereby facilitating constitutive activity (41). Furthermore, most antihistamines currently in clinical use are known to act as inverse agonists (25). Our observations showed increased expression of HRH1 in OA cartilage, indicating enhanced constitutive activity of HRH1. Conversely, HRH1 knockdown enhanced the nuclear expression of FOXO3 and increased FOXO target genes, also in the absence of histamine. Collectively, these results indicate that increased constitutive activity of HRH1 in OA suppresses FOXO activity and that a main mechanism that mediates the protective effects of cyproheptadine is by its acting as an inverse agonist.

Intracellular calcium plays a central role in HRH1 signaling (42). We found that the increase in total intracellular calcium levels induced by histamine was suppressed by cyproheptadine. Cyproheptadine treatment alone mildly increased intracellular calcium concentration. Intracellular calcium is in constant flux between ER and cytoplasm, even in the resting state (43). Accordingly, thapsigargin treatment can increase intracellular calcium levels of cells in the resting state by inhibiting the constant calcium uptake into the ER via SERCA. The cyproheptadine-induced total intracellular calcium levels might also be due to the feedback loop of intracellular calcium dynamics in response to the decrease in cytoplasmic calcium concentration by dampening the homeostatic calcium release from the ER into the cytoplasm via IP3. The result that cyproheptadine pretreatment suppressed the increase in intracellular calcium levels by thapsigargin also supports the notion that cyproheptadine enhances the retention of calcium in the ER. These results suggest that the constitutive activity of HRH1 promotes calcium efflux from the ER to the cytoplasm and that inverse agonism by cyproheptadine suppresses calcium signaling in the cytoplasm. Furthermore, cyproheptadine and HRH1 knockdown suppressed the expression of calcium binding proteins, especially calmodulin, which is the major mediator in calcium signaling and increased in OA cartilage. Finally, experiments with ionomycin confirmed that cyproheptadine activates FOXO by inhibiting cytoplasmic calcium signaling–mediated AKT activity.

In addition to the activation of FOXO, we found that HRH1 signaling regulates several key OA pathways. HRH1 inhibition suppressed inflammation in chondrocytes not only under histamine stimulation but also under IL-1β stimulation. The attenuation of IL-1β–induced inflammatory responses by inhibiting receptors for other ligands is an interesting observation. As IL-1β is a potent stimulus of OA-promoting factors (44), the finding that cyproheptadine was able to inhibit the effects of IL-1β illustrates its promise as an OA drug candidate. In addition to chondrocytes, cyproheptadine suppressed the effects of histamine and of IL-1β in synovial cells. Thus, cyproheptadine is effective against inflammation induced by stimuli other than histamine, and these mechanisms are also explained by inhibition of the constitutive activity of HRH1. Activation of NF-κB is a consequence of HRH1 constitutive activity (25), and our RNA-seq results showed that cyproheptadine suppressed NF-κB target genes. We initially investigated calcium signaling as a mechanism of antiinflammatory action by cyproheptadine. However, ionomycin treatment failed to suppress the antiinflammatory effect of cyproheptadine. Therefore, we explored other pathways and focused on PKC, which is a recognized target along with IP3 in HRH1 signaling (25). PLC activated by the HRH1 signal hydrolyzes phosphatidylinositol 4,5-bisphosphate (PIP2) to generate IP3 and diacylglycerol (DAG) (Figure 8B). DAG activates PKC, which, in turn, activates NF-κB. Our results, that PMA, a PKC activator, cancelled the antiinflammatory effect of cyproheptadine, and that cyproheptadine inhibited PMA-induced NF-κB activation, indicated that the main pathway of inflammation modulated by the HRH1 signal is through PKC activation. A previous study showed that PKC was activated in damaged cartilage and mediated NF-κB activation in chondrocytes, consistent with our observations (45).

Excessive mechanical stress is a key factor for OA pathogenesis. PIEZO1 is a mechanosensitive cation channel and mediates extracellular calcium influx in response to mechanical stress (46). PIEZO1 expression is increased in OA cartilage, and IL-1 stimulation is one mechanism to increase the expression of PIEZO1 in chondrocytes (47). In this study, IL-1β increased *PIEZO1* gene expression, and cyproheptadine and HRH1 knockdown suppressed *PIEZO1*. In addition, we found that PIEZO1 activation by Yoda1 induced AKT phosphorylation. These results suggest that HRH1 inhibition may control sensitivity to mechanical stress promoted under inflammatory conditions and rescues the FOXO3 activity suppressed by mechanical stress in OA chondrocytes.

Osteophyte formation is one of main hallmarks of OA. Cyproheptadine prevented osteophyte formation in mice. Although additional experiments, including micro-CT analysis, are required to further evaluate the effects of cyproheptadine, we found some mechanisms of the inhibitory effects of cyproheptadine on osteophyte formation. In chondrocytes, IL-1β stimulation increased more than 100 ossification-related genes and cyproheptadine suppressed 56 genes, including *YAP1*, *LEF1*, *PTGS2*, *PTGER4*,and *BMP2,* which are involved in osteophyte formation. These genes were also suppressed by HRH1 knockdown. YAP1, LEF1, PTGS2, and BMP2 were also reported to be regulated by PIEZO1. Combined with recent findings that PIEZO1 is involved in osteogenesis (48) and osteophyte formation (49), our results suggest that cyproheptadine suppressed osteophyte formation by inhibiting inflammation- and mechanical stress–mediated ossification. In addition, we demonstrated that cyproheptadine affects MSCs, which are thought to be responsible for osteophyte formation (50). HRH1 gene expression was increased during osteogenesis in MSCs, and cyproheptadine suppressed osteogenesis induced by IL-1β in a dose-dependent manner. These results indicate that HRH1 constitutive activity contributes to osteophyte formation under inflammation.

In the mouse OA model, cyproheptadine suppressed pain behaviors. Notably, cyproheptadine suppressed pain behaviors even at a low dose and also suppressed mirror image pain on the sham side, suggesting that cyproheptadine may act directly on mechanisms of pain, independent of its effects on joint tissues and structural changes. Indeed, previous studies have reported analgesic effects of cyproheptadine in other pain models (51, 52). It is a possible that, in addition to HRH1, cyproheptadine acts on receptors that are involved in pain, such as serotonin and muscarinic acetylcholine receptors, which are expressed in neuronal tissues. However, we found that cyproheptadine suppressed many neurogenesis-related genes increased by IL-1β in chondrocytes. This included NGF, an important target for pain control in OA (53). We found that cyproheptadine, desloratadine, and HRH1 knockdown suppressed IL1β-induced *NGF* expression in chondrocytes, and cyproheptadine and HRH1 knockdown had a similar effect in synoviocytes. These observations, showing that, under inflammatory conditions, NGF expression could be controlled by HRH1 suppression, indicate that suppression of HRH1 in cells of the knee joint may be one mechanism mediating the analgesic effect of cyproheptadine.

Our studies on signaling mechanisms revealed that effects of cyproheptadine occurred also via the retention of calcium in the ER, in addition to the inhibition of cytoplasmic calcium signaling. Calcium depletion in the ER is a trigger of ER stress (36). We then focused on the relationship between cyproheptadine and ER stress in cholesterol metabolism because, in RNA-seq, the expression of genes related to cholesterol/lipid metabolism was most strongly altered by cyproheptadine treatment, and ER stress is known to enhance lipogenesis (37). Excessive intracellular lipid and cholesterol accumulation in chondrocytes has also been suggested to be involved in OA (54, 55). Our results showed that thapsigargin promoted intracellular lipid droplet formation and cholesterol biosynthesis and this was inhibited by cyproheptadine. Mechanistically, we found that cyproheptadine increased the expression of INSIG1, a major inhibitor of lipogenesis and cholesterol synthesis (38). Conversely, thapsigargin decreased INSIG1 expression, but cyproheptadine rescued it. Consistent with the in vitro results, we found that INSIG1 expression was downregulated in human and mouse OA cartilage, and cyproheptadine treatment prevented suppression of INSIG1 in the mouse OA model. Mice with cartilage-specific *Insig1* deletion spontaneously develop OA (56). Hence, we considered the retention of INSIG1 expression to be one of the important mechanisms of OA attenuation by cyproheptadine.

This study demonstrates diverse effects of cyproheptadine on chondrocytes, synoviocytes, and MSCs via inhibition of the HRH1 signaling and the potential of cyproheptadine as a DMOAD. Notably, a recent study reported that patients taking oral H1 antihistamines for more than 1 year had a lower prevalence of OA (57). Although no adverse behavioral changes were observed in mice, the first-generation cyproheptadine targets receptors other than HRH1 and is likely to cause off-target adverse effects, such as drowsiness, due to crossing the blood brain barrier, thus, second-generation H1 antihistamines are now commonly used. We confirmed that desloratadine is effective similar to cyproheptadine in vitro. Furthermore, HRH1 knockdown also demonstrated similar effects, suggesting that other antihistamines may also be promising. Many types of antihistamines are already widely and safely used in clinical practice. This is a major advantage for repurposing these drugs in their development as DMOADs. To establish clinical use of HRH1 inverse agonists for OA treatment, further studies are required. Treatment can be initiated in persons at risk for OA to prevent OA or in patients with established OA to slow progression. In this study, administration of cyproheptadine was started one day after DMM surgery, a design for prevention of OA. Further studies need to test whether HRH1 inverse agonists can prevent or slow progression of established OA. In addition, considering that OA is an age-related chronic disease, the effects of drugs in an aging mouse model should be investigated. Furthermore, the optimal dose of drugs in OA patients needs to be established, although we used the similar doses of cyproheptadine as previous studies both in our in vitro and in vivo experiments (17, 58, 59).

In summary, we identified cyproheptadine as a FOXO activator and showed that it increased the expression of protective genes and suppressed OA-promoting genes. Mechanistic analysis revealed that inhibition of the constitutive activity of HRH1 prevented calcium efflux from the ER to the cytoplasm and inhibited PKC pathway. We propose that the constitutive activity of HRH1 is a promising therapeutic target for OA and that histamine H1 receptor inverse agonists are highly attractive drug candidates for OA pain and structure modification based on their effects on multiple OA mechanisms and their established safety profile.

## Methods

### Sex as a biological variable.

Our study used human tissues and primary cells from both male and female donors. In a mouse OA model, male mice were used because males develop more severe OA than females and male mice are generally used in this model (60, 61).

Detailed procedures are provided in Supplemental Materials.

### Statistics.

All data are presented as mean ± SD. All data analyses were performed using GraphPad Prism version 10.2.2 (GraphPad Software). After checking the normality using the Shapiro-Wilk test, 2-tailed paired or unpaired Student’s *t* test or the Mann-Whitney test was used for 2-group comparisons. One-way ANOVA followed by the Dunnett’s or the Tukey’s multiple comparisons post hoc test were used to compare multiple groups. Statistical tests used are all described in each figure legend. *P* values less than 0.05 were considered significant.

### Study approval.

Human tissue collection was approved by the Scripps Human Subjects Committee (IRB #22-8069). All animal experiments were approved by the Institutional Animal Care and Use Committee at Scripps Research (Protocol #09-0130-5).

### Data availability.

All individual-level data are presented in the Supporting Data Values file. RNA-seq data are available in the Gene Expression Omnibus under accession number GSE291878.

## Author contributions

IK, YA, YN, and MKL designed the overall study. IK performed in vitro experiments. IK, MO, KM, and CK performed animal studies, histological analyses, and immunohistochemistry. IK and MO performed histological scoring. IK and HS performed bioinformatic analyses. IK and MKL analyzed data and wrote the manuscript. All authors discussed the results and approved the final version of the manuscript.

## Funding support

This work is the result of NIH funding, in whole or in part, and is subject to the NIH Public Access Policy. Through acceptance of this federal funding, the NIH has been given a right to make the work publicly available in PubMed Central.

- NIH grants AG059418 and AG049617.
- The Uehara Memorial Foundation.
- The Japanese Society for the Promotion of Science (JSPS) Overseas Research Fellowships.

## Supplementary Material

Supplemental data

Unedited blot and gel images

Supplemental video 1

Supplemental video 2

Supplemental video 3

Supplemental video 4

Supplemental video 5

Supplemental video 6

Supporting data values

## Version 1 08/28/2025

In-Press Preview

## Version 2 11/03/2025

Electronic publication
