# Supplementary material for: Histamine H1 receptor inverse agonists improve structure and pain in an osteoarthritis mouse model
Source: J Clin Invest. 2025 Aug 28;135(21):e183588. doi: 10.1172/JCI183588 (PMC12578390; doi:10.1172/JCI183588)
Supplement: Supplemental data [file jci-135-183588-s174.pdf]

Supplemental Materials for

Histamine H1 receptor inverse agonists improve structure and pain in an osteoarthritis mouse model

Supplemental Methods
Supplemental Figure 1-21
Supplemental Table 1 and 2
Supplemental Video 1-6
Supplemental References

Supplemental Methods

Human tissue collection and primary cell isolation

Normal human knee joints were obtained from Lifesharing, San Diego. OA human knee cartilage and synovium were obtained from patients undergoing total knee arthroplasty. To isolate human primary chondrocytes and synoviocytes, cartilage and synovium were incubated with 2mg/ml collagenase II diluted in Dulbecco's Modified Eagle Medium (DMEM) at 37°C for 12 hours. After digestion, chondrocytes were cultured in DMEM with penicillin, streptomycin, and L-glutamine with 10% calf serum (CS). After having reached sub-confluence, the cells were used in experiments.

Cell culture

To test FOXO activator candidates, primary chondrocytes were cultured with selinexor (S7252, Selleck Chemicals), BEZ235 (S1009, Selleck Chemicals), cyproheptadine (S4635, Selleck Chemicals), LOM612 (HY-101035, Medchem express) or psammaplysene A (AOB8551, Aobious). All chemicals were dissolved in dimethyl sulfoxide (DMSO), and control groups were incubated with an equivalent amount of DMSO. Cell viability was evaluated with CellTiter-Glo 2.0 assay (Promega).

Desloratadine (S4012, Selleck Chemicals), thapsigargin (T9033, Sigma-Aldrich), ionomycin (AB120116, Abcam), Yoda1 (S6678, Selleck Chemicals) and phorbol 12-myristate 13-acetate (PMA) (AB120297, Abcam) were used for mechanistic analyses.

To model an inflammatory response, human primary chondrocytes and synoviocytes were cultured with human recombinant IL-1β (200-01B, Peprotech) or histamine (H7250, Sigma-Aldrich).

Immunocytochemistry

Human primary chondrocytes were seeded in 96-well plates at a density of 1x10^4 cells/well. After 24 hours, the cells were incubated with small molecules for 24 hours. The cells were fixed with 4% paraformaldehyde (PFA) for 10 min at room temperature and then blocked with 5% goat serum and 0.3% Triton X-100 in PBS for 1 hour. Subsequently, the cells were incubated with primary antibodies against FOXO1 (1:100; #2880, Cell Signaling Technology), FOXO3 (1:200; #2497, Cell Signaling Technology), p65 (1:400; #8242, Cell Signaling Technology), INSIG1 (1:50; sc-390504, Santa Cruz Biotechnology) and SREBP1 (1:50; 14088-1-AP, Proteintech) overnight at 4°C. The cells were washed three times with PBS and incubated with Alexa Flour 488 conjugate (1:500; A11034, Invitrogen) or Alexa Flour 568 conjugate (1:500; A11036, Invitrogen) for 1 hour at room temperature. For nuclear staining, the cells were incubated with Hoechst (1:500; 33342, Invitrogen) for 15 min. Immunostaining was visualized by wide-field fluorescence microscope (BZ-X700, Keyence). ImageJ software was used to analyze signal intensity.

RNA isolation and qRT-PCR

Total RNA was isolated with Direct-zol RNA MicroPrep kit (R2061, Zymo Research) and reverse transcribed to complementary DNA using PrimeScript RT regent kit (RR037B, Takara Bio). Real-time qPCR was performed on a CFX384 Real-Time System (Bio-Rad) using Power SYBR Green PCR Master Mix (4367659, Applied Biosystems). The sequences of the primers are shown in Supplemental Table 2. Data were normalized against the corresponding levels of glyceraldehyde-3-phosphate dehydrogenase (GAPDH).

siRNA transfection

Cells were seeded in 24-well plates at a density of 5x10^4 cells/well. After 24 hours, the cells were transfected with siRNAs (20nM) for FOXO3 (s5261, Invitrogen), HRH1(s223878, Invitrogen), and the negative control (4390843, Invitrogen) using Lipofectamine RNAiMAX (13778100, Life

Technologies) according to the manufacturer's instructions. After 48 hours, the cells were used in qRT-PCR and immunocytochemistry analyses.

Surgical induction of OA and treatment with cyproheptadine in mice

The surgical OA model of destabilization of the medial meniscus (DMM) was induced in 15 weeks-old C57BL/6J male mice by transection of the medial meniscotibial ligament in the right knee as described (1). Sham surgery was performed in the left knee by opening skin and capsule and closing with surgical suture. All mice were anesthetized with using a 2% isoflurane-O_2 mixture and received one subcutaneous injection of buprenorphine (20 µL, 1mg/mL) during the surgery. Mice were randomly assigned into the three groups, DMSO vehicle-treated group, 5mg/kg (low dose) cyproheptadine, and 10mg/kg (high dose) cyproheptadine-treated groups. Cyproheptadine was first solubilized in DMSO as a stock solution and diluted in 5% dextrose at a final concentration of 0.5 mg/mL for the 5 mg/kg group and 1 mg/mL for the 10 mg/kg group. Cyproheptadine or DMSO vehicle were administrated intraperitoneally 3 times per week for 12 weeks, starting one day after surgery.

von Frey test

To assess mechanical allodynia, the von Frey test was conducted in mice at 1 week before Sham or DMM surgery as baseline, and subsequently every 3 weeks at 3, 6, 9 and 12 weeks after the surgery. Mice were placed on a mesh metal platform and allowed 15 minutes of habituation before each test. The plantar surface of the hind paw was stimulated with using von Frey fibers and the up-down method (2). Each mouse was tested five times, and the average of the threshold was calculated. All tests were performed in a blinded manner.

Pressure application measurement (PAM)

Knee hyperalgesia was evaluated using the pressure application measurement device (PAM; Ugo Basile) (3). Mice were restrained by left hand and the hind paw was held in slight flexion with right fingers. The PAM transducer was pressed against the medial side of knee. The amount of force was increased at a constant rate (30 gram force/s) under the guidance of the PAM software. Pressure was stopped and measured in any case of paw withdrawal, or when an audible noise was observed. Two measurements were performed per knee and the withdrawal force data were averaged. PAM test was conducted on mice at 1 week before Sham or DMM surgery as a control, then every 3 weeks at 3, 6, 9 and 12 weeks after the surgery in a blinded manner.

Histological analyses

Mice were euthanized 12 weeks after surgery, and knee joints were harvested and fixed in Z-Fix (Anatech) for 2 days at room temperature. After decalcification in a formic acid solution (TBD-2, Shandon) for 2 days, the samples were embedded in paraffin and sectioned at 4 μm thickness. Safranin-O and fast green staining was performed, and OA related changes were scored as previously described for articular cartilage degradation (summed OARSI score for femur and tibia) on a 0-48 scale (4), synovitis on a 0-9 scale (5), and osteophyte maturation on a 0-3 scale (6). All scorings were performed by two different individuals in a blinded manner.

Immunohistochemistry

The sections from human cartilage and mouse knee joints were deparaffinized, rehydrated; antigen retrieval was performed by incubation with 10mM sodium citrate (pH6.0) at 70°C for 30 min. Endogenous peroxidase activity was blocked by incubation with Bloxall Solution (Vector Laboratories) for 10 min. After blocking with 2.5% normal horse serum for 1 hour, sections were incubated with primary antibodies against FOXO3 (1:50; #12829, Cell Signaling Technology), IL-6 (1:600; ab6672, Abcam), HRH1 (1:200; bs-6663R, Bioss) and INSIG1 (1:100; ab70784, Abcam)

overnight at 4°C. Rabbit IgG was used as a negative control. After washing with TBST, sections were incubated with ImmPRESS anti-rabbit IgG kit reagent (MP-7401, Vector Laboratories) at room temperature for 30 min. Antibody complexes were visualized by incubation with DAB and counterstained with methyl green. For quantification of positive cells, we observed fields in the medial femoral and tibial cartilage of mouse knees and fields within 1mm from the articular surface in human cartilage tissues. The percentage of positive cells was determined using the BZ-II Analyzer software (Keyence). The same signal threshold was applied for each group of comparable images.

RNA-seq analysis

Human primary chondrocytes were incubated with cyproheptadine (30 μM) or DMSO for 24 hours. For IL-1β stimulation, cells were incubated with IL-1β (1 ng/mL) for 6 hours following the pretreatment with cyproheptadine (30 μM) for 24 hours. Total RNA was isolated with Direct-zol RNA MicroPrep kit. RNA quality was confirmed by measuring RNA integrity number (RIN) using Bioanalyzer (Agilent) and all samples had RIN > 9.4. cDNA libraries were generated and sequenced on Illumina NextSeq 2000 instrument (Illumina) at an estimated 5 million reads per sample. Quality control of the fastq files was performed using FastQC (v0.11.8). Illumina 3' small adapter sequences was trimmed using Trim Galore! (v0.6.4). Trimmed reads were aligned to the human genome (GRch38) using STAR (v2.6.1d). Genomic features were assigned using featureCounts (v1.6.4). Differential expression was determined using DESeq2 (v1.20.0). The resulting p values were adjusted using the Benjamini-Hochberg's approach for controlling the false discovery rates (FDR). Genes with an FDR <0.05 were considered statistically significant. Functional enrichment analyses and the TRRUST analyses with differentially expressed genes

(DEGs) were performed using Metascape (7). Gene set enrichment analysis (GSEA) was also performed.

Western blotting

Whole-cell lysates were prepared from human chondrocytes using ice-cold RIPA buffer (89900, Thermo Scientific) with protease inhibitor (1862209, Thermo Scientific) and phosphatase inhibitor (1862495, Thermo Scientific). Total protein was quantified using BCA assay (23227, Thermo Scientific). Cell lysates were electrophoresed in 4-12% acrylamide gels (NW04122, Invitrogen) and the resolved proteins were transferred to nitrocellulose membrane. Membranes were blocked with blocking buffer, washed in TBST, and incubated with primary antibodies against LC3 (1:1000; #3868, Cell Signaling Technology), p65 (1:1000; #8242, Cell Signaling Technology), phosphorylated p65 (1:1000; #3033, Cell Signaling Technology), AKT (1:2000; #2920, Cell Signaling Technology), phosphorylated AKT (1:1000; #4060, Cell Signaling Technology) and GAPDH (1:2000; AM4300, Invitrogen) diluted in LI-COR antibody diluent overnight at 4℃. After incubating with secondary anti-rabbit antibody (1:5000; 926-32211, LI-COR) or anti-mouse antibody (1:20000; 926-68070, LI-COR) for 1 hour at room temperature, blots were visualized using LI-COR Odyssey imaging system. Band intensity values were analyzed using ImageJ software and normalized to those of GAPDH.

Live-cell intracellular calcium imaging

TC28 cells were purchased from Sigma-Aldrich and cultured in DMEM with penicillin, streptomycin, and L-glutamine with 10% CS. After having reached sub-confluence, the cells were seeded in 96-well plates with black wall and clear bottom at a density of $1x10^4$ cells/well in 0.5% CS medium. After 24 hours, the cells were loaded with Fluo-8 (ab112129, Abcam) in Hank's buffer with Hepes (HHBS) for 30 min at 37℃ in the presence or absence of cyproheptadine (30

μM) and then incubated at room temperature for another 30 min. Fluorescence was observed using Ti2-E widefield fluorescence microscope (Nikon). Calcium transients were induced by histamine (10 μM), cyproheptadine (30 μM), thapsigargin (1 μM) or ionomycin (0.2 μM). Signal intensity was recorded every 3 sec using NIS-Elements software (Nikon) for 30 sec as a baseline, and then for 5 min after each stimulation. Fluorescence was expressed as the ratio of the signal change to the initial intensity ($\Delta F/F_0$). Representative videos are shown in the Supplemental Materials.

Osteogenesis in mesenchymal stem cells (MSCs)

Normal human bone marrow derived MSCs were purchased from Lonza and seeded in growth medium containing mesenchymal cell growth supplement (MCGS), L-glutamine and GA-1000 (PT-3001, Lonza). To induce osteogenesis, cells were allowed to adhere for 24 hours and then incubated in osteogenic differentiation medium containing MCGS, dexamethasone, L-glutamine, ascorbate, β-glycerophosphate and penicillin/streptomycin (PT-3002, Lonza), with cyproheptadine and/or IL-1β. Growth medium was used as control medium. Medium was replaced every 3 days. MSCs were used by passage 5 in accordance with the manufacturer's protocol.

Alkaline phosphatase (ALP) assay

ALP staining was performed by using TRACP and ALP double-stain Kit (MK300, Takara Bio) according to the manufacturer's protocol. Briefly, MSCs were seeded in 48 well plates at a density of 3,500 cells/well and cultured in osteogenic differentiation medium with cyproheptadine and/or IL-1β for 7 days. Fixed MSCs were incubated with substrate solution for ALP for 45 min at 37℃. After washing three times with distilled water to stop the reaction, wells were photographed using wide-field fluorescence microscope (BZ-X700, Keyence).

For quantification of ALP activity, TRACP and ALP Assay Kit (MK301, Takara Bio) was used. MSCs were seeded in 96 well plates at a density of 1,000 cells/well and cultured in osteogenic

differentiation medium with cyproheptadine and/or IL-1β for 7 days. ALP activity was measured based on the absorbance at 405 nm and was normalized to the total protein concentration determined by BCA assay.

Alizarin red staining

After 28 days of culture in osteogenic induction, MSCs were fixed with 10% formalin for 10 min. After washing with distilled water, cells were stained with alizarin red S (A5533, Sigma-Aldrich) for 10 min. The stained cells were washed five times with distilled water and observed using wide-field fluorescence microscope (BZ-X700, Keyence). To quantify the staining, 10% cetylpyridinium chloride (C5460, Sigma-Aldrich) was added to each well and incubated for 15 min to extract the staining dye. The absorbance at 562 nm was measured and normalized to the total protein concentration determined by BCA assay.

Filipin staining

Cellular cholesterol in human primary chondrocytes was stained using Cholesterol Cell-Based Detection Assay Kit (10009779, Cayman Chemical) according to the manufacturer's instructions. In brief, human primary chondrocytes were seeded in 96-well plates at a density of $1x10^4$ cells/well. After 24 hours, the cells were incubated with cyproheptadine (30 μM) and/or thapsigargin (1 μM) for 24 hours. The cells were fixed with Cell-Based Assay Fixative solution for 10 min at room temperature, washed three times, and then incubated with Filipin III Staining solution for 1 hour in the dark. After washing two times, fluorescence was observed using wide-field fluorescence microscope (BZ-X700, Keyence). ImageJ software was used to analyze signal intensity.

BODIPY staining

Cellular lipid droplets in human primary chondrocytes were stained using BODIPY™ 493/503 (D3922, Invitrogen) according to the manufacturer's instructions. In brief, human primary

chondrocytes were seeded in 96-well plates at a density of 1x10^4 cells/well. After 24 hours, the cells were incubated with cyproheptadine (30 μM) and/or thapsigargin (1 μM) for 24 hours. The cells were fixed with 4% PFA for 10 min at room temperature, washed three times, and then incubated with BODIPY solution (10 μM) for 15 min in the dark. After washing three times, for nuclear staining, the cells were incubated with Hoechst for 15 min. Fluorescence was observed by wide-field fluorescence microscope (BZ-X700, Keyence). ImageJ software was used to analyze fluorescence.

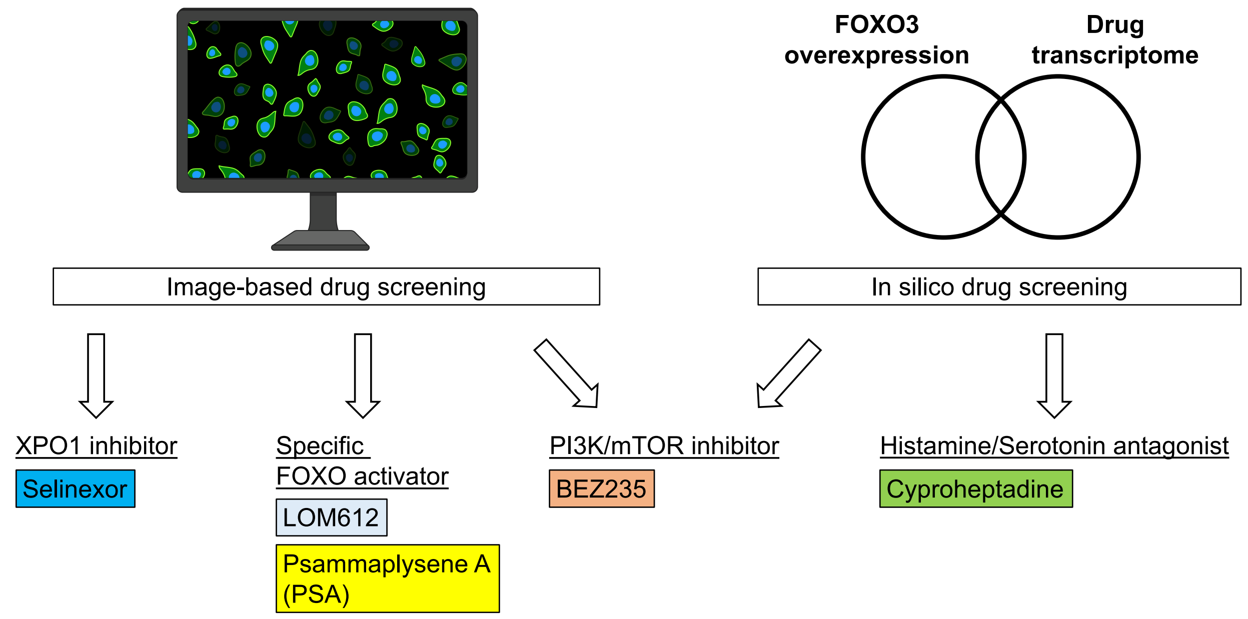

Supplemental Figure 1. FOXO activator candidates selected in this study.

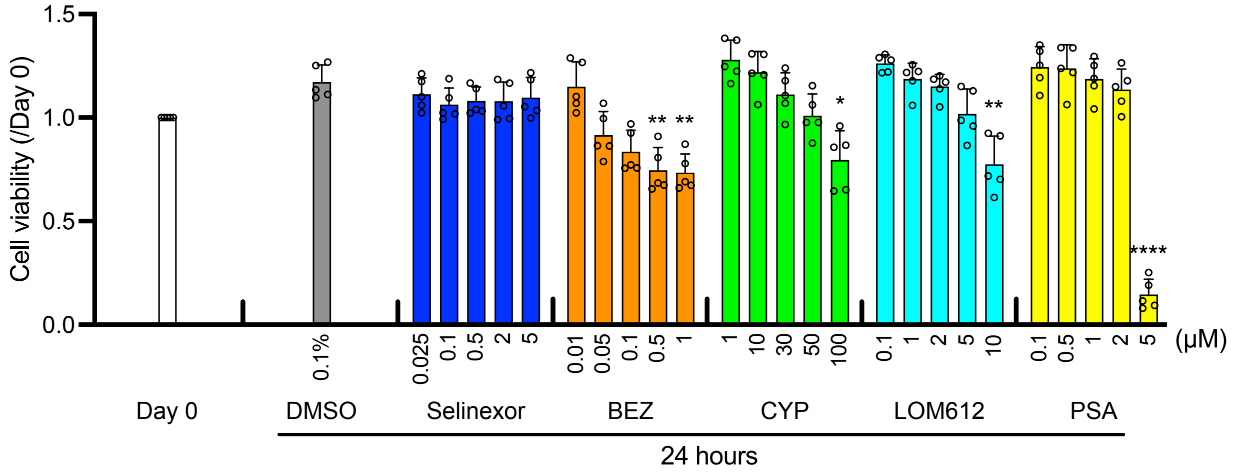

Supplemental Figure 2. Cell viabilities of human chondrocytes after treatment with FOXO activator candidates.

Cell viabilities of human chondrocytes (n = 5) were analyzed at 0 and 24 hours after treatment with DMSO (0.1%), selinexor (0.025, 0.1, 0.5, 2, 5 μM), BEZ235 (BEZ) (0.01, 0.05, 0.1, 0.5, 1 μM), cyproheptadine (CYP) (1, 10, 30, 50, 100 μM), LOM612 (0.1, 1, 2, 5, 10 μM) or psammaplysene A (PSA) (0.1, 0.5, 1, 2, 5 μM). Data are expressed as the percent luminescence of cells in each group relative to signals at Day 0. Data are presented as means ± SD. Statistical analysis was performed using one-way ANOVA with the Dunnett's post hoc test. *P < 0.05, **P < 0.01, ****P < 0.0001.

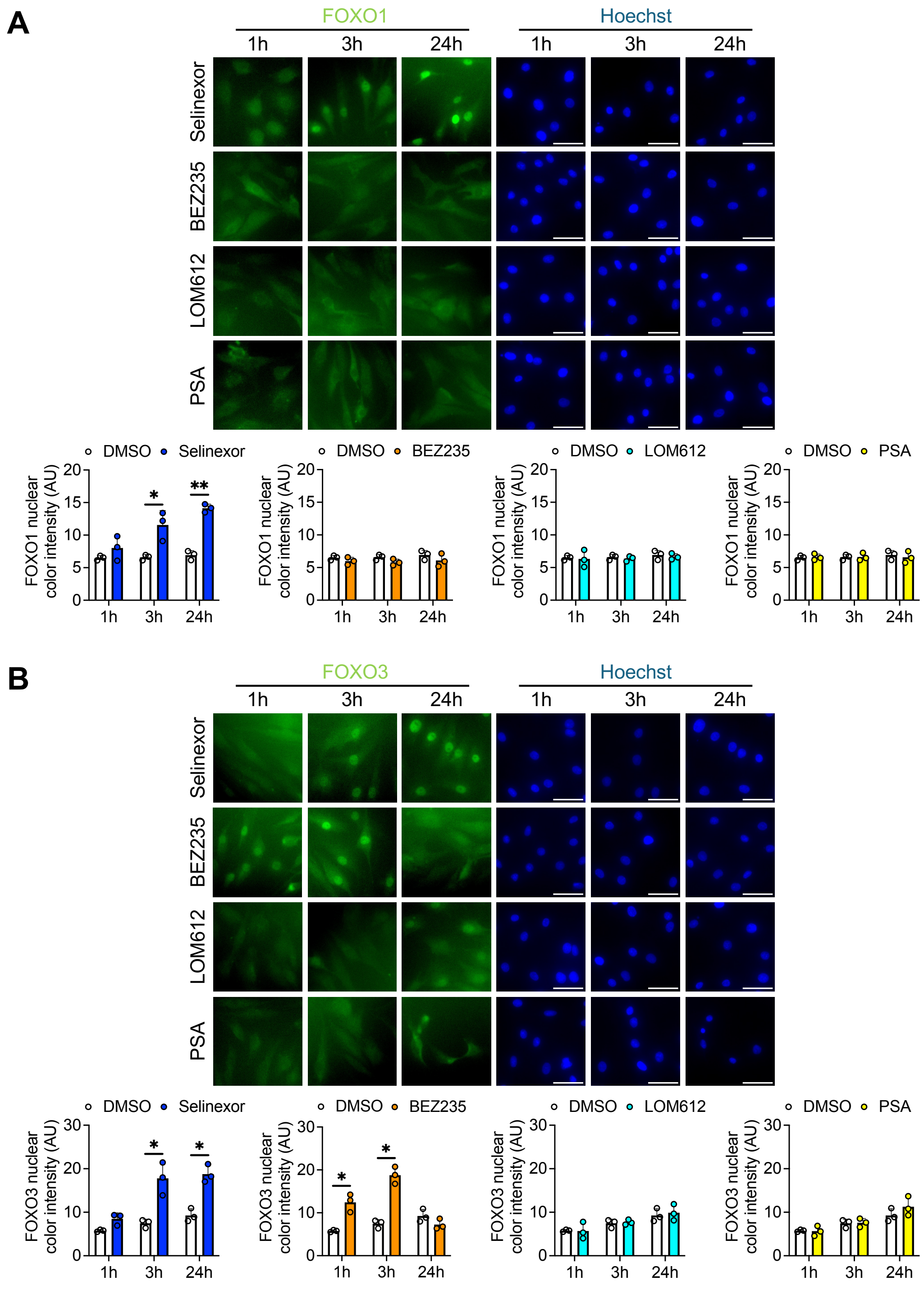

Supplemental Figure 3. Effects of selinexor, BEZ235, LOM612 and psammaplysene A on FOXO localization.

FOXO1 (**A**) and FOXO3 (**B**) localization in human chondrocytes (n = 3) 24 hours after treatment with selinexor (2 μM), BEZ235 (0.1 μM), LOM612 (5 μM) or psammaplysene A (PSA) (2 μM) in immunocytochemistry. Scale bar, 50 μm. Nuclear color intensity of FOXO1 and FOXO3 was quantified. Statistical analysis was performed using Student's t-test. *$P < 0.05$, **$P < 0.01$.

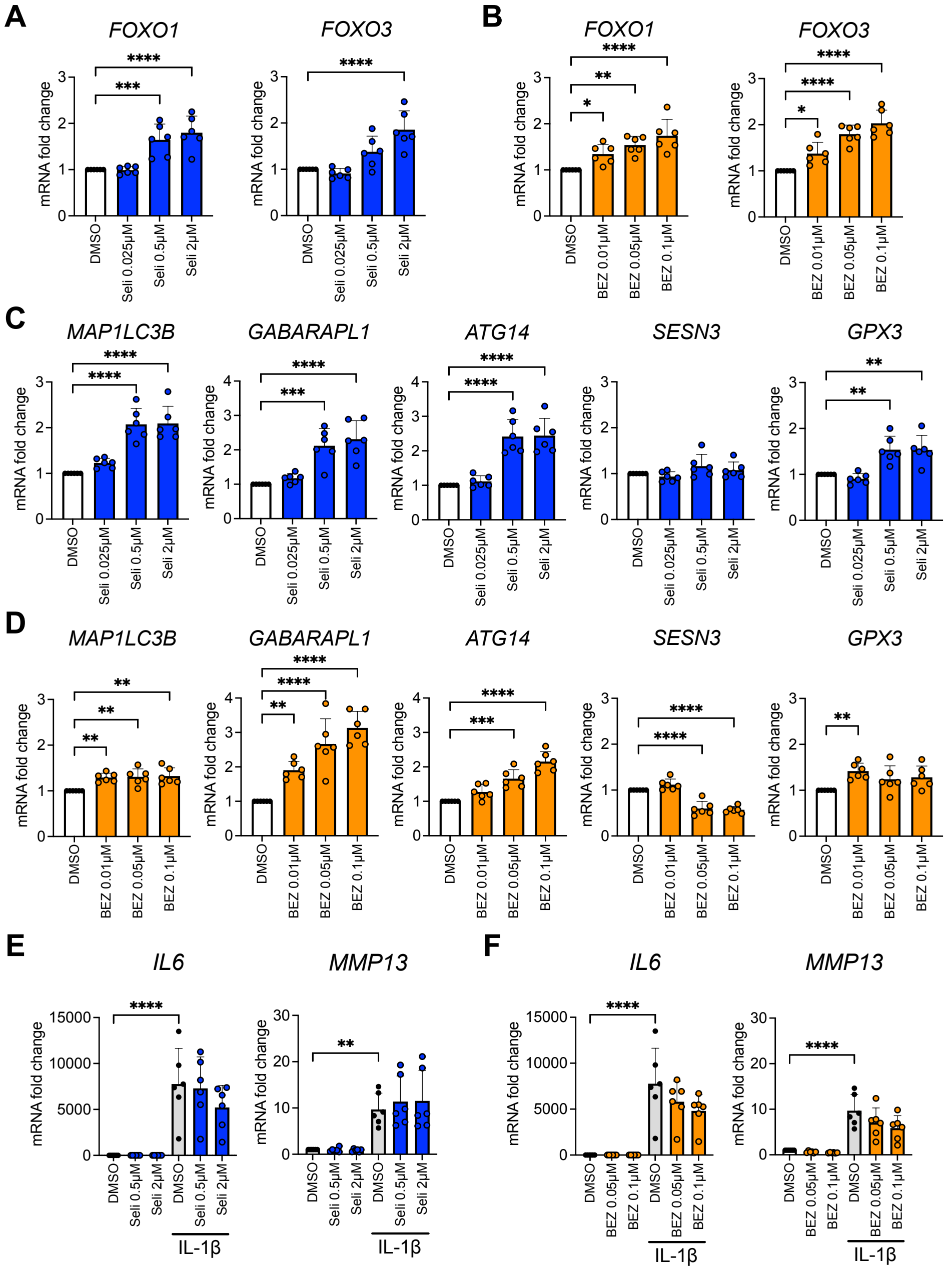

Supplemental Figure 4. Effects of selinexor and BEZ235 in human chondrocytes. Human chondrocytes (n = 6) were incubated with the indicated doses of selinexor (Seli) or BEZ235 (BEZ) for 24 hours. RNA was isolated for qRT-PCR for *FOXO1* and *FOXO3* genes after the incubation with Seli (**A**) and BEZ235 (**B**) and FOXO target genes after the incubation with Seli (**C**) and BEZ235 (**D**). Relative mRNA levels of *IL6* and *MMP13* in human chondrocytes (n = 6) incubated with IL-1β (1 ng/mL) for 6 hours after pretreatment with or without the indicated doses of Seli (**E**) and BEZ235 (**F**) for 24 hours in qRT-PCR. Data are presented as means ± SD. Statistical analysis in (A to D) was performed using one-way ANOVA with the Dunnett's post hoc test. Statistical analysis in (E) and (F) was performed using one-way ANOVA with Tukey-Kramer post hoc test. *$P < 0.05$, **$P < 0.01$, ***$P < 0.001$, ****$P < 0.0001$.

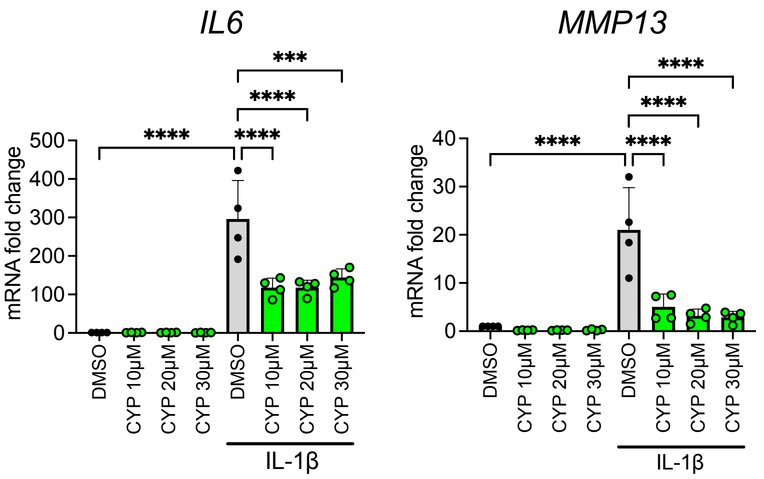

Supplemental Figure 5. Anti-inflammatory effect of cyproheptadine in human synoviocytes.

Human synoviocytes (n = 4) were incubated with IL-1β (1ng/mL) for 6 hours after pretreatment with or without the indicated doses of cyproheptadine (CYP) for 24 hours and RNA was isolated for qRT-PCR for *IL6* and *MMP13* genes. Statistical analysis was performed using one-way ANOVA with the Tukey-Kramer post hoc test. ***$P < 0.001$, ****$P < 0.0001$.

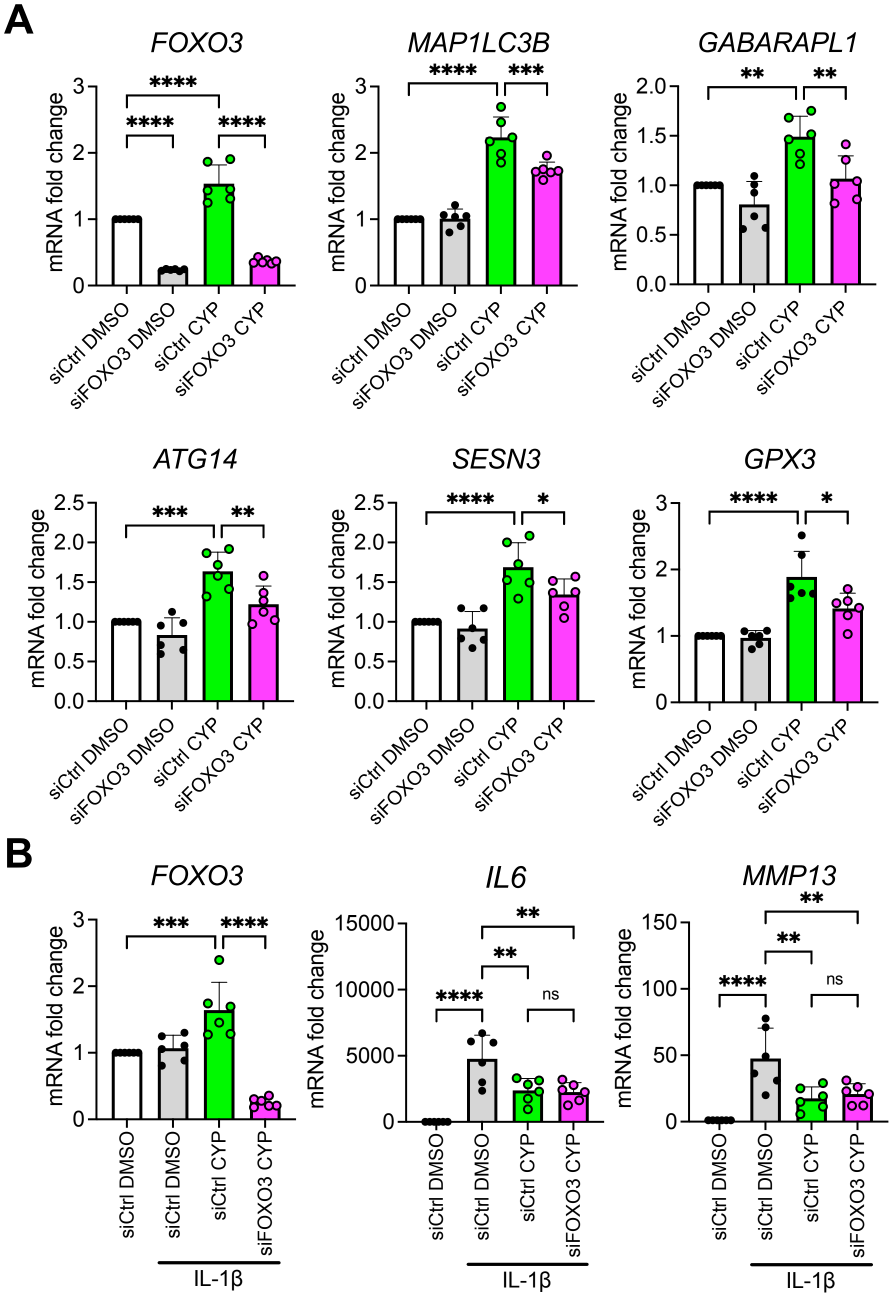

Supplemental Figure 6. Effects of FOXO3 knockdown on the expression of autophagy and antioxidant genes induced by cyproheptadine treatment.
(**A**) Human chondrocytes (n = 6) were incubated with cyproheptadine (CYP) (30 μM) for 24 hours after transfection with siCtrl or siFOXO3. Relative mRNA levels of autophagy genes and antioxidant genes were measured by qRT-PCR. (**B**) Human chondrocytes (n = 6) were incubated with IL-1β (1ng/mL) for 6 hours after pretreatment with or without CYP (30 μM) for 24 hours after transfection with siCtrl or siFOXO3 and RNA was isolated for qRT-PCR for *IL6* and *MMP13* genes. Statistical analysis was performed using one-way ANOVA with the Tukey-Kramer post hoc test. *$P < 0.05$, **$P < 0.01$, ***$P < 0.001$, ****$P < 0.0001$.

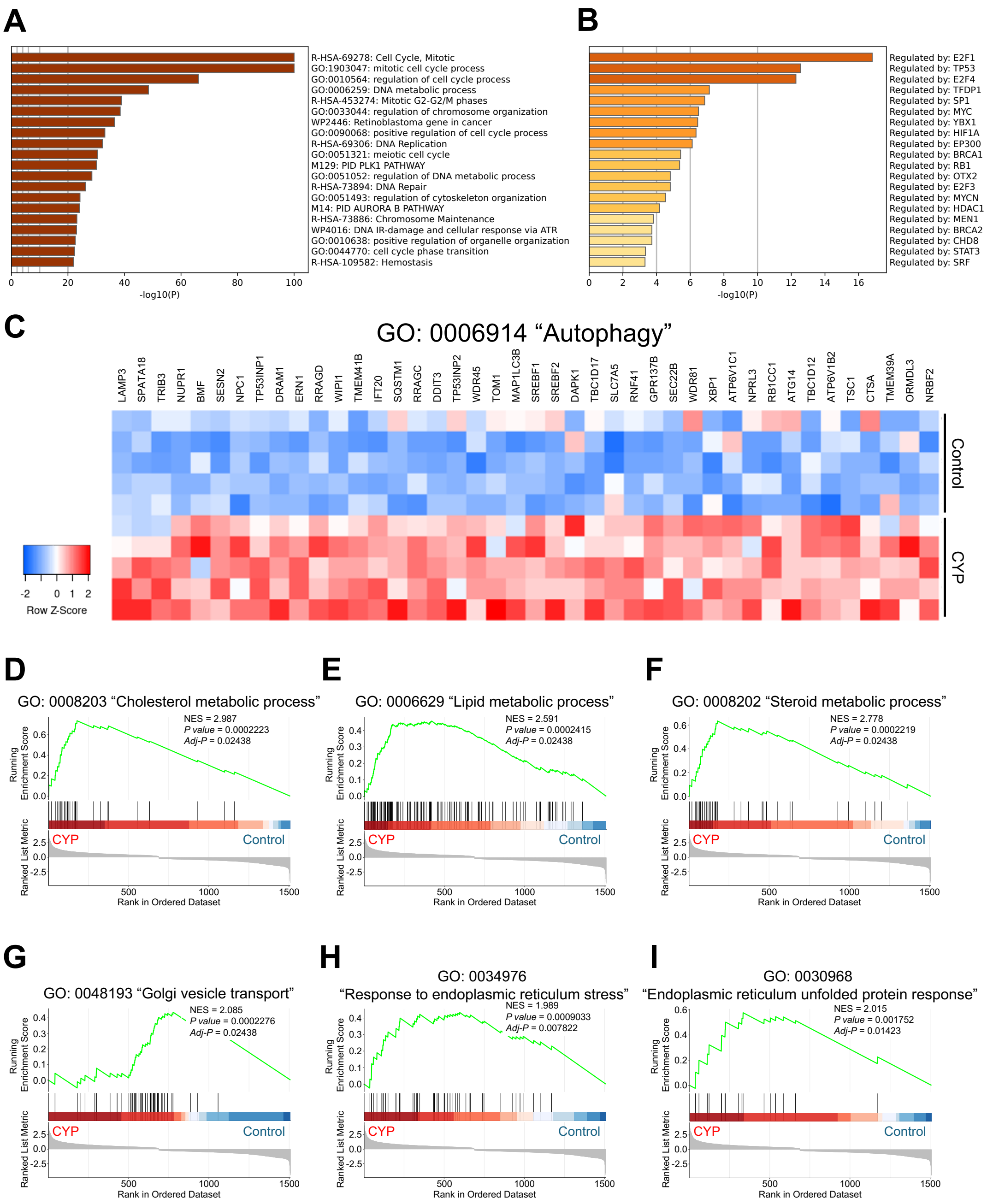

Supplemental Figure 7. Transcriptomic changes induced by cyproheptadine in human chondrocytes.

RNA-seq was performed on human chondrocytes (n = 5) treated with cyproheptadine (30 μM) for 24 hours. Metascape enrichment (**A**) and TRRUST (**B**) analysis using the downregulated genes after cyproheptadine treatment. (**C**) Heatmap of autophagy-related genes induced in chondrocytes treated with CYP. GSEA showing enrichment of "cholesterol metabolic process" (**D**), "lipid metabolic process" (**E**), "steroid metabolic process" (**F**), "golgi vesicle transport" (**G**), "response to endoplasmic reticulum stress" (**H**) and "endoplasmic reticulum unfolded protein response" (**I**) in chondrocytes treated with CYP.

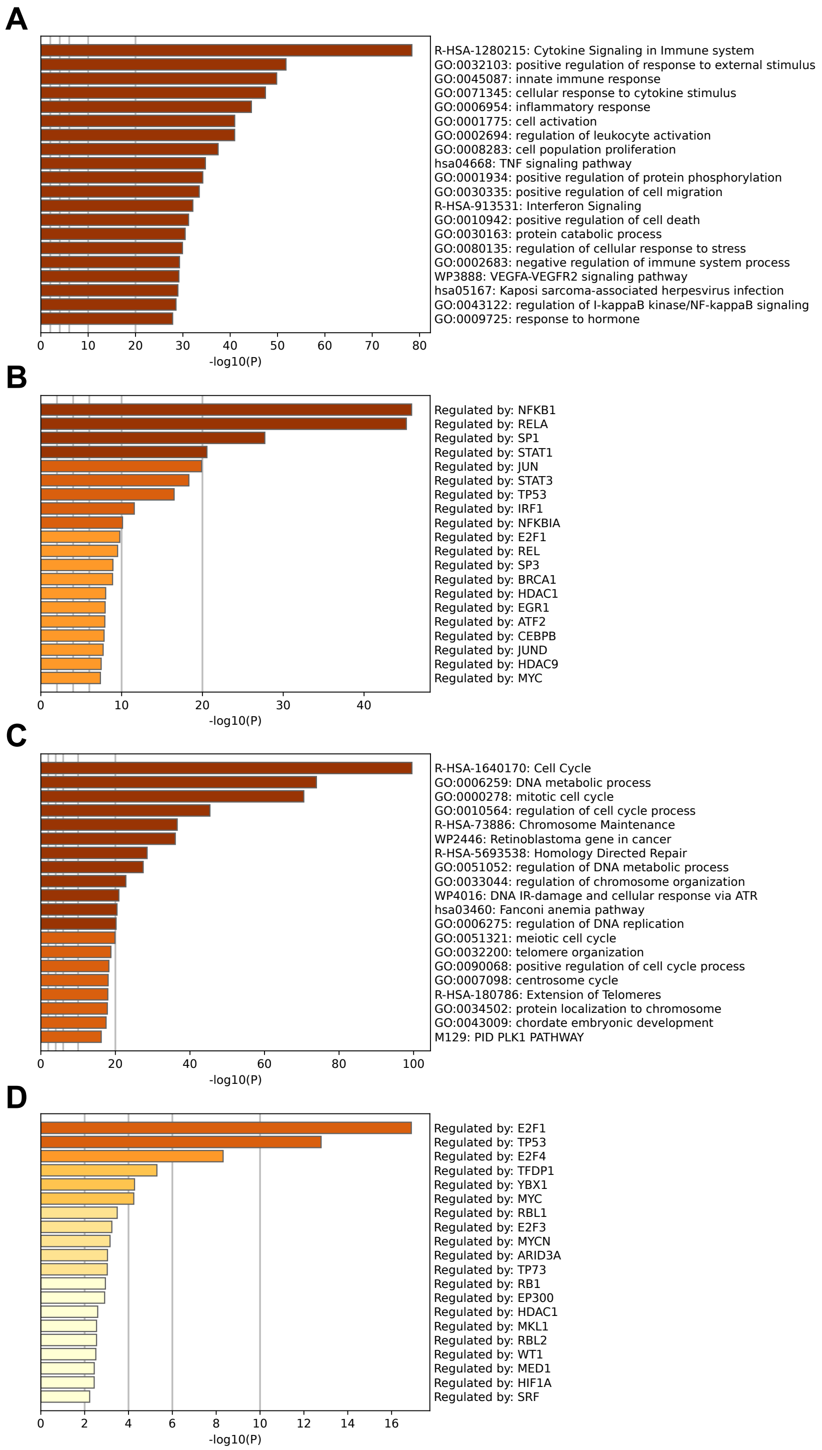

Supplemental Figure 8. Enrichment analysis of the upregulated and downregulated genes by IL-1β stimulation.

RNA-seq was performed on human chondrocytes (n = 5) treated with IL-1β (1 ng/mL) for 6 hours. Metascape enrichment (**A**) and TRRUST (**B**) analysis using the upregulated genes after IL-1β stimulation. Metascape enrichment (**C**) and TRRUST (**D**) analysis using the downregulated genes after IL-1β stimulation.

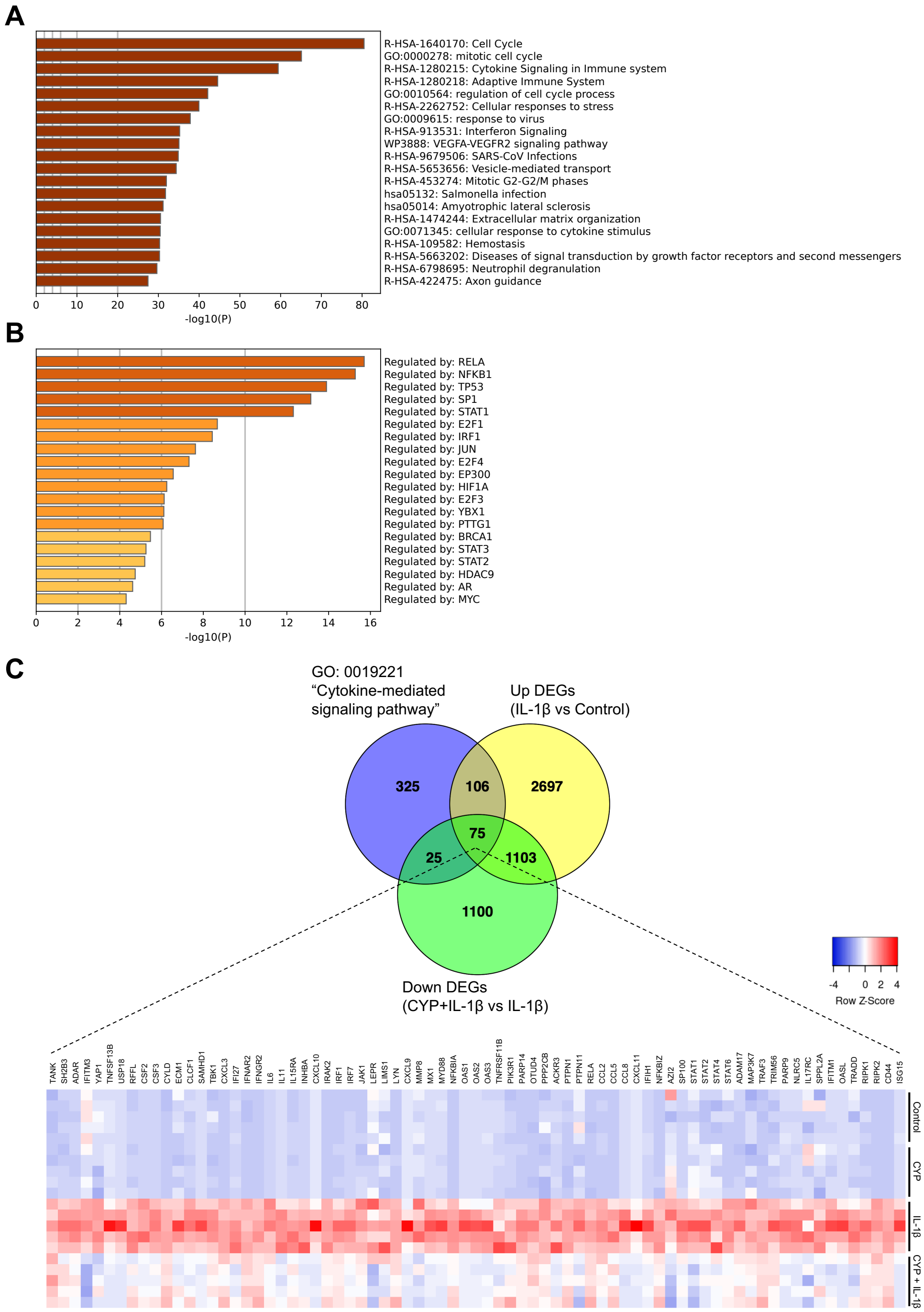

Supplemental Figure 9. Enrichment analysis of the downregulated genes by cyproheptadine treatment under IL-1β stimulation.

RNA-seq was performed on human chondrocytes (n = 5) treated with IL-1β (1 ng/mL) for 6 hours after pretreatment with or without cyproheptadine (CYP) for 24 hours. Metascape enrichment (**A**) and TRRUST (**B**) analysis using the downregulated genes by CYP treatment under IL-1β stimulation. (**C**) Venn diagram and heat map of the shared genes in GO: 0019221 "cytokine-mediated signaling pathway", the upregulated DEGs by IL-1β and the downregulated DEGs by CYP with IL-1β.

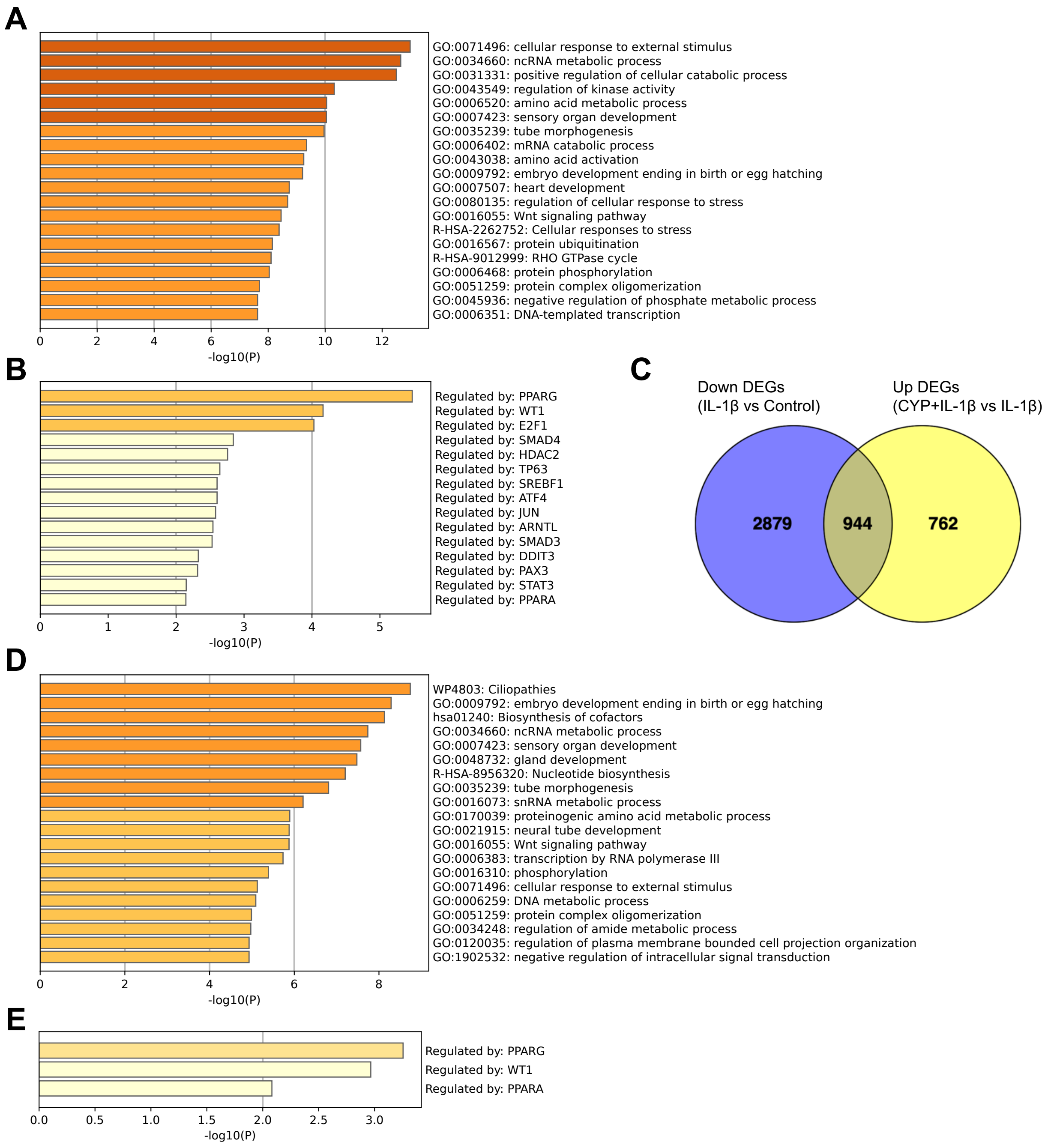

Supplemental Figure 10. Enrichment analysis of the upregulated genes by cyproheptadine treatment under IL-1β stimulation.

RNA-seq of human chondrocytes (n = 5) treated with IL-1β (1 ng/mL) for 6 hours after pretreatment with or without cyproheptadine (CYP) (30 μM) for 24 hours was performed. Metascape enrichment (**A**) and TRRUST (**B**) analysis using the upregulated genes by CYP treatment under IL-1β stimulation. (**C**) Venn diagram of the shared genes in the downregulated genes by IL-1β and the upregulated genes by CYP with IL-1β. Metascape enrichment (**D**) and TRRUST (**E**) analysis using the shared genes in (C).

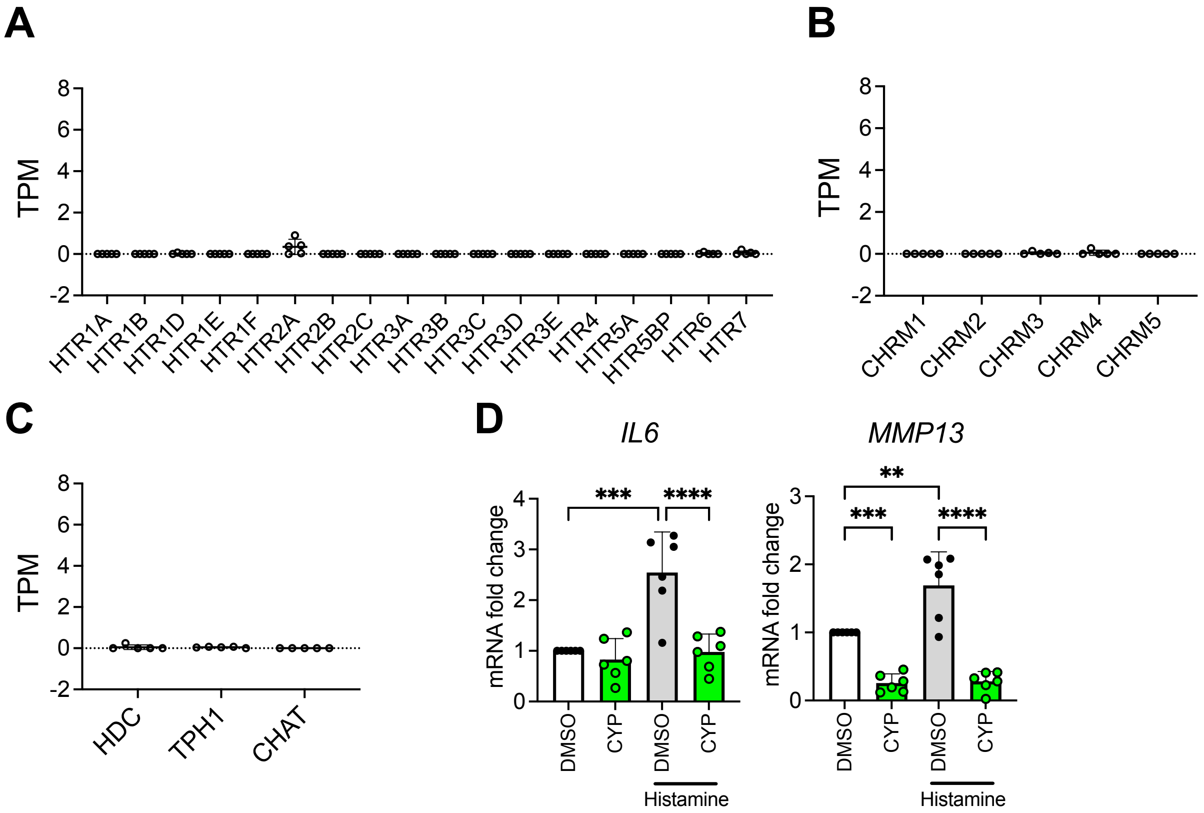

Supplemental Figure 11. Cyproheptadine signaling via histamine H1 receptor. Transcripts per kilobase million (TPM) in RNA-seq of human chondrocytes (n = 5) for serotonin receptors (**A**), muscarinic acetylcholine receptors (**B**), and HDC, TPH1 and CHAT (**C**). (**D**) Relative mRNA levels of *IL6* and *MMP13* in human synoviocytes (n = 6) incubated with histamine (10 μM) for 6 hours after pretreatment with cyproheptadine (CYP) (30 μM) for 24 hours. Data are presented as means ± SD. Statistical analysis was performed using one-way ANOVA with the Tukey-Kramer post hoc test. $^{**}P < 0.01$, $^{***}P < 0.001$, $^{****}P < 0.0001$.

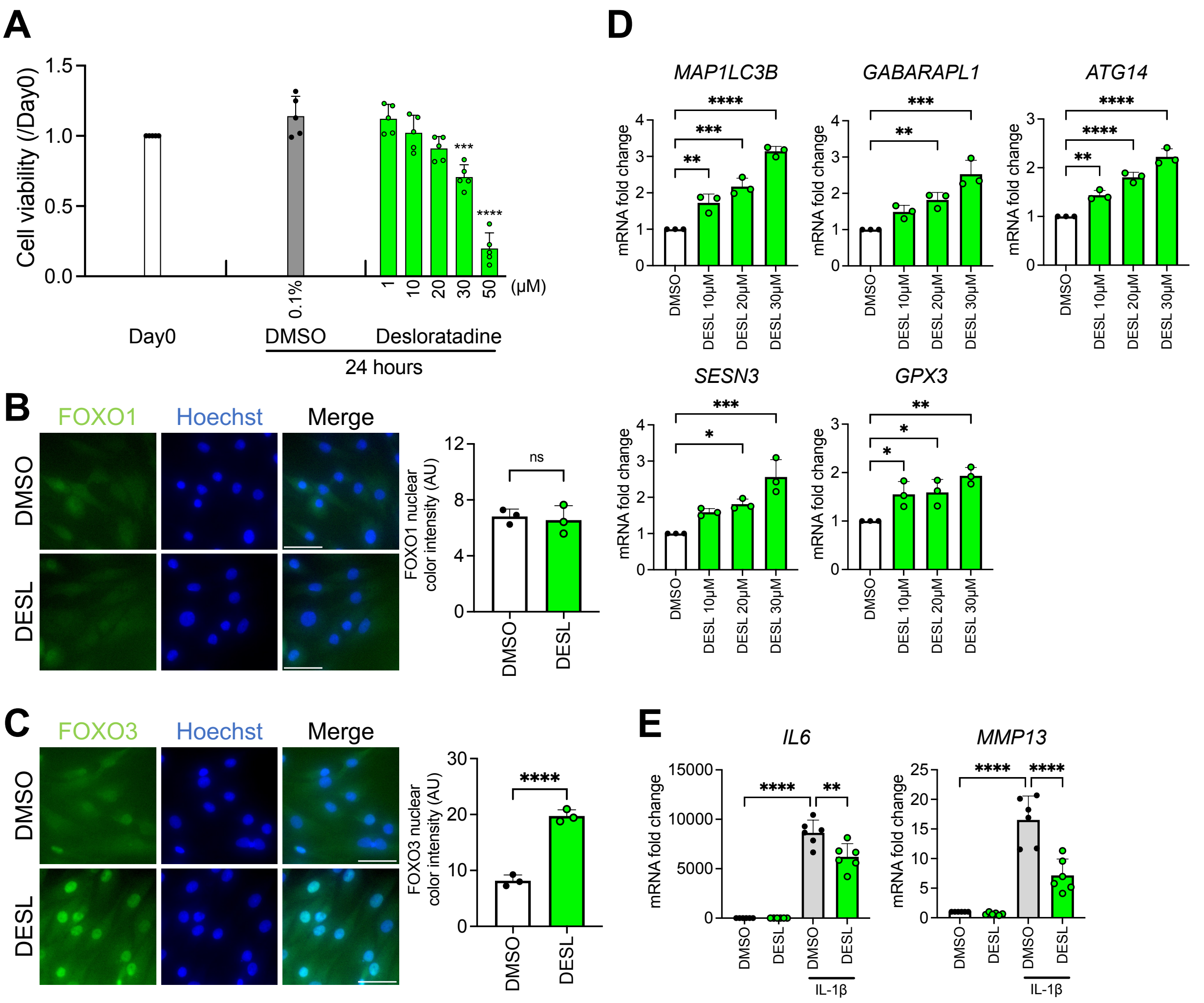

Supplemental Figure 12. Effects of desloratadine in human chondrocytes.
(**A**) Cell viabilities of human chondrocytes (n = 5) were analyzed at 0 and 24 hours after treatment with DMSO (0.1%) or desloratadine (1, 10, 20, 30, 50 μM). Data are expressed as the percent luminescence of cells in each group relative to signals at Day 0. Immunocytochemistry of FOXO1 (**B**) and FOXO3 (**C**) in human chondrocytes (n = 3) 24 hours after treatment with DMSO or desloratadine (DESL) (20 μM). Scale bar, 50 μm. (**D**) Human chondrocytes (n = 3) were incubated with the indicated doses of DESL for 24 hours and RNA was isolated for qRT-PCR for FOXO target genes. (**E**) Relative mRNA levels of *IL6* and *MMP13* in human chondrocytes (n = 6) incubated with IL-1β (1 ng/mL) for 6 hours after pretreatment with or without DESL (20 μM) for 24 hours in qRT-PCR. Data are presented as means ± SD. Statistical analysis in (A) and (D) was performed using one-way ANOVA with the Dunnett's post hoc test. Statistical analysis in (B) and (C) was performed using Student's t-test. Statistical analysis in (E) was performed using one-way ANOVA with the Tukey-Kramer post hoc test. *P < 0.05, **P < 0.01, ***P < 0.001, ****P < 0.0001.

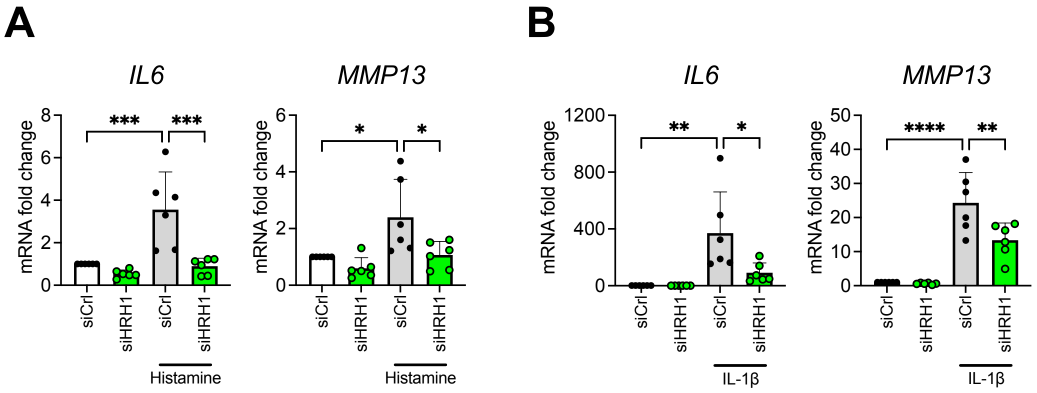

Supplemental Figure 13. Anti-inflammatory effects of HRH1 knockdown in human synoviocytes. Human synoviocytes (n = 6) were incubated with histamine (10 μM) (**A**) or IL-1β (1 ng/mL) (**B**) for 6 hours after siRNA transfection. *IL6* and *MMP13* were measured by qRT-PCR. Data are presented as means ± SD. Statistical analysis was performed using one-way ANOVA with the Tukey-Kramer post hoc test. **P* < 0.05, ***P* < 0.01, ****P* < 0.001, *****P* < 0.0001.

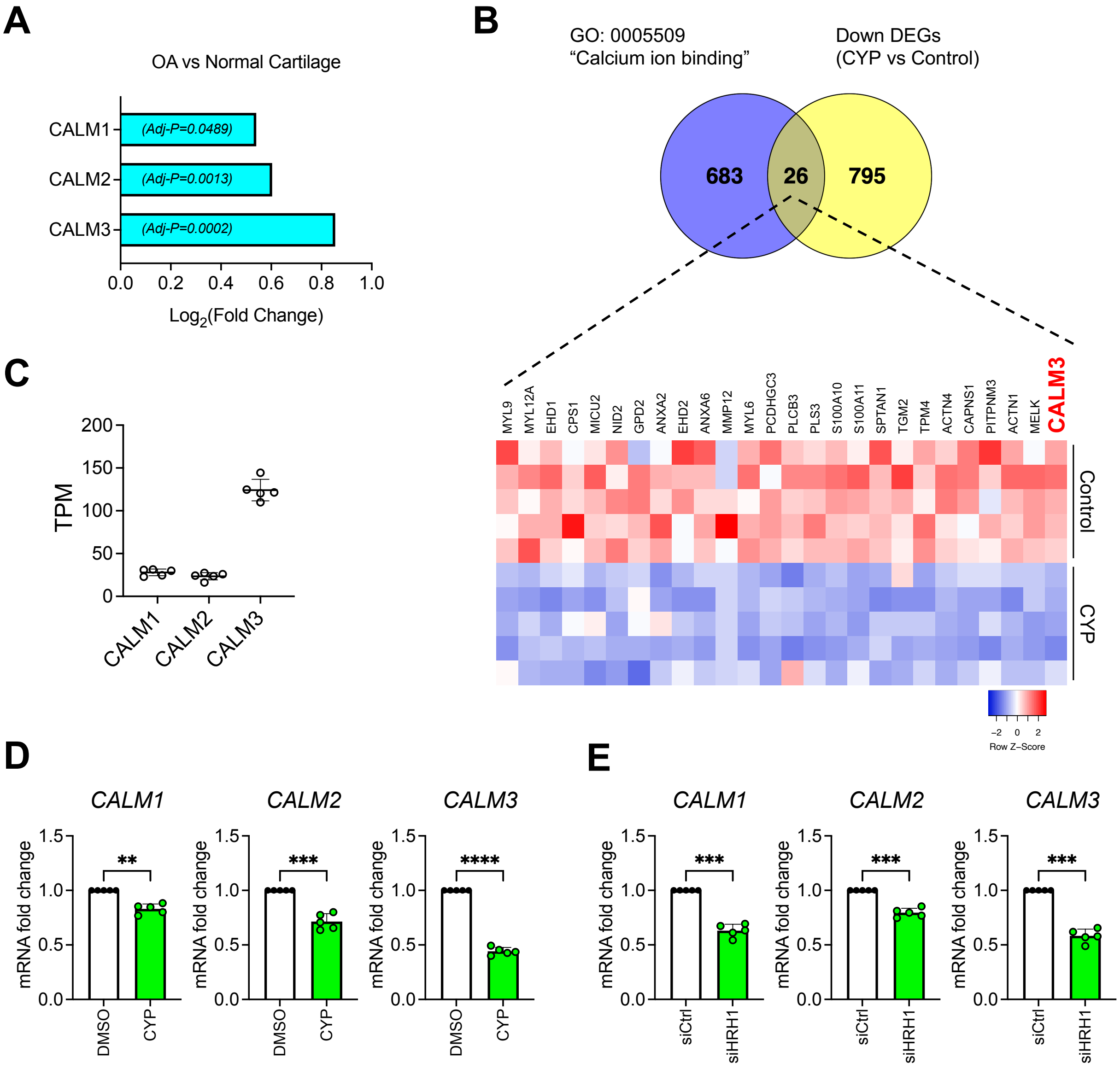

Supplemental Figure 14. Effects of cyproheptadine on calmodulin gene expression. (**A**) Enhanced CALMs gene expression in OA cartilage compared to normal cartilage (Fisch et al., 2018 (31)). (**B**) Venn diagram and heat map of the shared genes in GO: 0005509 "calcium ion binding" and the downregulated DEGs in chondrocytes treated with CYP. (**C**) TPM in RNA-seq of chondrocytes (n = 5) for CALMs. Relative mRNA levels of *CALM1*, *CALM2* and *CALM3* in human chondrocytes (n = 5) treated with CYP (**D**) or transfected with siCtrl or siHRH1 (**E**) in qRT-PCR. Data are presented as means ± SD. Statistical analysis was performed using Student's t-test. $^{**}P < 0.01$, $^{***}P < 0.001$, $^{****}P < 0.0001$.

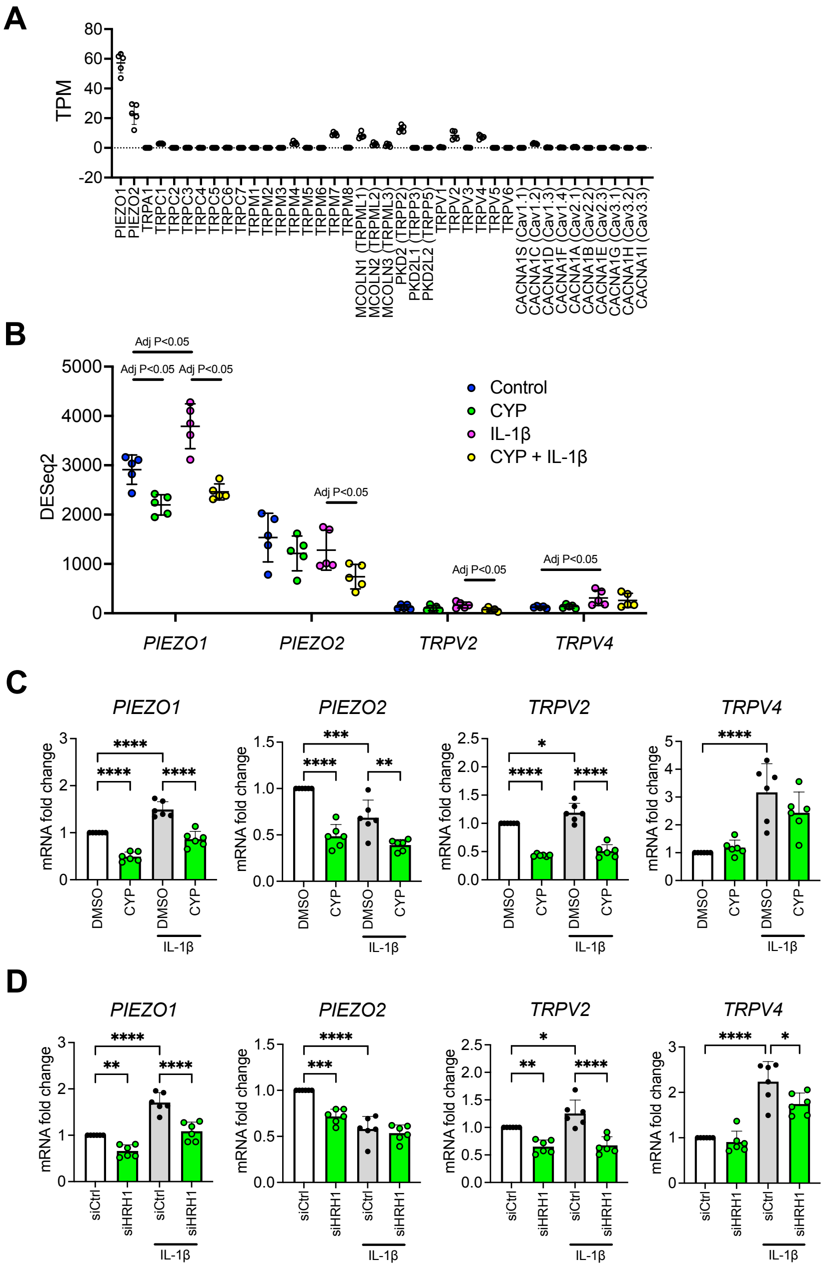

Supplemental Figure 15. Effects of cyproheptadine on gene expression of mechanosensitive channels.

(**A**) TPM in RNA-seq of human chondrocytes (n = 5) for calcium ion channels. (**B**) DeSeq2 normalized read counts for *PIEZO1*, *PIEZO2*, *TRPV2* and *TRPV4* in RNA-seq of chondrocytes (n = 5) incubated with IL-1β (1 ng/mL) for 6 hours after pretreatment with or without cyproheptadine (CYP) (30 μM). Adjusted P values (Adj P) < 0.05 were indicated. Relative mRNA levels of *PIEZO1*, *PIEZO2*, *TRPV2* and *TRPV4* in chondrocytes (n = 6) incubated with IL-1β (1 ng/mL) for 6 hours after pretreatment with or without CYP (**C**) or transfection with siCtrl or siHRH1 (**D**) in qRT-PCR. Data are presented as means ± SD. Statistical analysis was performed using one-way ANOVA with the Tukey-Kramer post hoc test. **P* < 0.05, ***P* < 0.01, ****P* < 0.001, *****P* < 0.0001.

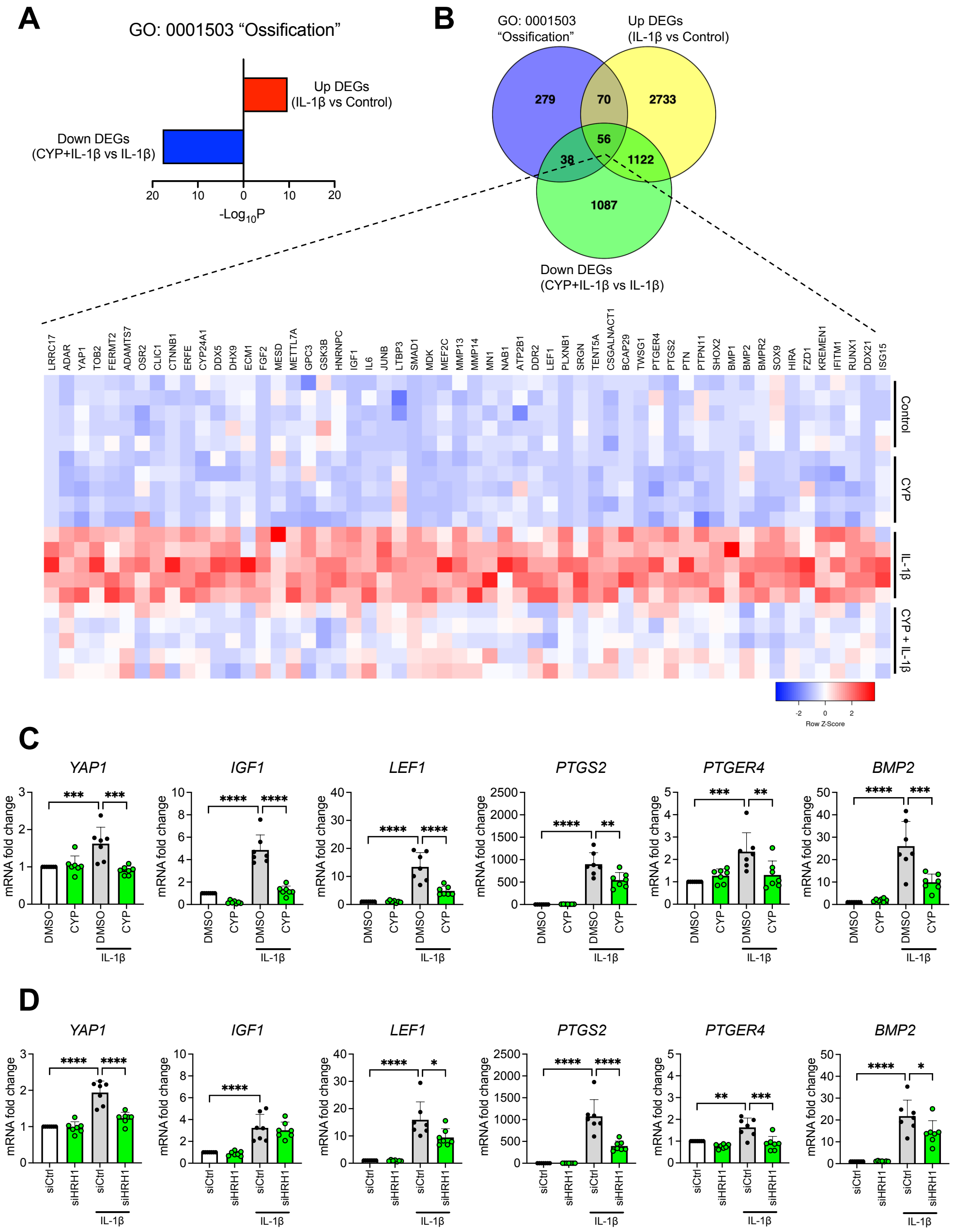

Supplemental Figure 16. Effects of HRH1 signaling on the expression of ossification-related genes.

(**A**) Enriched GO: 0001503 “ossification” in the upregulated DEGs by IL-1β and the downregulated DEGs by cyproheptadine (CYP) with IL-1β in RNA-seq. (**B**) Venn diagram and heat map of the shared genes in GO: 0001503 “ossification”, the upregulated DEGs by IL-1β and the downregulated DEGs by CYP with IL-1β. Relative mRNA levels of ossification-related genes in chondrocytes (n = 7) incubated with IL-1β (1 ng/mL) for 6 hours after pretreatment with or without cyproheptadine (CYP) (30 μM) for 24 hours (**C**) or siHRH1 transfection (**D**) in qRT-PCR. Data are presented as means ± SD. Statistical analysis was performed using one-way ANOVA with the Tukey-Kramer post hoc test. *P < 0.05, **P < 0.01, ***P < 0.001, ****P < 0.0001.

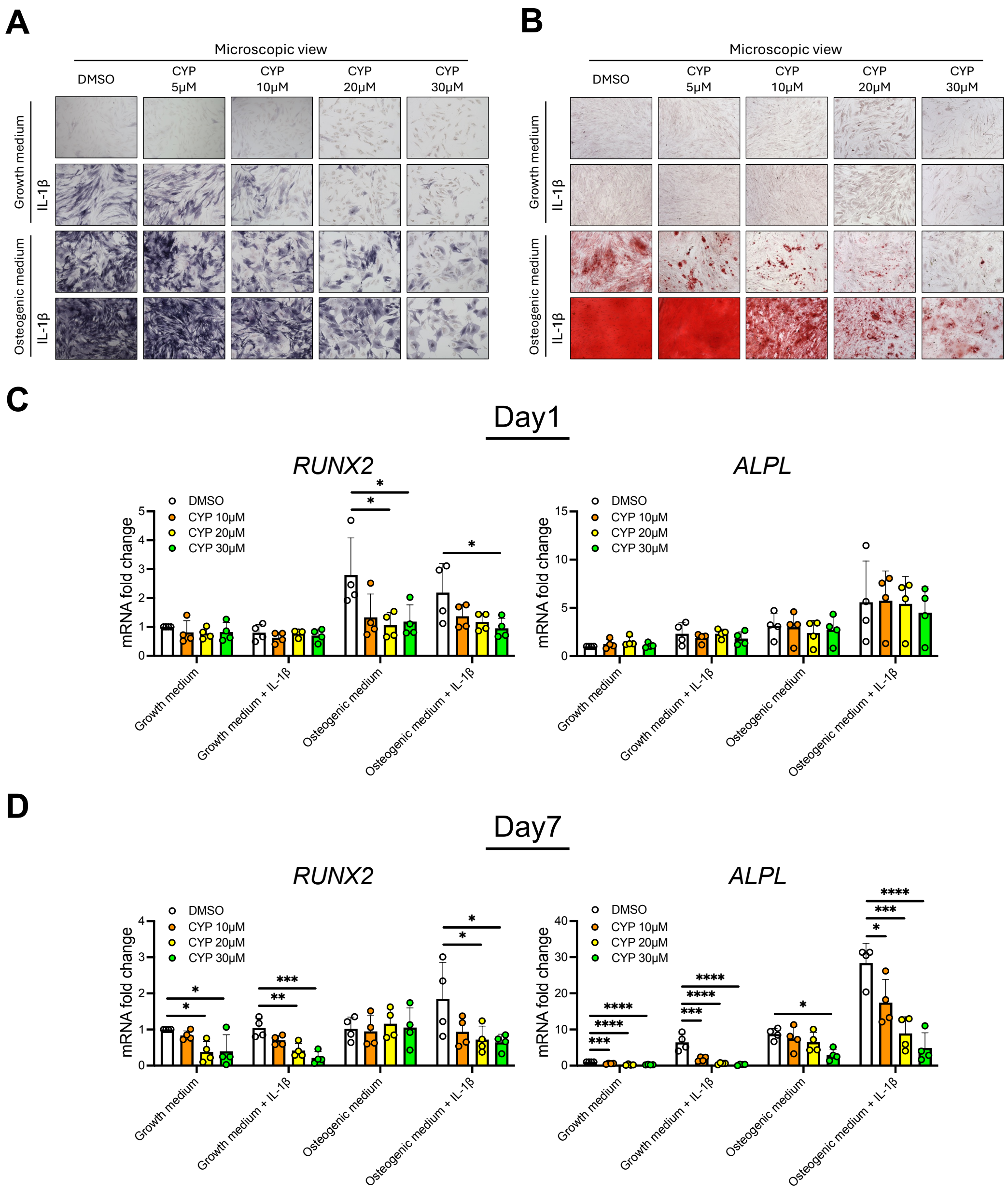

Supplemental Figure 17. Effects of cyproheptadine on osteogenesis.
(**A**) Microscopic view of ALP staining in human mesenchymal stem cells (MSCs) (n = 4) incubated in growth medium or osteogenic medium in the presence or absence of CYP (5, 10, 20 or 30 μM) and IL-1β (1 ng/mL) for 7 days. (**B**) Microscopic view of Alizarin red S (ARS) staining in MSCs (n = 4) incubated in growth medium or osteogenic medium in the presence or absence of CYP (5, 10, 20 or 30 μM) and IL-1β (1 ng/mL) for 28 days. Relative mRNA levels of *RUNX2* and *ALPL* in human mesenchymal stem cells (MSCs) (n = 4) incubated in growth medium or osteogenic medium in the presence or absence of cyproheptadine (CYP) (10, 20 or 30 μM) and IL-1β (1 ng/mL) for 1 day (**C**) or 7 days (**D**) in qRT-PCR. Data are presented as means ± SD. Statistical analysis was performed using one-way ANOVA with the Dunnett's post hoc test. *P < 0.05, **P < 0.01, ***P < 0.001, ****P < 0.0001.

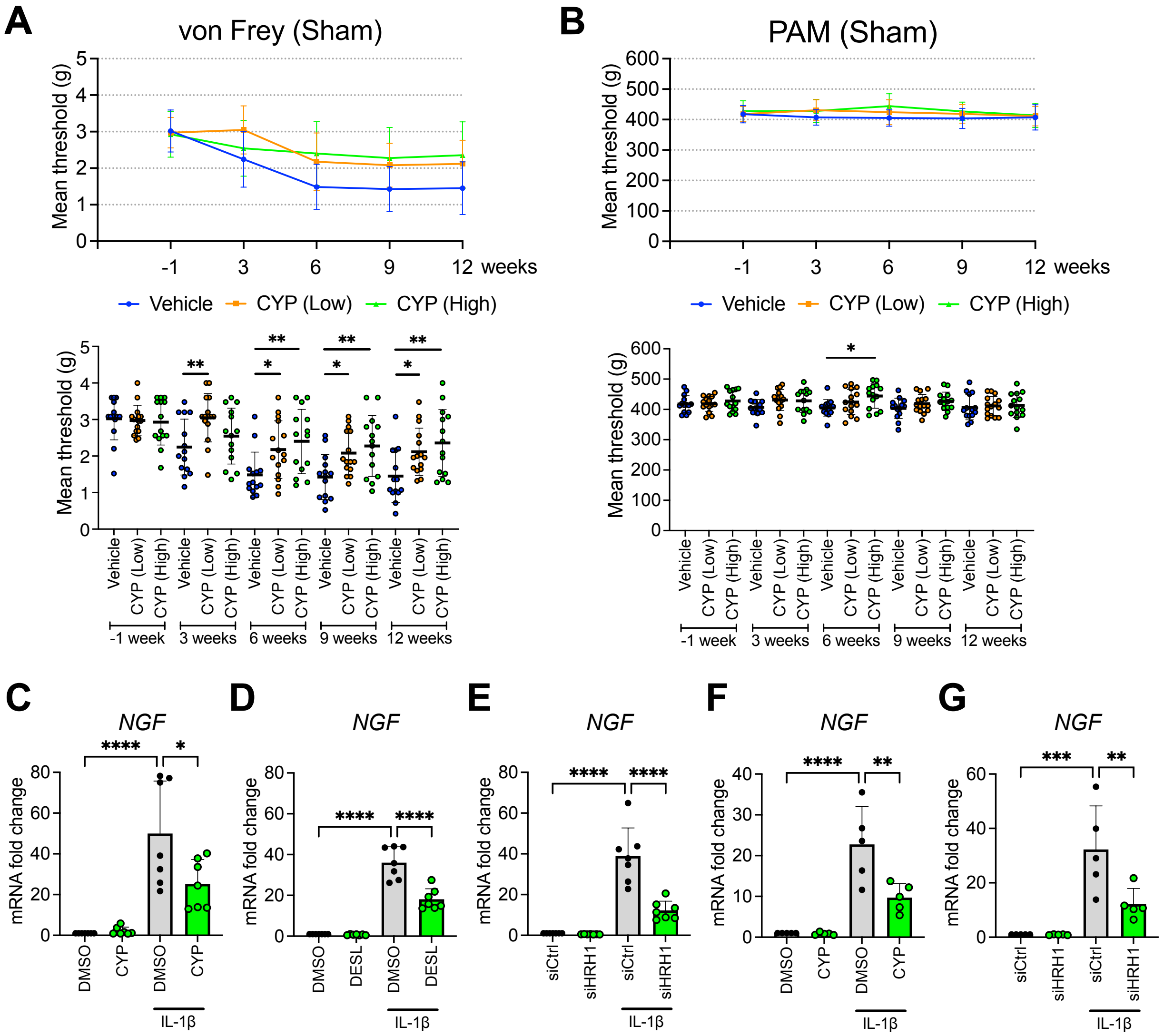

Supplemental Figure 18. Effects of cyproheptadine on pain behaviors.
Pain behaviors were evaluated by von Frey test (**A**) and pressure application measurement (PAM) (**B**) in mice treated with cyproheptadine (CYP) (low dose: 5mg/kg, high dose: 10mg/kg) or control vehicle after Sham surgery (Figure 2A). Sham vehicle (n = 14), Sham CYP (Low) (n = 15), Sham CYP (High) (n = 14). Relative mRNA levels of *NGF* in chondrocytes (n = 7) incubated with IL-1β (1 ng/mL) for 6 hours after pretreatment with CYP (**C**), desloratadine (DESL) (**D**) or siHRH1 transfection (**E**). Relative mRNA levels of *NGF* in synoviocytes (n = 5) incubated with IL-1β (1 ng/mL) for 6 hours after pretreatment with CYP (**F**) or siHRH1 transfection (**G**). Statistical analysis in (A) and (B) was performed using one-way ANOVA with the Dunnett's post hoc test. Statistical analysis in (C to G) was performed using one-way ANOVA with the Tukey-Kramer post hoc test. *$P < 0.05$, **$P < 0.01$, ***$P < 0.001$, ****$P < 0.0001$.

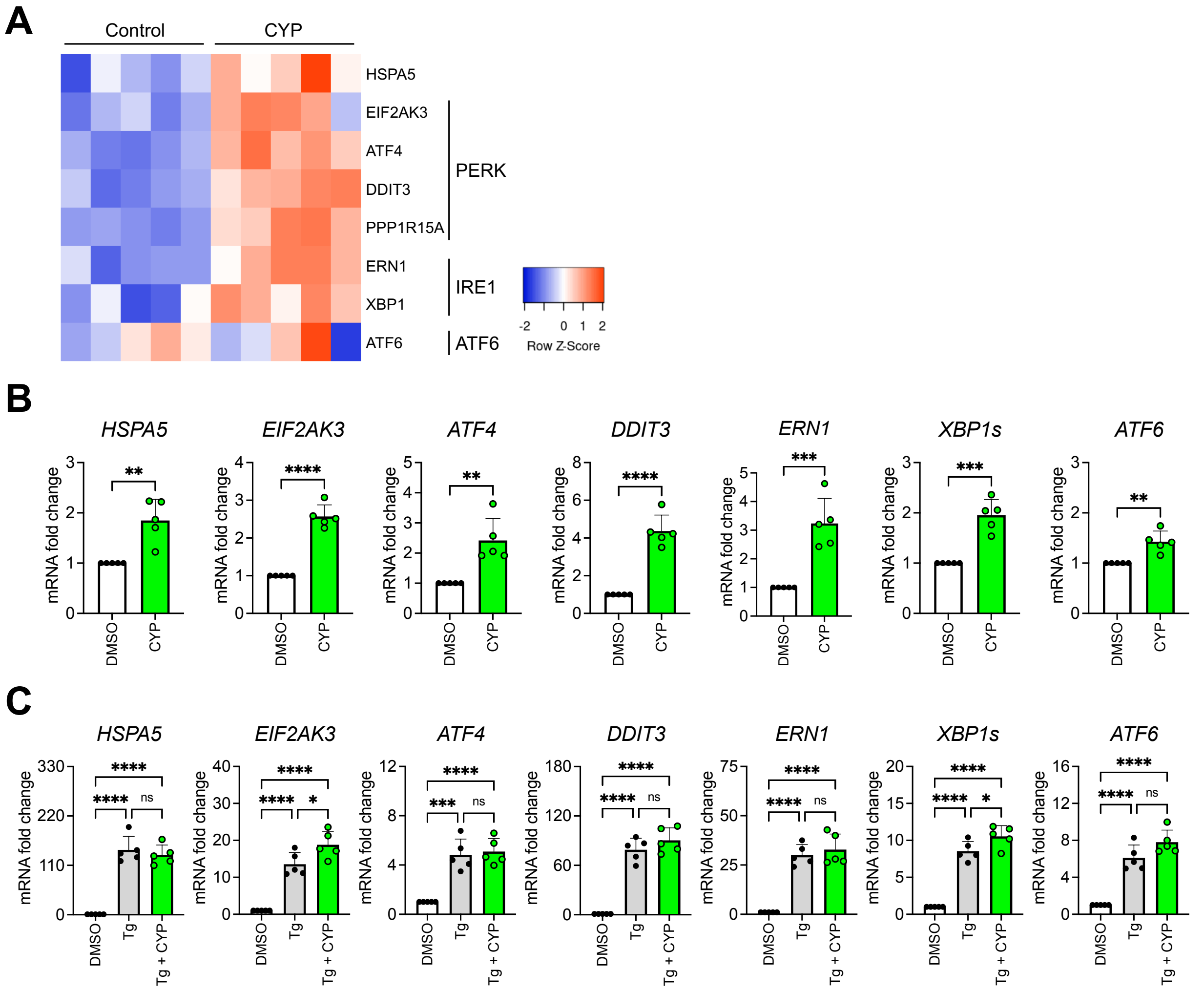

Supplemental Figure 19. Effects of cyproheptadine on the expression of UPR-related genes.

(**A**) Heatmap of UPR-related genes in RNA-seq in human chondrocytes (n = 5) treated with cyproheptadine (CYP) (30 μM). (**B**) Relative mRNA of UPR-related genes in human chondrocytes (n = 5) treated with CYP in qRT-PCR. (**C**) Relative mRNA of UPR-related genes in human chondrocytes (n = 5) treated with thapsigargin (Tg) with or without CYP in qRT-PCR. Data are presented as means ± SD. Statistical analysis in (B) was performed using Student's t-test. Statistical analysis in (C) was performed using one-way ANOVA with the Tukey-Kramer post hoc test. *P < 0.05, **P < 0.01, ***P < 0.001, ****P < 0.0001.

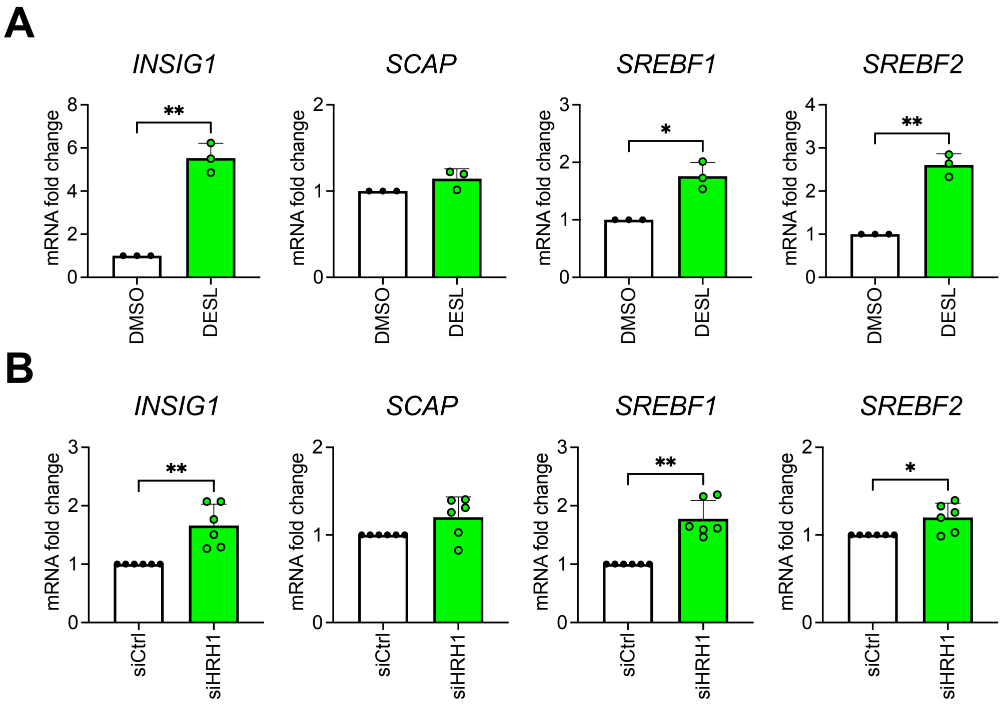

Supplemental Figure 20. Effects of HRH1 signaling on the expression of lipid and cholesterol biosynthesis-related genes.
(**A**) Relative mRNA levels of *INSIG1*, *SCAP*, *SREBF1* and *SREBF2* in human chondrocytes (n = 3) incubated with desloratadine (DESL) (20 μM) for 24 hours in qRT-PCR. (**B**) Relative mRNA levels of *INSIG1*, *SCAP*, *SREBF1* and *SREBF2* in human chondrocytes (n = 6) transfected with siCtrl or siHRH1 in qRT-PCR. Data are presented as means ± SD. Statistical analysis was performed using Student's t-test. *$P < 0.05$, **$P < 0.01$.

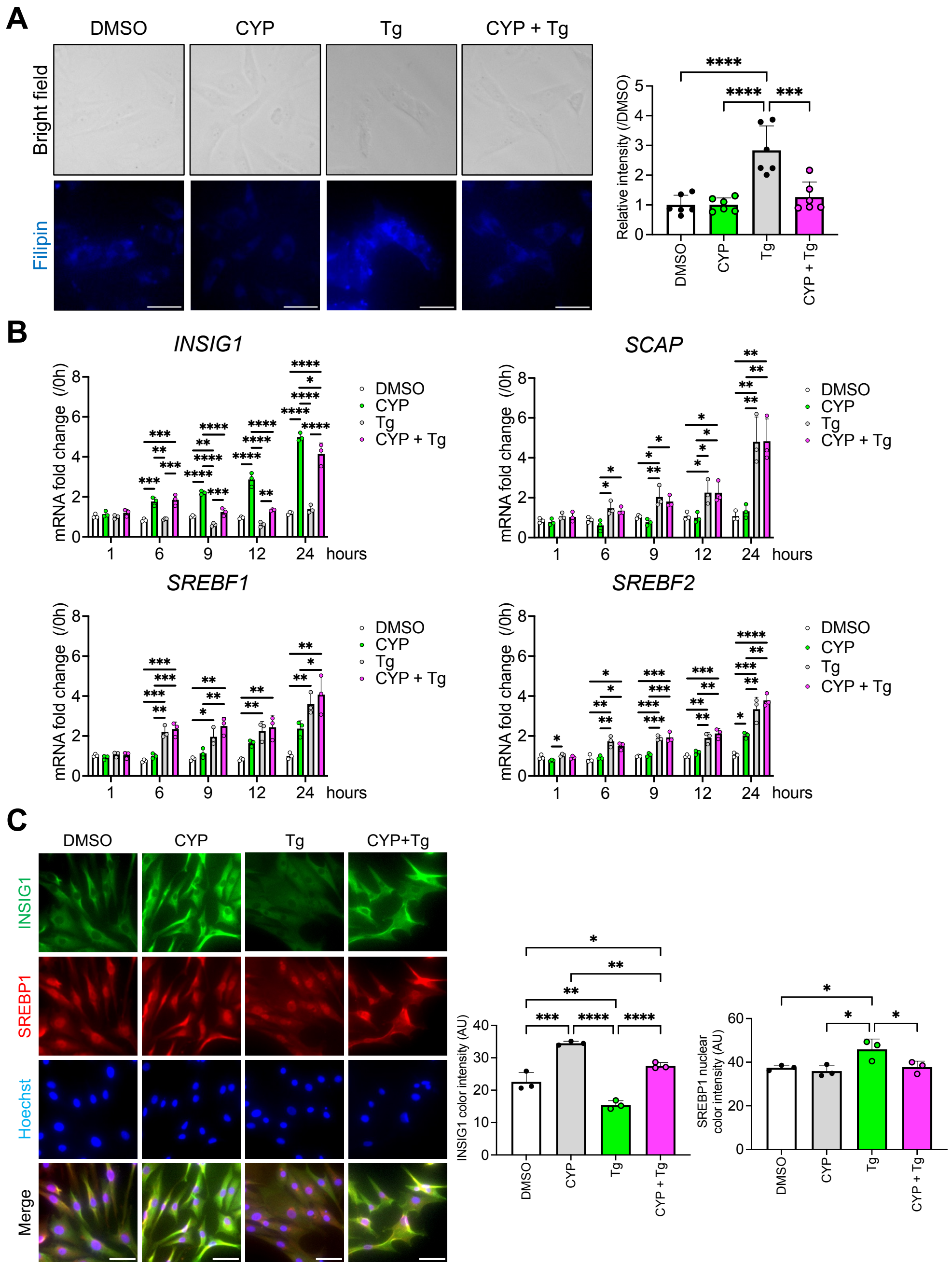

Supplemental Figure 21. Cyproheptadine regulation of ER stress-induced lipid/cholesterol biosynthesis.

(**A**) Filipin staining (n = 6) in chondrocytes incubated with CYP, Tg or CYP and Tg for 24 hours. Scale bar, 50 μm. (**B**) Relative mRNA levels of *INSIG1*, *SCAP*, *SREBF1* and *SREBF2* in chondrocytes (n = 3) incubated with CYP, Tg or CYP and Tg for 24 hours in qRT-PCR. (**C**) Immunocytochemistry of INSIG1 (green) and SREBP1 (red) in human chondrocytes (n = 3) incubated with CYP, Tg or CYP and Tg for 24 hours. Scale bar, 50 μm. Data are presented as means ± SD. Statistical analysis was performed using one-way ANOVA with the Tukey-Kramer post hoc test. *P < 0.05, **P < 0.01, ***P < 0.001, ****P < 0.0001.

Supplemental Table 1. Summary of drugs which have similar effects with FOXO3 overexpression in the in-silico screening.

Target	# of Hits	Compound
PI3K/AKT	12	TGX-221, BEZ235, PI-103, Wortmannin, PI-828, KU-0060648, AKT-inhibitor-1-2, PIK-90, MK-2206, ZSTK-474, AZD-6482, AKT-inhibitor-IV
mTOR	9	AZD-8055, Rapamycin, BEZ235, WYE-354, KU-0063794, Temsirolimus, WYE-125132, OSI-027, Torin-1
Serotonin receptor	6	Cyproheptadine, Anpirtoline, Spiramide, Ondansetron, Thioridazine, Perphenazine
Histamine receptor	4	Cyproheptadine, Iodophenpropit, Perphenazine, Carbinoxamine
HDAC	4	Apicidin, ISOX, Panobinostat, Tubastatin A
IGF1/Insulin receptor	3	BMS-754807, BMS-536924, Linsitinib
Actin polymerization	3	Cytochalasin B, Latrunculin B, Cytochalasin D

Supplemental Table 2. Sequences of primers for qRT-PCR.

Gene	Forward (5'-3')	Reverse (5'-3')
Human *GAPDH*	AGCCACATCGCTCAGACAC	GCCCAATACGACCAAATCC
Human *FOXO1*	AAGAGCGTGCCCTACTTCAA	CTGTTGTTGTCCATGGATGC
Human *FOXO3*	GCAAGCACAGAGTTGGATGA	CAGGTCGTCCATGAGGTTTT
Human *MAP1LC3B*	GAGAAGACCTTCAAGCAGCG	AAGCTGCTTCTCACCCTTGT
Human *GABARAPL1*	TCCGGAAGAGAATCCACCTG	GTCTTCCTCATGATTGTCCTCATAC
Human *ATG14*	GGACTCCGTGGACGATGC	CTTGTCGATAAACCTCTCCCG
Human *SESN3*	CGGTCGTCTGGACAACATCA	GATGTCTAGCTGCAGCCATTATT
Human *GPX3*	AGAAGTCGAAGATGGACTGCC	TGGTCGGACATACTTGAGGGT
Human *IL6*	GGTACATCCTCGACGGCATCT	GTGCCTCTTTGCTGCTTTCAC
Human *MMP13*	GTCTCTCTATGGTCCAGGAGATGAA	AGGCGCCAGAAGAATCTGT
Human *CALM1*	ATCAGCTGACCGAAGAACAGA	AGTGACCTCATGACAGTTCCAA
Human *CALM2*	GGCTGACCAACTGACTGAAGA	GTGCCATTACCATCAGCATCTAC
Human *CALM3*	TAGGCTGACCAGCTGACTGA	CCAACTCCTTGGTGGTGATAGT
Human *PIEZO1*	GCCGTCACTGAGAGGATGTTC	TGGAAGAGGAAGAGGTTGAGATGA
Human *PIEZO2*	TGACAGTATCTCCAGCTGCTAC	GAGGAGGACATATCCATGCTGAG
Human *TRPV2*	GGCCAAGAAGATGGCTCTGA	GAAGAGCCGATCTCGGTCAA
Human *TRPV4*	CCTGTTCTTCTTCACCAACATCAA	TGAGACGATCACCAGGACAGA
Human *YAP1*	GTGGATGAGATGGATACAGGTGATA	TCCTGCAGACTTGGCATCAG
Human *IGF1*	TGTGACATTGCTCTCAACATCTC	TGGTGTGCATCTTCACCTTCA
Human *LEF1*	CCAAGAATGACAGCTGCCTACA	AACAAGCTTCCATCTCCAGAAGAG
Human *PTGS2*	CTGGTGCCTGGTCTGATGA	GCCACTCAAGTGTTGCACATA
Human *PTGER4*	CCAGATGGTCATCTTACTCATTGC	AACTTGGCTGATATAACTGGTTGAC
Human *BMP2*	CGCAGCTTCCACCATGAAG	CTGAAGCTCTGCTGAGGTGATA
Human *RUNX2*	CCGGAATGCCTCTGCTGTTA	AGCTTCTGTCTGTGCCTTCTG
Human *ALPL*	CCTCGTTGACACCTGGAAGAG	TCCGTCACGTTGTTCCTGTT
Human *NGF*	GGCCACACTGAGGTGCATAG	AGGGCAGTGTCAAGGGAATG
Human *HSPA5*	GGAACCATCCCGTGGCATAA	TGGTAGGCACCACTGTGTTC
Human *EIF2AK3*	GCCTTAGCCAAGCTTGAACAC	GGCCAGTCTGTGCTTTCATC
Human *ATF4*	TGTGGATGGGTTGGTCAGTC	ACAGGGCATCCAAGTCGAAC
Human *DDIT3*	GGAGCTGGAAGCCTGGTATG	GCAGGGTCAAGAGTGGTGAA
Human *ERN1*	ACCAGCGTGGTGATAGTTGG	CTCACGGTCTGCGAAGCTAA
Human *XBP1s*	AGGCGCTGAGGAGGAAACT	CCCAAGCGCTGTCTTAACTC
Human *ATF6*	AGTATCAGGAACTCAGGGAGTG	GTAGCTGGTAACAGCAGGTGA
Human *INSIG1*	CTCGTGCTCTTCTCGGTTGG	CGAGGTGACTGTCGATACAGG
Human *SCAP*	TGCATCTTAGCCTGCTGCTAC	CACTCAGGCTGCTCAGTAGG
Human *SREBF1*	GCTGACCGACATCGAAGACAT	GGCTTCAAGAGAGGAGCTCAA
Human *SREBF2*	GCTGGCTTCTCTCCCTACTC	GAAGAATCCGTGAGCGGTCTA

Supplemental Videos

Supplemental Video 1. Intracellular calcium level after histamine treatment.

Response to histamine (10 μM) stimulation in TC28 cells pretreated with DMSO for 1 hour. Green fluorescence indicates the change of intracellular calcium levels. 50x speed.

Supplemental Video 2. Intracellular calcium level after histamine treatment following cyproheptadine pretreatment.

Response to histamine (10 μM) stimulation in TC28 cells pretreated with cyproheptadine (30 μM) for 1 hour. Green fluorescence indicates the change of intracellular calcium levels. 50x speed.

Supplemental Video 3. Intracellular calcium level after DMSO treatment.

Response to DMSO in TC28 cells. Green fluorescence indicates the change of intracellular calcium levels. 50x speed.

Supplemental Video 4. Intracellular calcium level after cyproheptadine treatment.

Response to cyproheptadine (30 μM) in TC28 cells. Green fluorescence indicates the change of intracellular calcium levels. 50x speed.

Supplemental Video 5. Intracellular calcium level after thapsigargin treatment.

Response to thapsigargin (1 μM) stimulation in TC28 cells pretreated with DMSO for 1 hour. Green fluorescence indicates the change of intracellular calcium levels. 50x speed.

Supplemental Video 6. Intracellular calcium level after thapsigargin treatment following cyproheptadine pretreatment.

Response to thapsigargin (1 μM) stimulation in TC28 cells pretreated with cyproheptadine (30 μM) for 1 hour. Green fluorescence indicates the change of intracellular calcium levels. 50x speed.

Supplemental References

1. Glasson SS, et al. The surgical destabilization of the medial meniscus (DMM) model of osteoarthritis in the 129/SvEv mouse. *Osteoarthritis Cartilage.* 2007;15(9):1061-1069.
2. Chaplan SR, et al. Quantitative assessment of tactile allodynia in the rat paw. *J Neurosci Methods.* 1994;53(1):55-63.
3. Courties A, et al. Human-specific duplicate CHRFAM7A gene is associated with more severe osteoarthritis and amplifies pain behaviours. *Ann Rheum Dis.* 2023;82(5):710-718.
4. Pritzker KPH, et al. Osteoarthritis cartilage histopathology: grading and staging. *Osteoarthritis Cartilage.* 2006;14(1):13-29.
5. Krenn V, et al. Grading of chronic synovitis -- a histopathological grading system for molecular and diagnostic pathology. *Pathol Res Pract.* 2002;198(5):317-325.
6. Nagira K, et al. Histological scoring system for subchondral bone changes in murine models of joint aging and osteoarthritis. *Sci Rep.* 2020;10(1):10077.
7. Zhou Y, et al. Metascape provides a biologist-oriented resource for the analysis of systems-level datasets. *Nat Commun.* 2019;10(1):1523.
