# Supplementary figures and images for: Histamine H1 receptor inverse agonists improve structure and pain in an osteoarthritis mouse model
Source: J Clin Invest. 2025 Aug 28;135(21):e183588. doi: 10.1172/JCI183588 (PMC12578390; doi:10.1172/JCI183588)

**Figure 3F**

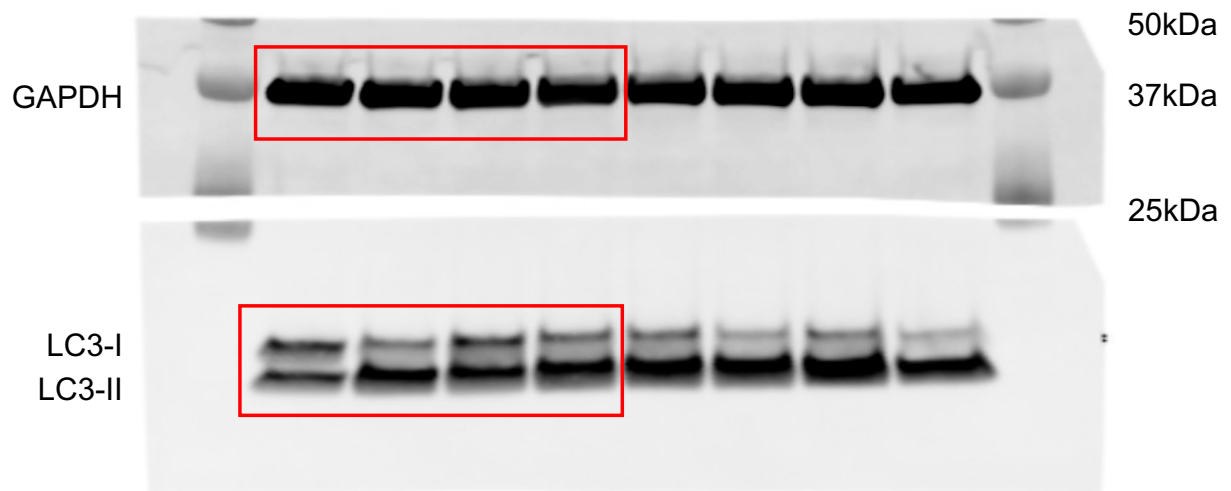

**Figure 4H**

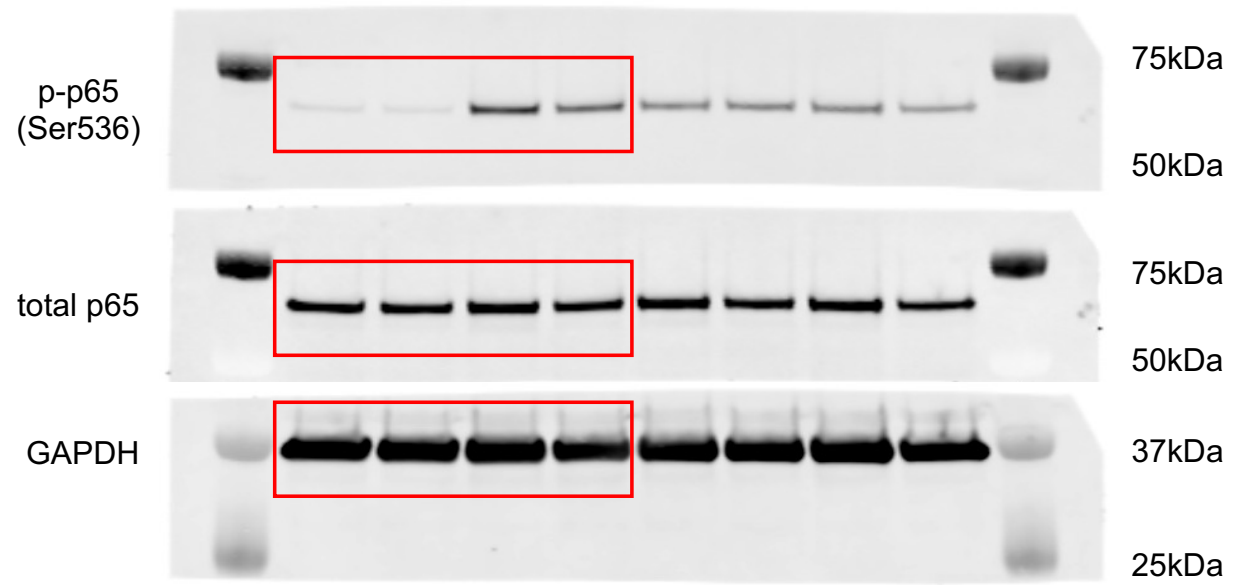

### Figure 7B

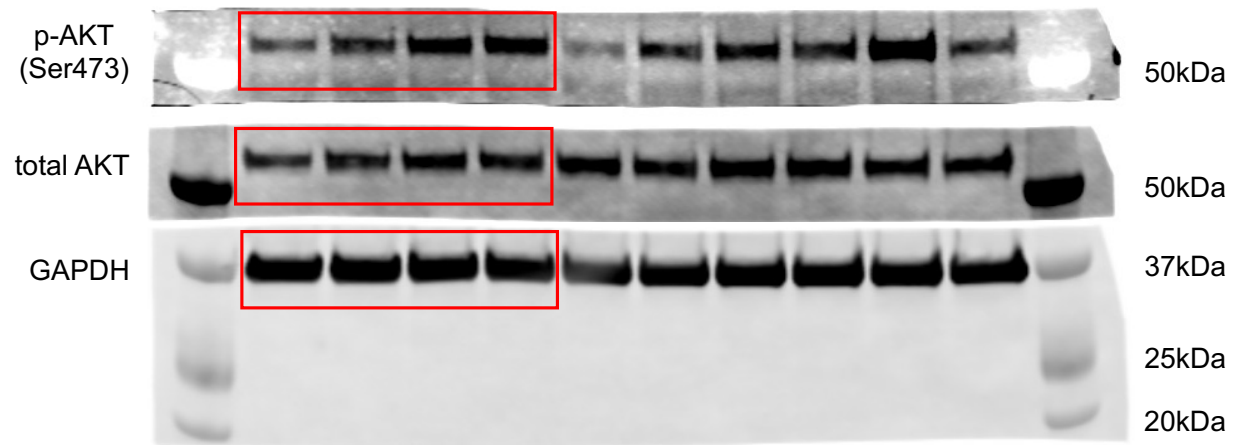

**Figure 7D**

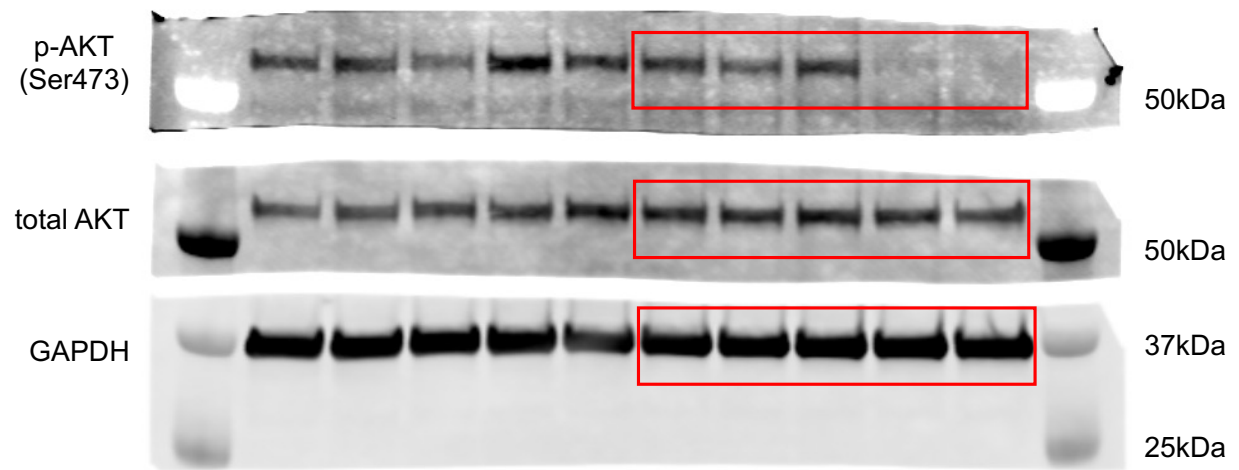

**Figure 8D**

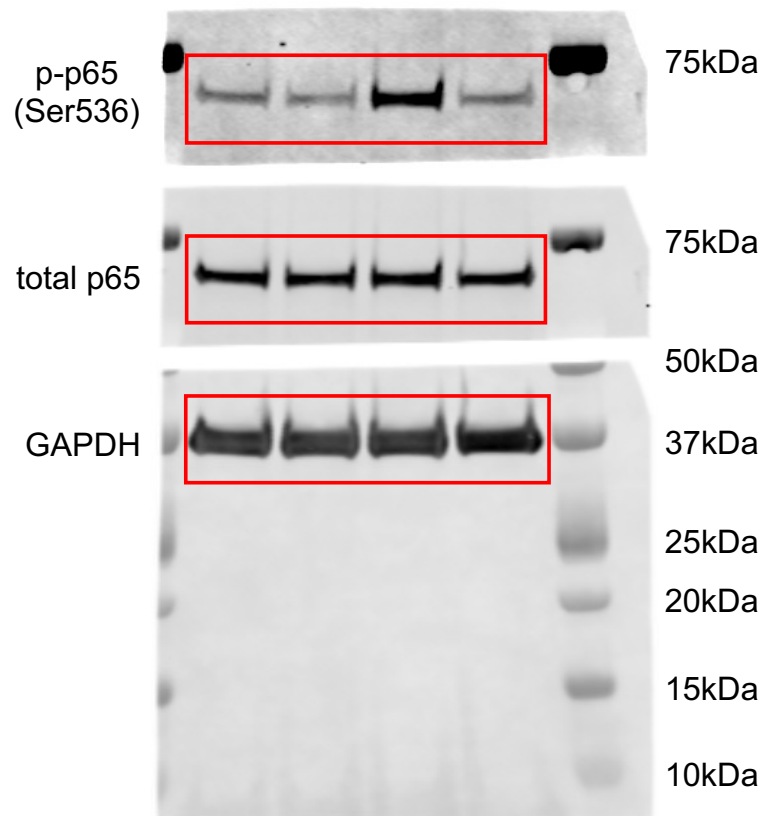

Supplement: Unedited blot and gel images [file jci-135-183588-s175.pdf]
